# Multimodal AI‐Driven Identification of Dehydrocostus Lactone as a Potent Renal Fibrosis Attenuator Targeting IQGAP1

**DOI:** 10.1002/advs.202520277

**Published:** 2026-02-03

**Authors:** Weijiang Lin, Wenzhuo Xu, Kang Liu, Ping Wang, Zhenzhen Zhu, Wenyu Lu, Zhe Zheng, Xiaoqian Peng, Xunkai Yin, Shulan Mei, An Pan, Jian Liu, Lihong Hu

**Affiliations:** ^1^ Jiangsu Key Laboratory for Functional Substance of Chinese Medicine School of Pharmacy Nanjing University of Chinese Medicine Nanjing P. R. China; ^2^ Department of Nephrology the First Affiliated Hospital of Nanjing Medical University (Jiangsu Province Hospital) Nanjing Medical University Nanjing P. R. China; ^3^ School of Artificial Intelligence and Information Technology Nanjing University of Chinese Medicine Nanjing P. R. China; ^4^ China Joint Graduate School of Traditional Chinese Medicine Nanjing P. R. China

**Keywords:** AI‐driven, Aucklandiae Radix, CCT3, DCL, IQGAP1, renal fibrosis

## Abstract

Renal fibrosis, a hallmark of chronic kidney disease (CKD), remains a critical therapeutic challenge with limited effective interventions. Herein, we proposed a multimodal AI‐driven Traditional Chinese Medicine (TCM) symptom prediction model (TCM‐SPred) and predicted potential herb‐symptom associations between herbs and symptoms to obtain agents for treating renal fibrosis. The prediction results of TCM‐SPred revealed that a natural guaianolide sesquiterpene lactone derivative dehydrocostus lactone (DCL, a main chemical constituent of Aucklandiae Radix) demonstrated significant anti‐fibrotic effects in vivo (unilateral ureteral obstruction) and in vitro (TGF‐β1‐induced epithelial‐mesenchymal transition). DCL directly targeted IQGAP1 to inhibit the Wnt signaling pathway by blocking the interaction between IQGAP1 and CCT3. These findings highlight the potential of DCL as a promising therapeutic candidate for renal fibrosis, providing novel insights into the IQGAP1‐CCT3‐Wnt signaling axis as a potential target for renal fibrosis intervention.

## Graphical abstract

Renal fibrosis, a hallmark of CKD, lacks effective treatments. Herein, we developed a multimodal AI model (TCM‐SPred) to identify anti‐fibrotic agents and found that dehydrocostus lactone (DCL) targets IQGAP1 to inhibit Wnt signaling, blocking the interaction between IQGAP1 and CCT3, demonstrating potent anti‐fibrotic activity in vitro and in vivo.

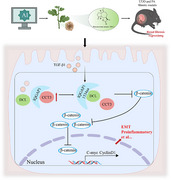

## 1 Introduction

Over the past twenty years, the global mortality rate associated with chronic kidney disease (CKD) has risen significantly, while its prevalence has surged to affect over 840 million individuals worldwide, representing a major global public health burden [1]. Renal fibrosis, the pathological hallmark of progressive CKD, is characterized by excessive extracellular matrix (ECM) deposition and irreversible architectural destruction in renal tissues [2]. This fibrotic cascade is mechanistically linked to epithelial‐mesenchymal transition (EMT), where tubular epithelial cells lose their epithelial characteristics and adopt mesenchymal phenotypes, further driving fibrotic progression [3, 4]. To combat renal fibrosis, a variety of therapeutic options have been investigated, including angiotensin receptor/neprilysin inhibitors (ARNI), sodium‐glucose cotransporter‐2 inhibitors (SGLT2i), and glucagon‐like peptide‐1 (GLP‐1) agonists [5, 6, 7]. However, effective treatments for renal fibrosis are still lacking, often due to insufficient clinical efficacy, limited efficacy in halting fibrosis, and lack of evidence from preclinical or clinical studies. Consequently, developing novel therapeutic agents for renal fibrosis is critically urgent.

The exploration of natural drug candidates from Traditional Chinese Medicine (TCM) with high efficiency and low toxicity to treat complex diseases has become one of the important strategies of drug discovery. There are several natural drug candidates that are reported to possess therapeutic effects on renal fibrosis. For example, Tanshinol B, a characteristic and bioactive constituent of *Salvia miltiorrhiza* Bunge, has emerged as a promising therapeutic candidate for the treatment of renal fibrosis and is administered via intravenous injection at a dose of 40 mg kg^−1^ [8]. Furthermore, other natural products, including puerarin [9], curcumin [10], and artemisinin [11], have also been identified to exhibit moderate biological activities in modulating renal fibrosis, highlighting their potential as therapeutic agents. However, insufficient efficacy and unclear molecular mechanisms have limited their further clinical application.

Recently, artificial intelligence (AI) has been increasingly applied to various phases of drug discovery for its significant time‐saving and efficacy‐enhancing capabilities. In particular, AI‐driven natural product prediction algorithms are considered as a pivotal strategy for discovering novel therapeutic candidates and elucidating their mechanisms for treating human diseases. Till now, these AI‐driven natural product prediction models mainly employ knowledge‐graph embeddings and graph neural networks to anticipate and accelerate the process [12, 13, 14, 15]. For example, in 2023, a literature reported that a network medicine framework was established to reveal the topological relationship between disease symptoms and TCM targets [12]. Additionally, Zhou's research group developed a deep learning‐based target prediction framework HTINet2, that was utilized to predict the targets of TCM [13]. However, these AI‐driven models still need improvement in deeply integrating multimodal data and more fully utilizing sequence and semantic information. Therefore, the development of novel AI‐driven models to effectively discover novel natural products has gained great attention.

**FIGURE 1:**
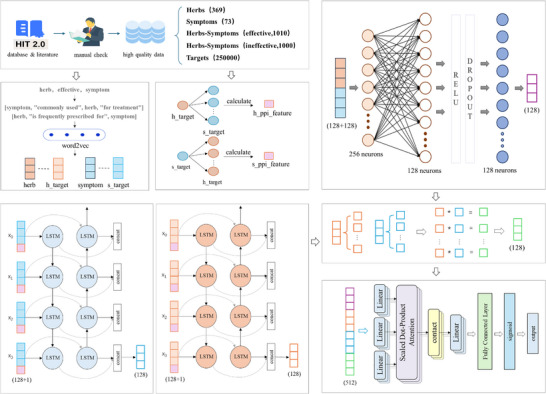
TCM‐SPred Architecture. TCM‐SPred comprises data preprocessing, feature extraction and embedding, and a neural network that integrates a main feature processor, bidirectional target encoders, cross‐modal attention and a classifier.

Herein, a multimodal AI‐driven TCM‐symptom prediction (TCM‐SPred) model that integrated deep neural networks with multi‐source biological feature fusion was proposed (Figure 1). Through conducting semantic embedding of TCM and symptoms via Word2Vec, TCM domain knowledge was represented by vectors. Meanwhile, the correlation between herbal targets and symptom‐related genes was established through the protein‐protein interaction network. Besides, a bidirectional long short‐term memory (LSTM) network integrated with a cross‐modal attention was employed to dynamically capture the potential symptom‐TCM correlation from the fused multimodal representations. Through the prediction of TCM‐SPred, Aucklandiae Radix was identified as a promising therapeutic candidate for renal fibrosis.

Subsequently, dehydrocostus lactone (DCL), the main chemical constituent of Aucklandiae Radix, exhibited a favorable therapeutic effect on unilateral ureteral obstructive (UUO)‐ and folic acid (FA)‐induced renal fibrosis in mice at a therapeutic dose of 1 mg kg^−1^ (i.g.). To elucidate its biological mechanism, a biotin‐tagged DCL chemical probe was designed, and IQGAP1 was considered as a potential target for DCL treatment of renal fibrosis. Then, through a detailed biological study, DCL was further confirmed to ameliorate renal fibrosis by inhibiting the IQGAP1‐CCT3‐Wnt/β‐catenin signaling axis. Collectively, DCL was confirmed as a drug candidate for the treatment of renal fibrosis.

## 2 Results

### 2.1 Discovery of the Potential Anti‐Renal Fibrosis Role of Aucklandiae Radix Based on the TCM‐SPred System

**FIGURE 2:**
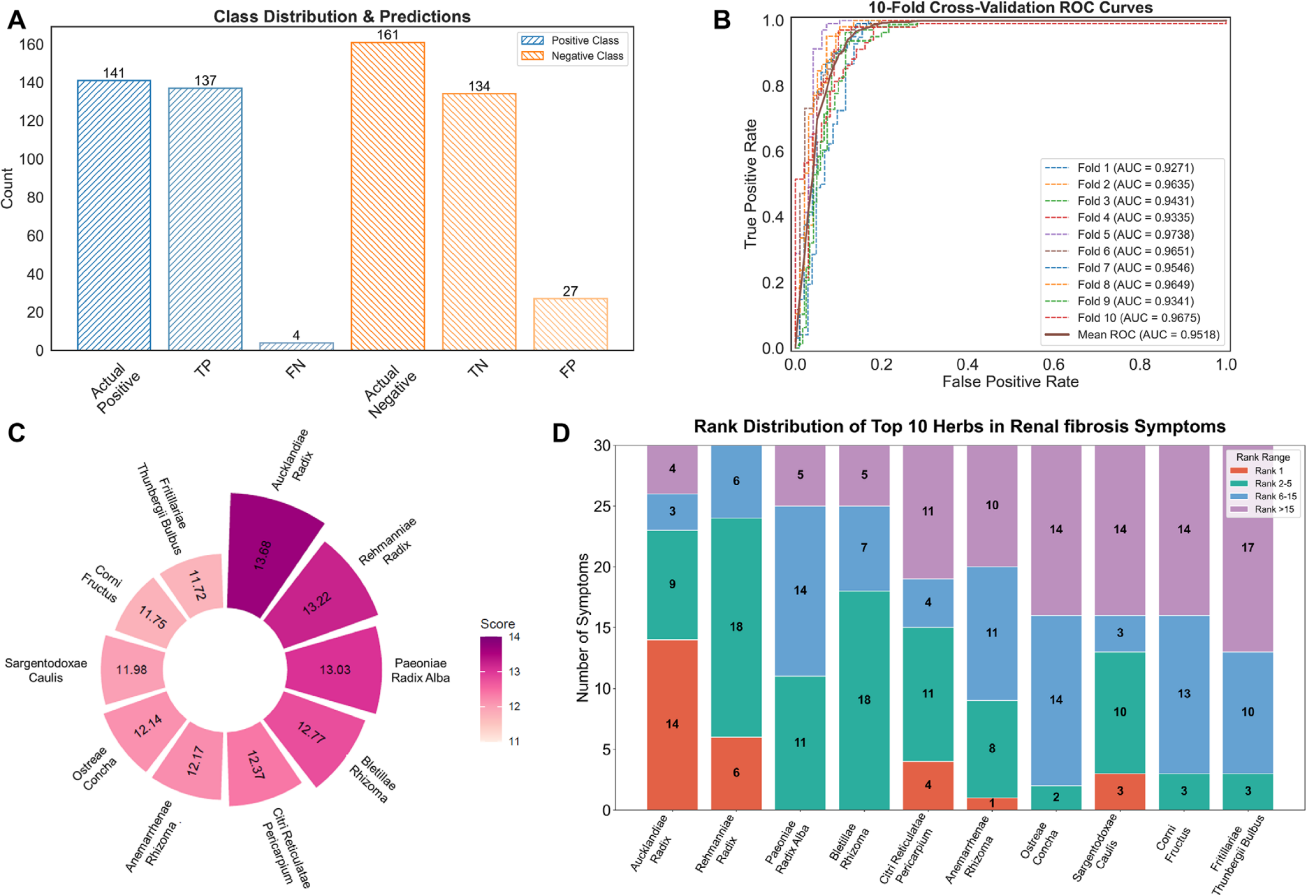
Revealing the Potential Anti‐Renal Fibrosis Activity of Aucklandiae Radix via the TCM‐SPred Prediction System. (A) Class distribution of true labels and model predictions in renal fibrosis symptom dataset. (B) Receiver Operating Characteristic Curve (ROC) of 10‐fold cross‐validation. The dataset is divided into 10 subsets. In each iteration, one subset is selected as the validation set, and the remaining nine subsets are used as the training set. The model is trained and validated during each iteration, and its generalization ability is ultimately evaluated by averaging the results of the 10 validation runs. (C) Top 10 Chinese Herbs for Renal Fibrosis‐Associated Symptoms (Total Score). Predictive scores for all candidate herbs were computed on the basis of 30 Renal fibrosis‐related symptoms; the ten herbs with the highest aggregate scores are presented as the model's prioritized recommendations. (D) Rank Distribution of Top 10 Herbs in Renal Fibrosis‐Associated Symptoms. Each herb's predicted affinity to every one of the 30 Renal fibrosis ‐associated symptoms is individually ranked, and the resulting ranks are aggregated to depict the model's symptom‐wise prioritization across the selected herbs.

The detailed establishment of an AI‐driven TCM‐SPred was described in the methods section, and the performance of TCM‐SPred model was shown in Figure 2. The confusion matrix decomposition results demonstrated that the favorable calculation accuracy was achieved in all disease categories, with a true positive (TP) rate exceeding 97.16% (Figure 2A). Subsequently, we conducted cross‐validation experiments to further assess the robustness and generalizability of TCM‐SPred. As shown in Figure 2B, the AUC values ranged from 0.92 to 0.97, with a mean value of 0.95 ± 0.01, indicating that the model maintained high discriminative performance regardless of the dataset partitioning method. Thus, these comprehensive performance metrics revealed that the proposed TCM‐SPred model exhibited robust convergence and high predictive accuracy.

Considering the excellent performance of TCM‐SPred model, it was employed to predict potential therapeutic agents for renal fibrosis. To facilitate this prediction, a set of kidney disease symptoms was retrieved from the SymMap database (v2.0) [16], resulting in 127 clinical symptoms. Then, according to the standardized methodology for TCM symptom description, the diverse 127 clinical symptoms were mapped to 30 high‐confidence renal fibrosis‐associated symptoms, and these 30 renal fibrosis‐associated symptoms were listed in Table S1. Through the prediction of TCM‐SPred, Aucklandiae Radix was predicted to possess a favorable herb‐symptom correlation score (score = 13.68, Figure 2C).

Then, we further analyzed the relationship between drug candidates and the symptoms of renal fibrosis (Figure 2D). Detailed analysis revealed that Aucklandiae Radix was predicted to be the preferred therapeutic drug for treating 15 symptoms of renal fibrosis, and it was also employed as a secondary therapeutic agent for treating 12 symptoms of renal fibrosis. Consequently, the prediction of TCM‐SPred demonstrated that Aucklandiae Radix exhibited excellent calculation performance, with a favorable correlation score between TCM herbs and symptoms, suggesting potential therapeutic effects on renal fibrosis.

### 2.2 Aucklandiae Radix Extract Alleviates UUO‐Induced Renal Fibrosis in Mice

To investigate the positive effect of Aucklandiae Radix on renal fibrosis, we initially employed an activity‐guided isolation approach to refine the fractions derived from the crude extraction (Figure S1A). UUO mice were treated with the Aucklandiae Radix extract (ARE) by daily oral gavage for 12 days (Figure S1B). In response to UUO, ARE significantly improved morphology and reduced collagen deposition (Figure S1C). RT‐qPCR analysis showed that ARE dramatically inhibited UUO‐induced gene expression of renal injury markers (*Havcr1, Ngal* and *Klotho*), fibrogenic factors (*Acta2, Fn1, Col1a1, Col3a1, Vim*, and *Tgfb*), inflammatory cytokines (*Il1b* and *Il18*), EMT and adhesion molecules (*Cdh1, Snail1, Snail2* and *Icam*) (Figure S1D–G). Further western blot analysis confirmed that E‐Cadherin, a marker of epithelium cells, was upregulated by ARE in the kidneys of UUO mice. The mesenchymal cell and fibrotic markers, such as Vimentin (VIM), SNAI1, and α‐smooth muscle actin (α‐SMA) were significantly downregulated by ARE (Figure S1H). These results confirmed the remarkable therapeutic potential of Aucklandiae Radix in mitigating renal fibrosis.

### 2.3 DCL Exerts Anti‐Renal Fibrotic Effects in TGF‐β1‐Induced Renal Tubular Epithelial Cells

The characteristic high‐abundance bioactive components, including artemisinin [17] in Artemisia annua and andrographolide [18] in Andrographis paniculata, are typically served as primary pharmacological effectors in TCMs. The main components in Aucklandiae Radix are two sesquiterpene lactones: DCL and costunolide (CT) (Figure S2A). We evaluated the anti‐renal fibrosis activity of these two sesquiterpene lactones in TGF‐β1‐stimulated HK‐2 cells to further identify the bioactive constituents in Aucklandiae Radix. Notably, DCL significantly suppressed TGF‐β1‐induced upregulation of *ACTA2*, *HAVCR1*, and *IL1B* at the mRNA level, whereas CT exhibited no discernible activity (Figure S2B). These findings indicated that DCL served as the primary anti‐renal fibrosis constituent in Aucklandiae Radix.

**FIGURE 3:**
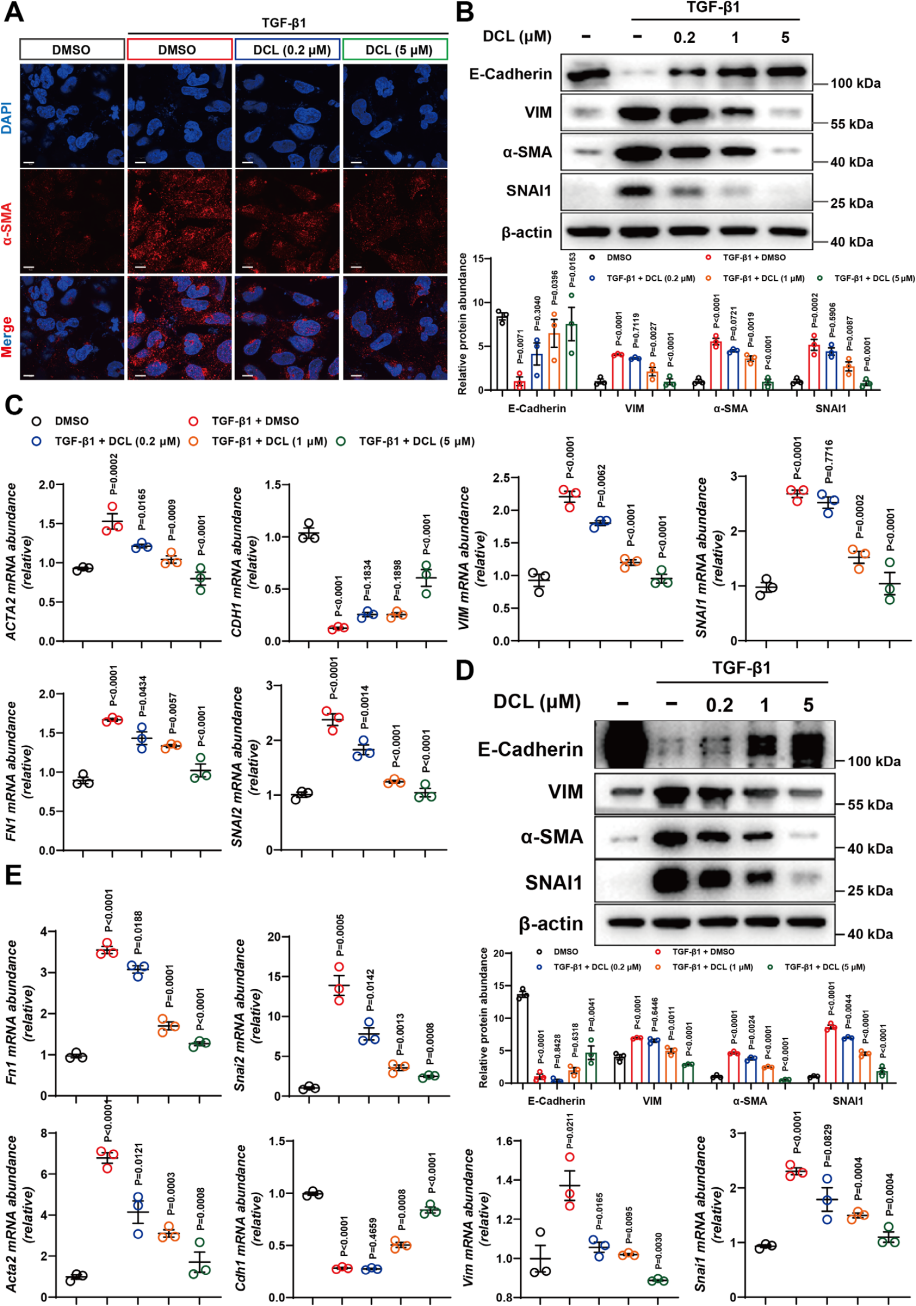
DCL Alleviates TGF‐β1‐Induced Fibrosis in Renal Tubular Epithelial Cells. (A) Representative fluorescent photographs of immunofluorescence staining for α‐SMA (red) in HK‐2 cells in indicated conditions. Nuclei were counterstained with DAPI (blue). Scale bar, 10 µm. (B) Representative western blot for E‐Cadherin, VIM, α‐SMA and SNAI1 in TGF‐β1‐stimulated HK‐2 cells treated with indicated concentrations of DCL for 24 h (n = 3 per group). (C) Effects of DCL on the expression profiles of fibrosis‐, injury‐, EMT‐, and inflammation‐related genes in HK‐2 cells stimulated with TGF‐β1 (10 ng mL^−1^). qRT‐PCR assay was carried out in cells treated with indicated concentrations of DCL for 24 h (n = 3 per group). (D) Representative western blot for E‐Cadherin, VIM, α‐SMA, and SNAI1 in TGF‐β1‐stimulated TCMK‐1 cells treated with indicated concentrations of DCL for 24 h (n = 3 per group). (E) Effects of DCL on the expression profiles of fibrosis‐, injury‐, EMT‐, and inflammation‐related genes in TCMK‐1 cells stimulated with TGF‐β1 (10 ng mL^−1^). qRT‐PCR assay was carried out in cells treated with indicated concentrations of DCL for 24 h (n = 3 per group). Data were presented as mean ± SEM and statistical differences were determined by one‐way ANOVA.

To further validate the anti‐fibrotic activity of DCL in vitro, HK‐2 cells were stimulated with TGF‐β1 and treated with DCL (0.2, 1, and 5 µM). Immunofluorescence staining revealed that DCL inhibited the upregulation of the fibrotic marker α‐SMA induced by TGF‐β1 (Figure 3A). Western blotting and qRT‐PCR results demonstrated that the levels of intracellular fibrosis‐, injury‐, EMT‐, and inflammation‐related markers were reversed by the administration of DCL (Figure 3B,C, Figure S2C,D). Additionally, DCL also exhibited similar therapeutic effect on TGF‐β1‐stimulated mouse renal tubular epithelial TCMK‐1 cells (Figure 3D,E, Figure S2E,F).

Besides, we examined the effects of DCL on primary mouse bone marrow‐derived macrophages (BMDMs) and fibroblasts under TGF‐β1 stimulation, because fibroblasts and BMDMs play a significant role in the progression of renal fibrosis. Results showed that DCL only modestly reduced the extent of fibrosis triggered by TGF‐β1 in fibroblasts (Figure S2 G–I). DCL failed to alleviate TGF‐β1‐induced macrophage‐to‐fibroblast transition (MMT, Figure S2 J–L). Consequently, proximal tubular epithelial cells might be the primary target of DCL.

### 2.4 DCL Inhibited UUO‐induced Renal Fibrosis in Mice

**FIGURE 4:**
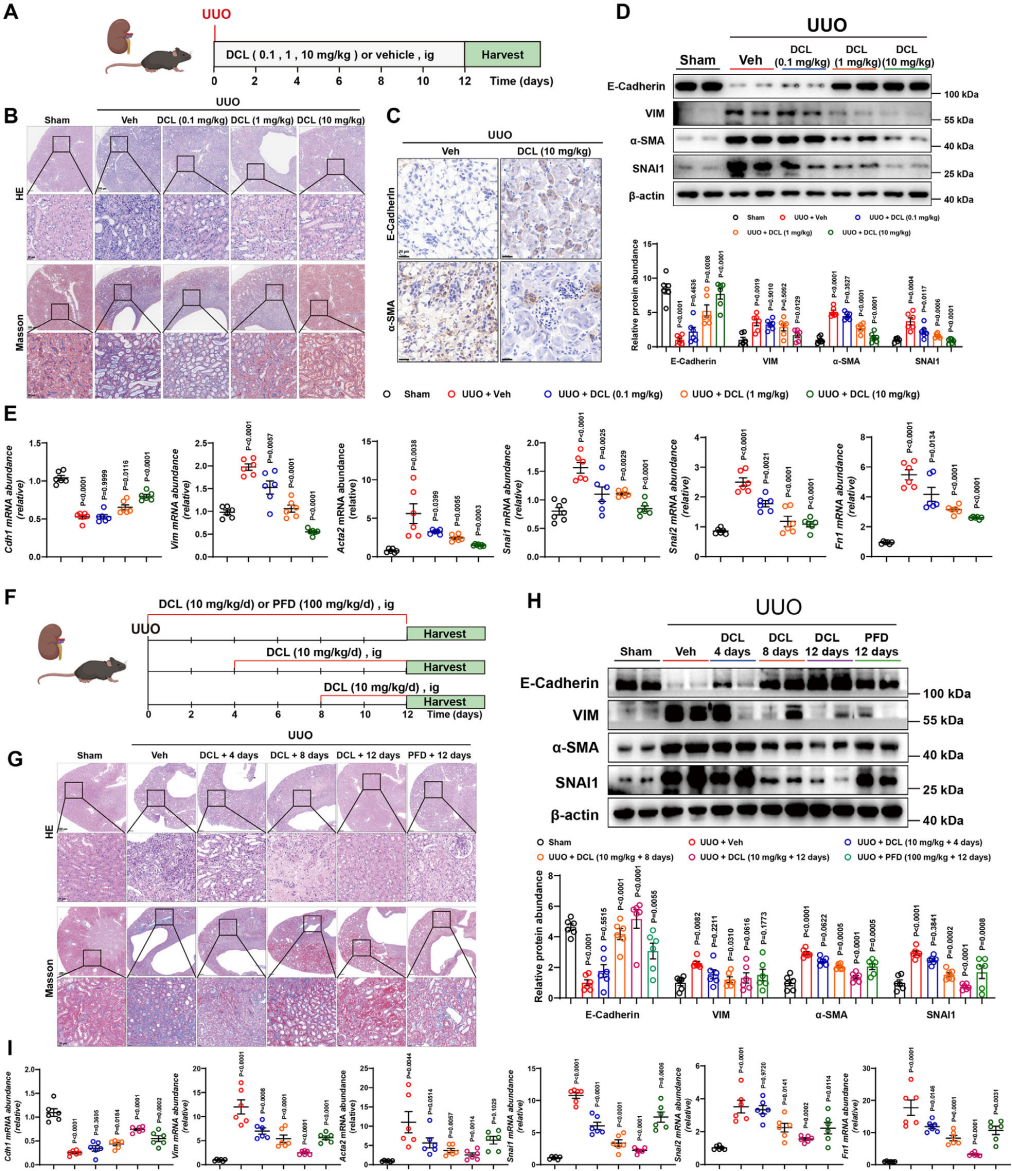
DCL Alleviates Renal Fibrosis in vivo. (A) Schematic overview of the experimental design of the UUO‐induced fibrosis model in mice. Sham or UUO surgery was performed on C57BL/6J mice. Vehicle (Veh) or DCL (0.1, 1 and 10 mg kg^−1^) was intragastric administered to mice daily for the consecutive 14 days after the surgery. (B) Representative images of mouse renal tissues stained with HE and Masson in indicated groups. Scale bar, 20 µm. (C) Representative images of mouse renal tissues stained with α‐SMA and E‐Cadherin indicated groups. Scale bar, 20 µm. (D) Kidney homogenate samples were analyzed by western blotting to quantify the protein levels of E‐Cadherin, VIM, α‐SMA and SNAI1 (n = 6 per group). (E) Kidney homogenate samples were analyzed by qRT‐PCR to quantify the gene levels of EMT marker and fibrogenic factors (n = 6 per group). (F) Schematic overview of the experimental design of the UUO‐induced fibrosis model in mice. Sham or UUO surgery was performed on C57BL/6J mice. Veh, DCL (10 mg kg^−1^), or PFD (100 mg kg^−1^) Mice were administered intragastrically daily on days 0, 4, and 8 after surgery, ending on days 12. (G) Representative images of mouse renal tissues stained with HE and Masson in the indicated groups. Scale bar, 20 µm. (H) Kidney homogenate samples were analyzed by western blotting to quantify the protein levels of E‐Cadherin, VIM, α‐SMA, and SNAI1 (n = 6 per group). (I) Kidney homogenate samples were analyzed by qRT‐PCR to quantify the gene levels of EMT marker and fibrogenic factors (n = 6 per group). Data were presented as mean ± SEM, and statistical differences were determined by one‐way ANOVA.

Subsequently, we investigated whether DCL serves as the material basis for the anti‐renal fibrosis effect of Aucklandiae Radix (Figure 4A). HE and Masson staining showed that UUO‐induced renal atrophy, renal tubular expansion, and collagen deposition in the renal cortex and medulla were observed (Figure 4B). Immunohistochemistry indicated that DCL treatment increased the E‐Cadherin expression and reduced α‐SMA expression in the kidneys of UUO mice (Figure 4C). RT‐qPCR and western blot analyses showed that UUO‐stimulated EMT progression, pro‐inflammatory cytokines, kidney injury, and fibrogenic factors were attenuated by DCL (Figure 4D,E, Figure S3A).

Additionally, therapeutic administration of DCL for 4 or 8 days could significantly alleviate renal fibrosis and EMT progression, demonstrating a more favorable effect than the positive control drug pirfenidone (Figure 4F–I, Figure S3B). Meanwhile, in another acute renal fibrosis model induced by FA, the administration of DCL could also significantly alleviate renal injury and fibrosis (Figure S4A–E).

### 2.5 IQGAP1 was a Direct Target of DCL in Tubular Epithelial Cells

**FIGURE 5:**
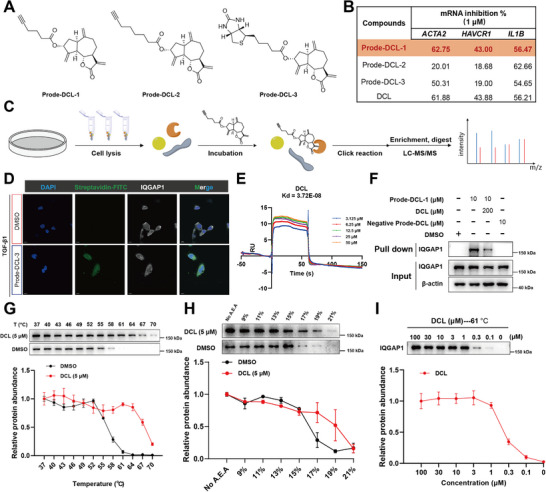
DCL Directly Targets IQGAP1. (A) The structures of three affinity chemical probes. (B) Inhibition of *ACTA2*, *HAVCR1*, and *IL1β* gene expression by different probe compounds (1 µM, n = 3 per group). (C) Schematic illustration of target identification. Labeling experiments were performed in HK‐2 cell lysate, using DMSO as the control (CON). The resulting samples were subjected to a click chemistry reaction for streptavidin bead enrichment. (D) Co‐localization assay of IQGAP1 and Prode‐DCL‐3 in HK‐2 cells. Cells were treated with vehicle or 10 µM Prode‐DCL‐3 for 3 h and then stained with anti‐IQGAP1 antibody (white) and Streptavidin‐FITC (green). Scale bar, 10 µm. (E) SPR analysis of interactions between DCL and IQGAP1. (F) Western blot analysis on endogenous IQGAP1 protein after Prode‐DCL‐1 pulldown in HK‐2 cells. (G) CETSA analysis of intracellular binding between DCL and IQGAP1. Protein levels were investigated at different temperatures under the treatment of DCL (5 µM) in HK‐2 cells for 3 h (n = 3 per group). (H) SIP analysis of intracellular binding between DCL and IQGAP1. Protein levels were investigated at different organic solvent under the treatment of DCL (5 µM) in HK‐2 cells (n = 3 per group). (I) Protein levels were investigated at 61°C under the indicated concentrations of DCL in HK‐2 cells (n = 3 per group).

To identify the functional targets of DCL that were responsible for its potent anti‐fibrotic effect, several chemical probes for affinity purification were prepared. The cellular potencies of chemical probes (Probe‐DCL‐1, 2, and 3) were evaluated in vitro, and Probe‐DCL‐1 showed the most inhibitory effects on gene expression of *ACTA2, HAVCR1* and *IL1B* (Figure 5A,B) and protein expression of E‐Cadherin, VIM, α‐SMA, and SNAI1 (Figure S5A). Then, we conducted a pulldown assay by incubating the HK‐2 cell lysates with Probe‐DCL‐1 or DMSO. The proteins binding to DCL were precipitated by streptavidin‐agarose beads, followed by SDS‐PAGE and mass spectrometry analysis (Figure 5C). Utilizing MaxQuant software for bioinformatics analysis, a total of 334 proteins were identified as potential binding targets for DCL (Table S2). Among the top interacting proteins in protein abundances, IQGAP1 [19, 20], CAND1 [21], NAMPT [22], SF3B3 [23], and EPRS [24] played potential roles in the pathogenesis of fibrosis. We confirmed the binding between the top interacting proteins and DCL, cellular thermal shift assay (CETSA) and pull‐down experiments indicated that IQGAP1 exhibited the strongest interaction with DCL (Figure S5B–D).

To further investigate the interaction between DCL and IQGAP1, we employed a series of biophysical analyses. Fluorescence imaging experiments showed that Probe‐DCL‐3 was co‐localized with IQGAP1 (Figure 5D). Surface plasmon resonance (SPR) analysis showed that DCL had strong binding affinity for IQGAP1 (*K*_d_ = 37.2 nM, Figure 5E). Probe‐DCL‐1 effectively pulled IQGAP1 protein out, while the addition of unlabeled DCL reduced their binding due to competitive binding (Figure 5F). The negative probe synthesized by reducing the carbon‐carbon double bond of the Michael receptor could not pull out IQGAP1 protein (Figure 5F, Figure S5E). CETSA assay revealed that DCL enhanced the thermal stability of IQGAP1 protein (Figure 5G). The solvent‐induced protein precipitation (SIP) assay also demonstrated that DCL could directly bind to IQGAP1, evidenced by IQGAP1 degradation at a higher proportion of organic solvent (A.E.A.) (Figure 5H). Additionally, DCL could dose‐dependently preserve the stability of IQGAP1 at 61°C (Figure 5I). Collectively, these results suggested that DCL directly bound to IQGAP1.

### 2.6 IQGAP1 Mediated TGF‐β1‐Induced Changes in Tubular Epithelial Cell

**FIGURE 6:**
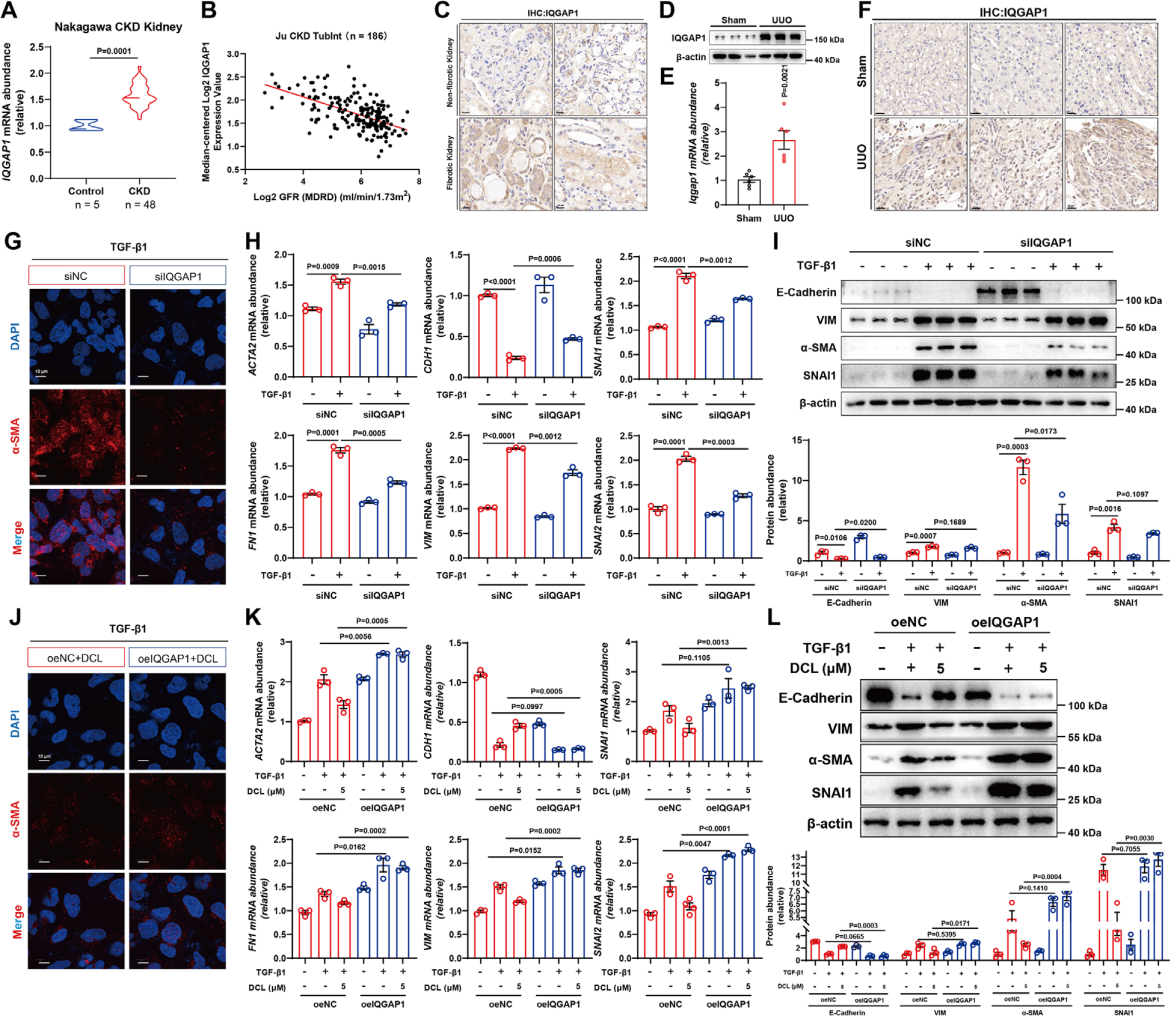
DCL is Associated with the Progression of Renal Fibrosis. (A) mRNA expression levels of *IQGAP1*. Data analysis from Nephroseq database (‘Ju CKD Tublnt’ dataset, median‐centered log_2_). n = 5 samples in control group, n = 45 samples in CKD group. Unpaired *t*‐test. ns, no significant difference. (B) Pearson's correlation of IQGAP1 with estimated glomerular filtration rate (eGFR). (C) Tissue adjacent sections of kidney from patients with non‐fibrotic kidney or fibrotic kidney by immunohistochemistry. Scale bar = 20 µm. n = 2 samples per group. (D) the protein expression levels of IQGAP1 in mice renal tissues (n = 3 per group). (E) The mRNA levels of *IQGAP1* in mice renal tissues (n = 6 per group). (F) Representative photographs of immunohistochemistry for IQGAP1 in mice renal tissues. Scale bar, 20 µm. (G) Representative fluorescent photographs of immunofluorescence staining for α‐SMA (red) in HK‐2 cells 3D spheroids in indicated conditions. Nuclei were counterstained with DAPI (blue). Scale bar, 10 µm. (H) HK‐2 cells samples were analyzed by qRT‐PCR to quantify the gene levels of EMT marker and fibrogenic factors (n = 3 per group). (I) Western blot (top panel) and quantification (under panel) of the protein expression of E‐Cadherin, VIM, α‐SMA, and SNAI1 in HK‐2 cells. β‐actin served as loading control (n = 3 per group). (J) Representative fluorescent photographs of immunofluorescence staining for α‐SMA (red) in HK‐2 cells 3D spheroids in indicated conditions. Nuclei were counterstained with DAPI (blue). Scale bar, 10 µm. (K) HK‐2 cells samples were analyzed by qRT‐PCR to quantify the gene levels of EMT marker and fibrogenic factors (n = 3 per group). (L) Western blot (top panel) and quantification (under panel) of the protein expression of E‐Cadherin, VIM, α‐SMA, and SNAI1 in HK‐2 cells. β‐actin served as loading control (n = 3 per group). Data were presented as mean ± SEM and statistical differences were determined by one‐way ANOVA.

We examined the expression of the *Iqgap1* by analyzing the renal transcriptomics database Nephroseq (https://www.nephroseq.org/resource/login.html). In the Nakagawa CKD kidney dataset, the mRNA of *IQGAP1* was significantly upregulated in kidney biopsy tissues of the CKD patients (n = 48) compared with controls (n = 5) (Figure 6A). The levels of IQGAP1 showed a strong negative correlation with the estimated glomerular filtration rate in the Ju CKD Tublnt dataset (Figure 6B). We analyzed microscopically dissected human kidney samples collected from patients with renal fibrosis and non‐renal fibrosis. As shown in Figure 6C and Table S3, immunohistochemistry revealed that IQGAP1 protein level in patients with renal fibrosis were significantly higher than that in patients without renal fibrosis. Subsequently, we determined the levels of IQGAP1 protein in mice and HK‐2 cells. The results of immunohistochemistry, RT‐qPCR, and western blots showed that the mRNA and protein levels of IQGAP1 were significantly increased in the kidneys of UUO‐induced mice (Figure 6D–F). In addition, under TGF‐β1 stimulation, the expression levels of IQGAP1 in both mRNA and protein increased in HK‐2 cells (Figure S8E,F).

To investigate the role of IQGAP1 in kidney fibrosis, we knocked down or overexpressed IQGAP1 in HK‐2 cells (Figure S6A–E). Immunofluorescence staining results showed that in the presence of TGF‐β1, α‐SMA was downregulated in IQGAP1‐knockdown HK‐2 cells (Figure 6G). Knockdown of IQGAP1 significantly downregulated TGF‐β1‐induced mesenchymal markers VIM, α‐SMA, and SNAI1, while upregulating epithelial marker E‐Cadherin (Figure 6I). RT‐qPCR analysis revealed that TGF‐β1‐stimulated EMT progression, pro‐inflammatory cytokines, kidney injury, and fibrogenic factors expression were improved by IQGAP1 knockdown (Figure 6H, Figure S6F,G). Conversely, overexpression of IQGAP1 promoted transcription and protein expression of EMT‐related genes, and blocked the inhibitory effects of DCL on TGF‐β1‐induced HK‐2 cells fibrosis (Figure 6J–L, Figure S6H,I).

### 2.7 DCL Bound to IQGAP1 Cys1534

**FIGURE 7:**
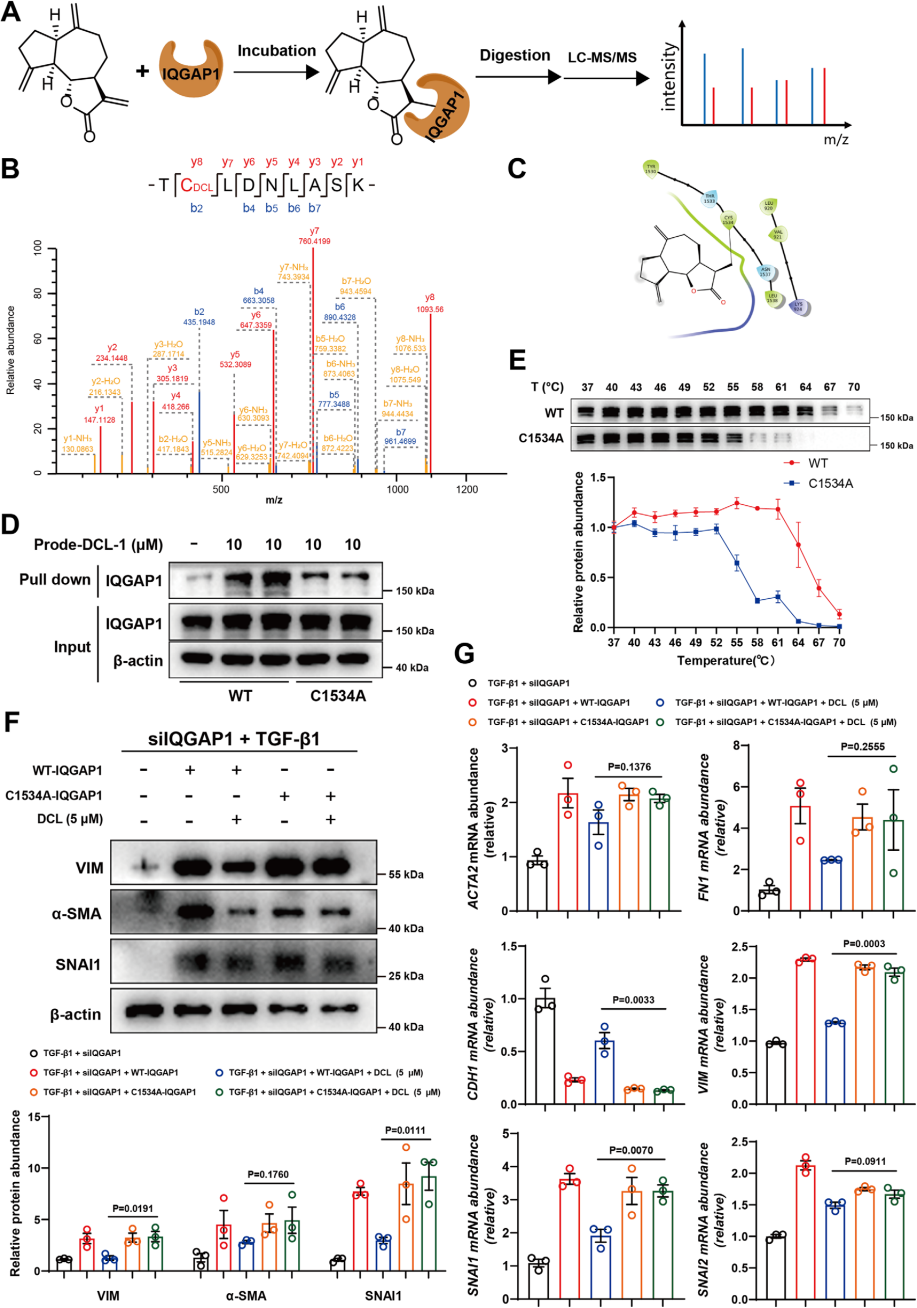
Mapping the Binding Sites of DCL on IQGAP1. (A) Schematic illustration of binding site mapping between DCL and IQGAP1. DCL was incubated with purified IQGAP1 and was then successively subjected to trypsin digestion and MS/MS. (B) LC‐MS/MS plot after the incubation of DCL with IQGAP1. (C) Molecular docking result. (D) Pull‐down experiments of Prode‐DCL‐1 on different IQGAP1 mutants in HEK293T cells. C1534A: Cys1534 mutates into Ala. (E) CETSA experiments of DCL on different IQGAP1 mutants in HEK293T cells. C1534A: Cys1534 mutates into Ala (n = 3 per group). (F) Western blot experiments of DCL on different IQGAP1 mutants in HK‐2 cells. C1534A: Cys1534 mutates into Ala (n = 3 per group). (G) qRT‐PCR experiments of DCL on different IQGAP1 mutants in HEK293T cells. C1534A: Cys1534 mutates into Ala (n = 3 per group). Data were presented as mean ± SEM, and statistical differences were determined by one‐way ANOVA.

To elucidate the binding mode of DCL with IQGAP1, we investigated the DCL‐IQGAP1 complex using liquid chromatography‐tandem mass spectrometry (LC‐MS/MS) (Figure 7A). As shown in Figure 7B, the results revealed that the molecular weight of peptide 1533‐TCLDNLASK‐1541 increased by 230.13, corresponding to the photoactivated DCL. Besides, through the utilization of Schrödinger Maestro 2019, the covalent docking study also confirmed the binding interaction of DCL with Cys1534 in IQGAP1 (Figure 7C). To verify the binding of DCL to Cys1534, an alanine residue was introduced by site‐directed mutagenesis to replace Cys1534. The results revealed that mutagenesis with an alanine residue reduced the thermal stability of DCL on IQGAP1 protein, and compared with wild‐type IQGAP1, it also reduced the expression of IQGAP1 protein pulled out by DCL (Figure 7D,E). In addition, compared with wild‐type IQGAP1, the inhibitory effect of DCL on TGF‐β1‐stimulated EMT progression and fibrosis was diminished by Cys1534 mutation (Figure 7F,G, Figure S7A,B). Collectively, these finding suggested that DCL covalently bound with Cys1534 in IQGAP1.

### 2.8 IQGAP1 Inhibited Wnt Signaling Pathway

**FIGURE 8:**
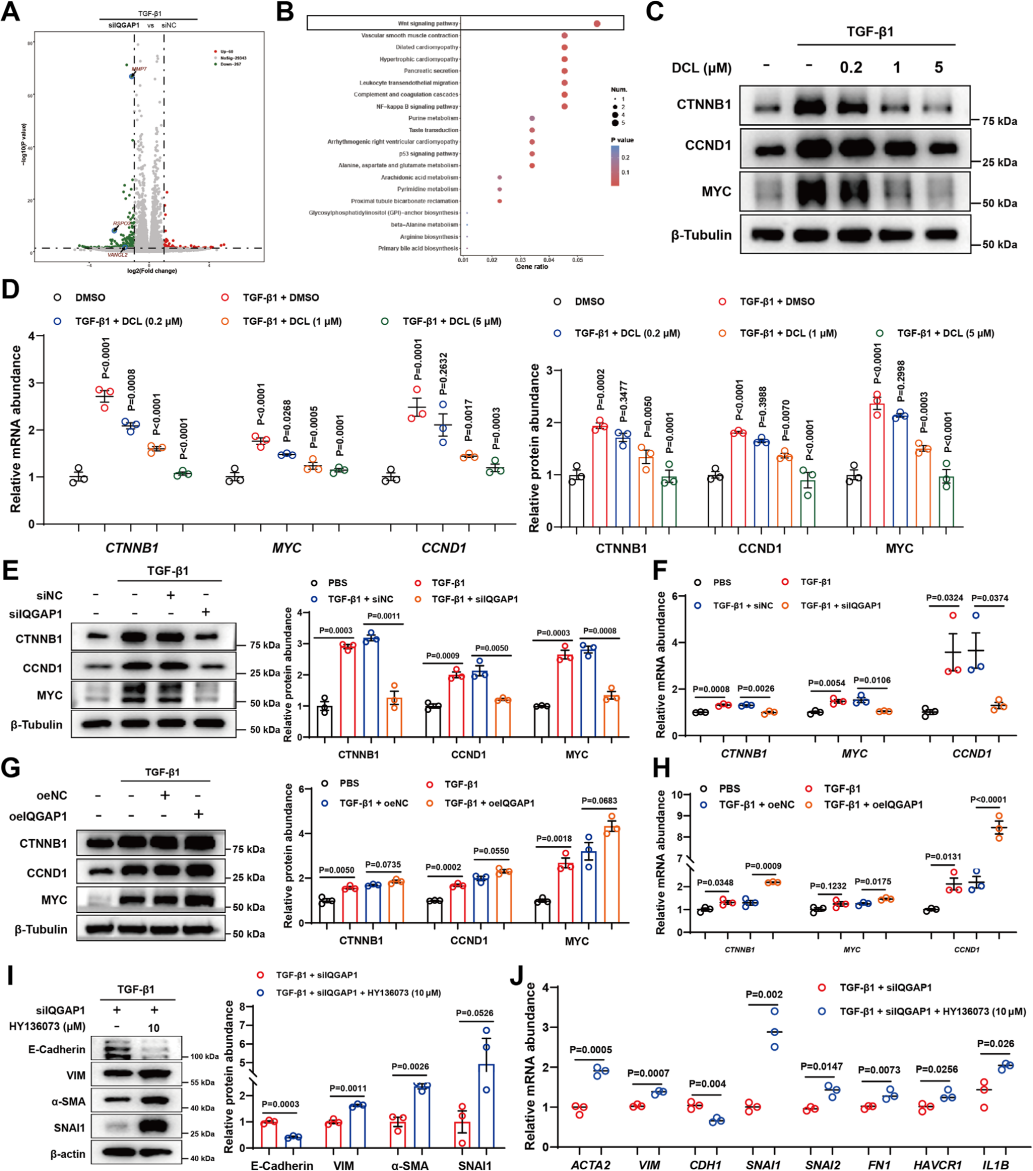
IQGAP1 Inhibits the Wnt Signaling Pathway. (A) Volcano plots depicting the differentially expressed genes between siNC‐treated and siIQGAP1‐treated groups. (B) Barplot showing all the down‐regulated KEGG pathways termed signaling pathway. (C,D) Western blot (above) and RT‐qPCR (below) analysis of CTNNB1, CCND1 and MYC expression in HK‐2 cells treated with TGF‐β1 (10 ng/mL, n = 3 per group). (E,F) Western blot(left) and RT‐qPCR(right) analysis of CTNNB1, CCND1, and MYC expression in IQGAP1 knockdown HK‐2 cells (n = 3 per group). (G,H) Western blot (left) and RT‐qPCR (right) analysis of CTNNB1, CCND1, and MYC expression in IQGAP1‐overexpressed HK‐2 cells (n = 3 per group). (I,J) Western blot (left) and RT‐qPCR (right) analysis of EMT progression and fibrosis in HK‐2 cells treated with Wnt signaling pathway activator HY136073 and TGF‐β1 (10 ng mL^−1^) treated with IQGAP1 knockdown (n = 3 per group). Data were presented as mean ± SEM, and statistical differences were determined by one‐way ANOVA.

To investigate the molecular mechanism underlying the anti‐fibrosis effect of IQGAP1, the transcriptomes of IQGAP1‐knockdown HK‐2 cells were analyzed by KEGG analysis. Subsequently, we analyzed the data in transcriptomes of cells and identified the Wnt signaling pathway as the most significantly changed pathway (Figure 8A,B). To further verify the regulatory effects of DCL and IQGAP1 on the Wnt signaling pathway, western blots and RT‐qPCR were used to detect the activation of Wnt signaling in vitro and in vivo. After TGF‐β1 stimulation, the Wnt signaling pathway was activated in HK‐2 cells; moreover, DCL treatment and IQGAP1 knockdown both suppressed Wnt signaling pathway (Figure 8C–F). Conversely, IQGAP1 overexpression promoted Wnt‐associated genes transcription and protein expression in TGF‐β1‐induced HK‐2 cells (Figure 8G,H). To further explore whether the function of IQGAP1 in HK‐2 cells EMT depends on the Wnt signaling pathway, we treated the IQGAP1‐knocked down HK‐2 cells with Wnt signaling agonist (Wnt pathway activator 2: HY136073), the agonist treatment diminished the improvement of IQGAP1 knockdown on EMT progression and fibrosis in response to TGF‐β1 in HK2 cells (Figure 8I,J). In conclusion, these findings demonstrated that DCL could suppress Wnt signaling pathway under conditions of renal fibrosis by binding to IQGAP1.

### 2.9 DCL Blocked the Interaction Between IQGAP1 and CCT3 to Inactivating Wnt Signaling Pathway

**FIGURE 9:**
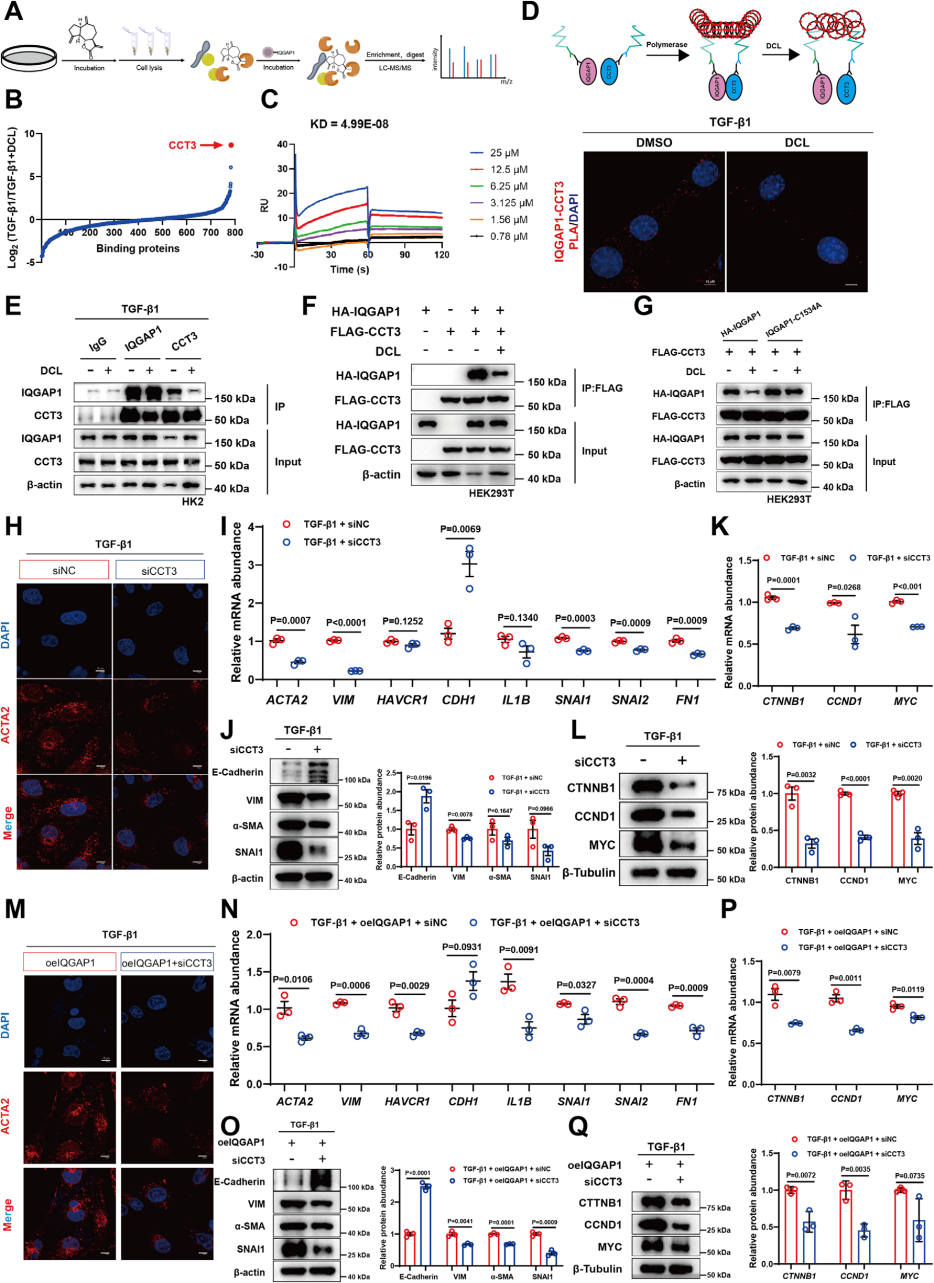
DCL Blocked the Interaction between IQGAP1 and CCT3, Inhibit the Aberrant Activation of Wnt Signaling Pathway, and Improved the Progression of Renal Fibrosis. (A) LC‐MS/MS analysis of DCL‐treated HK‐2 cell lysates by co‐immunoprecipitation. Co‐immunoprecipitation was subjected to trypsinization followed by MS/MS. (B) Binding protein data graph (TGF‐β1 versus TGF‐β1 + DCL (5 µM) in each experiment). (C) SPR analysis of interactions between IQGAP1 and CCT3. (D) Schematic diagram of in situ proximity ligation assay (IQGAP1‐CCT3 PLA). The interaction between IQGAP1 and CCT3 (IQGAP1‐CCT3, red arrow) in HK‐2 cells was analyzed by PLA, and DCL inhibited the interaction between the two. Scale bars, 10 µm. (E) Co‐immunoprecipitation of IQGAP1 and CCT3 in HK‐2 cells stimulated with TGF‐β1 (10 ng mL^−1^) for 24 h. (F) Co‐immunoprecipitation of IQGAP1 and CCT3 in HEK293T cells stimulated with TGF‐β1 (10 ng mL^−1^) for 24 h. G, Coimmunoprecipitation of IQGAP1 and CCT3 in HEK293T cells transfected with IQGAP1‐C1534A plasmid. (H) Representative fluorescent photographs of immunofluorescence staining for α‐SMA (red) in HK‐2 cells 3D spheroids with CCT3 knockdown in indicated conditions. Nuclei were counterstained with DAPI (blue). (I) HK‐2 cells samples were analyzed by qRT‐PCR to quantify the gene levels of EMT marker and fibrogenic factors (n = 3 per group). (J) Western blot (above) and quantification (below) of the protein expression of E‐Cadherin, VIM, α‐SMA, and SNAI1 in HK‐2 cells. β‐actin served as loading control (n = 3 per group). (K,L) Western blot (below) and RT‐qPCR (above) analysis of CTNNB1, CCND1, and MYC expression in CCT3 knockdown HK‐2 cells (n = 3 per group). (M) Representative fluorescent photographs of immunofluorescence staining for α‐SMA (red) in HK‐2 cells 3D spheroids with CCT3 knockdown based on IQGAP1 overexpression in indicated conditions. Nuclei were counterstained with DAPI (blue). N, HK‐2 cells samples were analyzed by qRT‐PCR to quantify the gene levels of EMT marker and fibrosis factors (n = 3 per group). (O) Western blot (left) and quantification (right) of the protein expression of E‐Cadherin, VIM, α‐SMA and SNAI1 in HK‐2 cells. β‐actin served as loading control (n = 3 per group). (P,Q) Western blot (below) and RT‐qPCR (above) analysis of CTNNB1, CCND1, and MYC expression in HK‐2 cells with IQGAP1 overexpression and knockdown of CCT3 (n = 3 per group). Data were presented as mean ± SEM and statistical differences were determined by one‐way ANOVA.

Since DCL did not affect the protein expression of IQGAP1 both in vivo and in vitro (Figure S8A–F), we further investigated the mechanism by which DCL regulated the Wnt signaling pathway through IQGAP1. To identify potential IQGAP1‐interacting proteins under DCL treatment, the protein in HK‐2 cells was co‐immunoprecipitated with the anti‐IQGAP1 antibody, and the immunoprecipitates were analyzed by LC‐MS/MS (Figure 9A). Among the identified proteins, CCT3 had been reported to regulate the activity of Wnt signaling pathway (Figure 9B) [25, 26]. SPR results showed that IQGAP1 displayed a favorable binding affinity with CCT3 (Figure 9C). In situ proximity ligation assay (PLA) was performed to validate the interaction of IQGAP1 and CCT3, and PLA results revealed strong fluorescent signals in HK‐2 cells (Figure 9D), suggesting a direct interaction between IQGAP1 and CCT3. We further confirmed that IQGAP1 bound to CCT3 in HK‐2 cells by co‐immunoprecipitation, but this interaction was blocked by DCL treatment (Figure 9E,F). Moreover, when the Cys1534 of IQGAP1 was mutated, DCL could not affect the interaction between IQGAP1 and CCT3 (Figure 9G).

We next determined the effect of CCT3 on fibrosis in vitro (Figure 9H–J, Figure S9A,B). CCT3 knockdown by siRNA in HK‐2 cells suppressed the EMT progression and fibrosis in response to TGF‐β1 and IQGAP1 overexpression (Figure 9M–O). Additionally, CCT3 knockdown significantly inhibited the Wnt signaling pathway activated by TGF‐β1 (Figure 9K,L). Conversely, CCT3 overexpression promoted TGF‐β1‐induced pathway under the conditions of DCL treatment or IQGAP1 overexpression (Figure 9P,Q, Figure S9C,D). Collectively, these results indicated that DCL blocked the interaction between IQGAP1 and CCT3 to inactivate Wnt signaling pathway.

### 2.10 Tubular Epithelial Cells‐specific IQGAP1 or CCT3 Deletion Inhibited Renal Fibrosis in Mice

**FIGURE 10:**
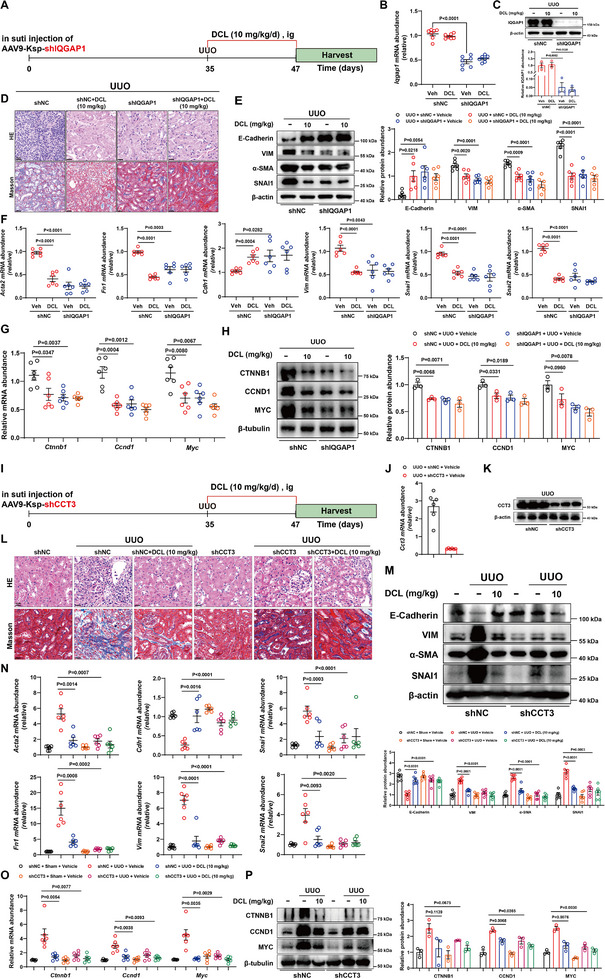
Specific IQGAP1 or CCT3 Deletion Inhibited Renal Fibrosis in Mice. (A) Schematic diagram of IQGAP1 knockdown in mice. Mice were in situ injected with AAV9 encoding IQGAP1 or scramble. After the injection for 5 weeks, the mice were subjected to UUO surgery. Veh or DCL (10 mg kg^−1^) was intragastrically administered to mice daily for the consecutive 12 days after the surgery. (B) mRNA levels of *Iqgap1* in the cortices of kidneys (n = 6 per group). (C) IQGAP1 protein levels in the kidneys of AAV9‐NC and AAV9‐IQGAP1 mice (n = 3 per group). (D) Representative images of mouse tissues stained with HE and Masson in the indicated groups. Scale bar, 50 µm. (E) Representative western blot for fibrotic and EMT markers in indicated groups (n = 6 per group). (F) mRNA levels of fibrotic and EMT genes in indicated groups (n = 6 per group). Gene expression levels were normalized to *Gapdh*. (G) mRNA levels of *Ctnnb1*, *Ccnd1*, and *Myc* genes in indicated groups (n = 6 per group). Gene expression levels were normalized to *Gapdh*. (H) Representative western blot for CTNNB1, CCND1, and MYC markers in groups (n = 3 per group). (I) Schematic diagram of CCT3 knockdown in mice. Mice were in situ injected with AAV9 encoding CCT3 or scramble. After the injection for 5 weeks, the mice were subjected to UUO surgery. Veh or DCL (10 mg/kg) was intragastrically administered to mice daily for the consecutive 12 days after the surgery. (J) mRNA levels of *Cct3* in the cortices of kidneys (n = 6 per group). (K) CCT3 protein levels in the kidneys of AAV9‐NC and AAV9‐CCT3 mice (n = 3 per group). (L) Representative images of mouse tissues stained with HE and Masson in indicated groups. Scale bar, 50 µm. (M) Representative western blot for fibrotic and EMT markers in indicated groups (n = 6 per group). (N) mRNA levels of fibrotic and EMT genes in indicated groups (n = 6 per group). Gene expression levels were normalized to *Gapdh*. (O) mRNA levels of *Ctnnb1*, *Ccnd1* and *Myc* genes in indicated groups (n = 6 per group). Gene expression levels were normalized to *Gapdh*. (P) Representative western blot for CTNNB1, CCND1, and MYC markers in groups (n = 3 per group). Data were presented as mean ± SEM, and statistical differences were determined by one‐way ANOVA.

Given that DCL binding to IQGAP1 blocks the interaction between IQGAP1 and CCT3, thereby inhibiting Wnt signaling pathway activation, we evaluated the effect of IQGAP1 or CCT3 in UUO‐induced renal fibrosis in mice. We first generated mice with tubular epithelial cells‐specific deletion of *Iqgap1* using adeno‐associated virus serotype 9 encoding IQGAP1 (kspAAV9‐IQGAP1) and AAV9‐control (NC), the virus was injected in situ in the cortex of the kidney in mice (Figure 10A). The mRNA and protein expression of IQGAP1 were significantly decreased in the kidneys after injection of AAV9‐IQGAP1 for 5 weeks (Figure 10B,C). IQGAP1 knockdown inhibited the UUO‐induced tubular expansion, and collagen deposition both in the cortex and medulla of the kidneys (Figure 10D). UUO‐stimulated EMT progression, pro‐inflammatory cytokines, adhesion molecules, and fibrogenic factors expressions were also reversed by IQGAP1 knockdown (Figure 10E,F, Figure S10A,B). In CCT3 knockdown mice induced by kspAAV9 in situ injection, macrophage infiltration, EMT marker gene expression, pro‐inflammatory cytokines, and fibrogenic factors were noticeably reversed (Figure 10I–N, Figure S10C,D). As expected, the activation of Wnt signaling pathway in kidneys of UUO mice was inhibited by IQGAP1 or CCT3 knockdown (Figure 10G,H, O,P).

## 3 Discussion

In this study, we developed a novel multimodal AI‐driven TCM‐SPred model that synergistically integrates deep neural networks with multi‐omics biological feature fusion. Through systematic virtual screening of TCM compounds, TCM‐SPred prioritized Aucklandiae Radix as a top candidate for renal fibrosis intervention. Subsequently, a natural guaianolide sesquiterpene lactone derivative DCL, the main bioactive constituent in Aucklandiae Radix, effectively ameliorated renal injury and fibrosis in vitro and in vivo. The major findings of this study include the following: (i) We utilized TCM‐SPred to discover that Aucklandiae Radix possessed anti‐renal fibrosis effects. (ii) We identified that DCL from Aucklandiae Radix effectively ameliorated UUO‐ and FA‐induced renal fibrosis and injury in mice. Additionally, DCL mitigated fibrosis and EMT progression in tubular epithelial cells in response to TGF‐β1. (iii) Mechanistically, DCL directly targeted IQGAP1 to inhibit Wnt signaling pathway by blocking the interaction between IQGAP1 and CCT3. These findings highlighted the potential of DCL as a promising therapeutic agent for renal injury and fibrosis, underscoring its ability to mitigate CKD by targeting the IQGAP1‐CCT3‐Wnt signaling axis.

The AI technology has been increasingly applied to discover novel therapeutic agents and accelerate the process of drug discovery. Herein, we proposed an AI model, TCM‐SPred, that integrates semantic embedding from Word2Vec, sequence modeling driven by BiLSTM, and cross‐modal attention‐guided interaction. Through the predictions of TCM‐SPred, the complex herbal and symptom associations could be elucidated, leading to the acquisition of therapeutic herbs for related symptoms. The excellent performance of TCM‐SPred was further validated by stable parameter convergence (TP rate > 97.16%) and robust cross‐validation (AUC: 0.92–0.97). Furthermore, the TCM‐SPred model prediction revealed that the TCM herb Aucklandiae Radix exhibited a high herb‐symptom correlation score in the treatment of renal fibrosis (score = 13.68, Figure 2C), supporting its potential as a therapeutic agent for renal fibrosis.

Aucklandiae Radix is an important traditional Chinese medicinal herb, and 212 compounds had been identified in Aucklandiae Radix, including sesquiterpene lactones, monoterpenoids, triterpenoids, phenylpropanoids, steroids, flavonoids, and amino acids [27]. Among them, DCL and CT were considered the primary constituents and served as quality control benchmarks for Aucklandiae Radix, with their content not being less than 1.8%. Although numerous pharmacological activities of DCL have been reported, such as anti‐inflammatory and anticancer effects [28, 29], the potential of DCL in the treatment of renal fibrosis and its underlying mechanisms remains unexplored.

To elucidate the underlying mechanism, we utilized an affinity‐based protein profiling (ABPP)‐based chemical proteomic strategy to identify the protein targets of DCL. Our investigations revealed 334 proteins that may interact with DCL. Among them, the top 10 interacting proteins were FASN, XRCCS, IQGAP1, LRPPRC, EIF4G1, EPRS, SF3B3, RPN2, LMAN1, and EIF2S1. Notably, IQGAP1 has emerged as a critical player in kidney diseases [30]. IQGAP1 belongs to the scaffold protein family and interacts with signaling and structural molecules to regulate various cellular functions, such as adhesion, migration, and integration of complex signaling pathways [31]. Previous studies have shown that silencing IQGAP1 inhibits the recruitment of bone marrow mesenchymal stromal cells and alleviates hepatic fibrosis [32]. Additionally, IQGAP1 has been implicated as a scaffold protein contributing to bleomycin‐induced pulmonary fibrosis, strongly suggesting its involvement in the pathophysiology of systemic sclerosis‐related interstitial lung disease by increasing contractile forces and lung stiffness [20]. However, it remains unclear whether IQGAP1 plays a role in renal fibrosis and tubular epithelial cell EMT. Given IQGAP1's pivotal role in kidney diseases and tissue fibrosis, we selected it for subsequent validations. SPR analysis demonstrated that DCL directly binds to the recombinant IQGAP1 protein, which was further corroborated by CETSA, fluorescence assays, and pull‐down experiments. The LC‐MS/MS result revealed that DCL covalently binds to Cys1534 in IQGAP1. These findings collectively identified IQGAP1 as a direct target of DCL, highlighting its potential role in mediating DCL for the treatment of renal fibrosis.

The transcriptomic and IP‐MS analyses revealed that DCL suppressed the Wnt signaling pathway by disrupting the interaction between IQGAP1 and CCT3. CCT3, also referred to as CCT‐γ, PIG48, TRIC5, or TCP‐1‐γ, is a critical subunit of the chaperonin‐containing TCP‐1 complex (CCT), playing a vital role in protein folding [33]. Recently, abnormal expression of CCT3 has been associated with various cancers, correlating with prognosis and therapeutic outcomes. Previous studies have confirmed that multiple factors and pathways are involved in CCT3‐mediated cancer progression, including STAT3, P53, NF‐κB, AKT/mTOR, and Wnt/β‐catenin signaling pathways [34, 35, 36, 37, 38]. The Wnt signaling pathway not only contributes to tumor cell dedifferentiation and proliferation but also affects target genes such as fibroblast‐specific protein 1 (FSP‐1), fibronectin, matrix metalloproteinase 7 (MMP7), Snail, and Twist, which play significant roles in regulating the EMT process in renal fibrosis settings [39, 40, 41, 42, 43]. Therefore, targeting the CCT3‐Wnt axis may represent a novel and promising therapeutic strategy for the treatment of renal fibrosis.

Despite the encouraging findings, our work still has some limitations: (1) Whether other top DCL‐interacting proteins also play a role in renal fibrosis needs further validation. (2) The Cre/loxP‐dependent conditional gene‐targeting approach should be used to test for DCL‐mediated activities through IQGAP1 or CCT3 interactions. (3) The crystal structure of IQGAP1 with DCL, a direct means to decipher protein‐ligand interactions, remains a challenging feat. (4) We have shown that DCL interacted with IQGAP1, but the specific binding domain and sites for IQGAP1 are not characterized.

In conclusion, this study identifies a positive role of DCL in the treatment of renal fibrosis and proposes a potential therapeutic approach by targeting the IQGAP1‐CCT3‐Wnt axis to treat renal fibrosis.

## 4 Methods

### 4.1 Establishment Procedures of the Multimodal AI‐driven TCM‐SPred

See the Supplementary Information for details.

### 4.2 Synthesis of Probes and Data Assay

See the Supplementary Information for details.

### 4.3 Plant Material and Extraction

The dried rhizomes of Aucklandiae Radix were pulverized into coarse particles and subjected to sequential extraction with petroleum ether. Briefly, the powdered material (5 kg) was macerated with 8 volumes (v/w) of solvent for 12 h at 25°C, followed by two cycles of hot reflux extraction (80°C, 2 h per cycle) using a condenser‐equipped apparatus. The resultant extracts were filtered through qualitative filter paper, pooled, and concentrated under reduced pressure using a rotary evaporator. The concentrate was subsequently desiccated in a vacuum drying oven to yield the petroleum ether extract (ARE, mean yield: 3.52 ± 0.7% w/w). The crude extract was subsequently fractionated via silica gel flash column chromatography with a gradient elution system of petroleum ether‐ethyl acetate. Fractions enriched in DCL were identified by thin‐layer chromatography, pooled, and concentrated under reduced pressure at 40°C. Final purification was achieved through recrystallization from methanol‐chloroform at 4°C, yielding crystalline DCL.

### 4.4 Animal

Male C57BL/6J mice (SPF grade; 6–8 weeks old; 22–26 g) were obtained from Jiangsu Jicui Yaokang Biotechnology Co., Ltd. (Jiangsu, China). The animals were housed in individually ventilated cages under controlled environmental conditions (temperature: 20°C–26°C; relative humidity: 30%–70%) for a 7‐day acclimatization period. All experimental protocols were conducted in compliance with the Guidelines for the Care and Use of Laboratory Animals and approved by the Animal Ethics Committee of Nanjing University of Chinese Medicine (Ethics Approval No. 202105A005).

### 4.5 UUO‐Induced Renal Fibrogenesis Model

Male mice were anesthetized by intraperitoneal injection of pentobarbital sodium (30 mg kg^−1^). The left ureter was incised and ligated with 5–0 silk suture. The abdominal wall was closed with continuous sutures followed by interrupted skin closure. Postoperative animals were maintained in a temperature‐controlled environment with 12/12‐h light/dark cycles, receiving standard rodent chow and water ad libitum.

### 4.6 FA‐Induced Renal Fibrogenesis Model

Male mice (8 weeks old) were intraperitoneally administered a single dose of folic acid (250 mg kg^−1^) dissolved in 0.3 M sodium bicarbonate solution. Age‐matched controls received equivalent volumes of bicarbonate vehicle (normal saline) using identical injection protocols.

### 4.7 The Petroleum Ether Extract of Aucklandiae Radix Treatment In Vivo

The petroleum ether extract of Aucklandiae Radix (100 mg kg^−1^ day^−1^) was orally administered based on interspecies dose conversion protocols and established pharmacological precedents. Male C57BL/6J mice were subjected to UUO procedures. In the UUO paradigm, daily delivery of the botanical extract or saline vehicle was initiated on the surgical day and maintained for 12 consecutive postoperative days.

### 4.8 DCL Treatment In Vivo

In the UUO experiment, mice were orally administered the vehicle, 0.1, 1, or 10 mg kg^−1^ DCL once daily for 12 consecutive days.

In the FA experiment, mice were orally administered the vehicle, 0.1, 1, or 10 mg kg^−1^ DCL once daily for 3 consecutive days.

For the assessment of the protective (prophylactic) effect of the petroleum ether extract of Aucklandiae Radix against renal fibrosis, the male C57BL/6J mice were randomly allocated into Sham, UUO, and the petroleum ether extract of Aucklandiae Radix (100 mg kg^−1^ d^−1^) groups based on body weight, with 6 mice in each group. For the involvement of IQGAP1 expressed in colonic macrophages in DCL‐mediated alleviation of colitis, female C57BL/6J mice were randomly allocated into normal group, adeno‐associated virus (AAV)‐scramble plasmid group, AAV‐IQGAP1 plasmid group, DCL (10 mg kg^−1^ d^−1^) + AAV‐IQGAP1 plasmid group, DCL (10 mg kg^−1^ d^−1^) + AAV‐scramble plasmid group and PFD (100 mg kg^−1^ d^−1^) groups.

### 4.9 ELISA Assay

Serum creatinine (Cr) and blood urea nitrogen (BUN) concentrations were measured using commercial assay kits (Cr: E‐BC‐K188‐M; BUN: E‐BC‐K183‐M) according to the manufacturer's protocols. Both kits were procured from Wuhan Elabscience Biotechnology Co., Ltd.

### 4.10 Immunohistochemical Analysis

Renal tissues from mice were fixed in 4% paraformaldehyde for at least 24 h and paraffin‐embedded. Sections were dewaxed through xylene and rehydrated via graded ethanol series prior to peroxidase anti‐peroxidase (PAP) staining. Antigen retrieval was achieved by microwave heating in 10 mM citrate buffer (pH 6.0) or Tris‐EDTA buffer (pH 9.0). Endogenous peroxidase activity was quenched using 3% H_2_O_2_. Primary antibody incubation was conducted overnight at 4°C, followed by incubation with a species‐matched secondary antibody. Diaminobenzidine (DAB) served as chromogen with hematoxylin counterstaining. Slides were permanently mounted using neutral balsam after graded alcohol dehydration. Whole‐slide imaging was performed using Hamamatsu NanoZoomer 2.0 RS scanner.

### 4.11 Real‐Time Polymerase Chain Reaction

Total RNA was extracted from renal tissues or HK‐2 cells using FreeZol reagent (R711, Vazyme, Nanjing, China) per manufacturer's protocol. RNA was reverse‐transcribed with HiScript II Q RT SuperMix (+qDNA wiper, R223, Vazyme) following product guidelines. cDNA aliquots were cryopreserved at −20°C until qPCR analysis. Real‐time quantitative PCR was performed using ChamQ SYBR Master Mix (Without ROX, Q321, Vazyme) according to kit specifications. Data normalization was performed using Gapdh as the endogenous control, followed by ΔΔCt method calculations. Primer sequences are cataloged in Table S5.

### 4.12 Culture and Treatment of Cells

HK‐2 cells were procured from the Cell Bank of Jiangsu Kaiji Biotechnology Co., Ltd. (Jiangsu, China) and maintained in DMEM/F12 basal medium supplemented with 10% fetal bovine serum (FBS; Gibco) and 1% penicillin‐streptomycin cocktail. TCMK‐1 cells were procured from the Cell Bank of Jiangsu Kaiji Biotechnology Co., Ltd. (Jiangsu, China) and maintained in DMEM basal medium supplemented with 10% fetal bovine serum (FBS; Gibco) and 1% penicillin‐streptomycin cocktail. HK‐2/TCMK‐1 cells were treated with 10 ng mL^−1^ recombinant human TGF‐β1 protein (with or without DCL) for 24 h.

NIH‐3T3 cells were procured from the Cell Bank of Jiangsu Kaiji Biotechnology Co., Ltd. (Jiangsu, China) and maintained in RPMI 1640 basal medium supplemented with 10% fetal bovine serum (FBS; Gibco) and 1% penicillin‐streptomycin cocktail. NIH‐3T3 cells were treated with 10 ng mL^−1^ recombinant human TGF‐β1 protein (with or without DCL) for 24 h.

L929 cells were obtained from the American Type Culture Collection (ATCC). They were cultured in RPMI 1640 medium supplemented with 10% fetal bovine serum (FBS; Gibco) and 1% penicillin‐streptomycin cocktail, and maintained at 37°C in a humidified 5% CO_2_ incubator. L929 cells were seeded into 10‐cm dishes under the same incubation conditions (37°C, 5% CO_2_). Once the cells reached 70%–80% confluence, 12 mL of fresh RPMI 1640 medium containing 10% FBS was added. After 5–6 days of culture, the conditioned supernatant was collected into a 15‐mL centrifuge tube, stored at −80°C, and filtered through a 0.22 µm filter immediately prior to use.

After discarding the supernatant, the precipitate was re‐suspended in RPMI 1640 medium containing 20% conditioned medium from L929 cells and incubated in an incubator for 3 days. Subsequently, half of the old culture medium was replaced with fresh complete media (containing 20% conditioned medium from L929 cells), and the cells were cultured for another 4 days. On day 7, the BMDMs were ready for subsequent experiments.

Isolation and Differentiation of BMDMs: mice were euthanized by cervical dislocation, and their skin was disinfected by immersion in 75% ethanol. Both femurs were carefully excised, and adhering muscle tissue was removed. Bone marrow cells were flushed from the bones using pre‐chilled, serum‐free RPMI 1640 medium and collected into a 15 mL conical tube. Following centrifugation at 1600 rpm for 5 min at 4°C, the supernatant was discarded, and the cell pellet was resuspended in complete RPMI 1640 medium (supplemented with 10% FBS and 1% penicillin‐streptomycin). Cells were then plated and allowed to adhere for 12 h; non‐adherent cells were discarded, and the remaining adherent cells were cultured in RPMI 1640 medium supplemented with 20% L929 cell‐conditioned medium. The culture medium was replaced every three days. By day 7, the adherent cells had differentiated into BMDMs and were ready for use. BMDMs were first treated with 10 ng mL^−1^ recombinant human TGF‐β1 for 3 days, followed by treatment with or without DCL in fresh medium for an additional 24 h.

HEK‐293T cells were procured from the Cell Bank of Jiangsu Kaiji Biotechnology Co., Ltd. (Jiangsu, China) and maintained in DMEM basal medium supplemented with 10% fetal bovine serum (FBS; Gibco) and 1% penicillin‐streptomycin cocktail.

### 4.13 Co‐Immunoprecipitation

For co‐immunoprecipitation experiments, cells were washed with ice‐cold PBS and lysed in pre‐chilled lysis buffer (P0013, Beyotime Biotechnology, China) on ice for 30 min. Lysates were centrifuged at 12,000 × *g* for 10 min, and supernatants were collected. A portion of the supernatant was reserved as an input control, while the remaining supernatant was incubated with antibodies or IgG under rotation at 4°C overnight. The following day, the supernatant‐antibody mixture was incubated with 20 µL protein A/G agarose beads at room temperature for 4 h. After PBS washing, 2× loading buffer was added to the complexes. The mixture was boiled for 5 min, and the supernatant was subjected to Western blotting. The following antibodies were employed: IQGAP1 (1:100, 12995‐1‐AP, Proteintech, China); CCT3 (1:100, SC‐376830, Santa Cruz Biotechnology, USA); HA‐Tag (1:100, AE105, Abclonal, China); IgG (A7017/A7028, Beyotime Biotechnology, China); IgG Light Chain (HRP) (1:1000, A25022/A25012, Abbkine, China).

To validate interacting proteins of IQGAP1 or CCT3, immunoprecipitation followed by Western blotting was performed. Cells were transfected with IQGAP1‐ or CCT3‐expressing plasmids for 24 h, followed by an additional 24‐h treatment with recombinant TGF‐β1 (10 ng mL^−1^) in the presence or absence of DCL.

### 4.14 Western Blotting

Total proteins were extracted from cells by incubating with NP‐40 buffer (P0013F, Beyotime Biotechnology, China) supplemented with protease inhibitors (ST507, Beyotime Biotechnology) on ice for 30 min to prepare cell lysates. The lysates were then centrifuged at 12 000 × *g* for 10 min at 4°C. Supernatants were carefully collected and mixed with 4 × SDS‐PAGE loading buffer. An equal amount of protein was loaded on 10% SDS‐PAGE wells and transferred onto PVDF membranes (1620177, Millipore, Massachusetts, MA, USA). Then, the membranes were blocked with Tris‐buffered saline containing 0.05% Tween‐20 (TBST) and 5% nonfat milk for 2 h on decoloring shaker and probed with different primary antibodies at 4°C overnight. After washing with TBST for three times, membranes were incubated with the indicated horseradish peroxidase (HRP)‐linked anti‐rabbit (7074, 1: 1000, CST) or anti‐mouse (7076, 1:1000, CST) antibody for 1 h at room temperature. The final detection was performed by Super Signal West Femto (34094, Thermo Fisher Scientific).

### 4.15 Antibodies

See the Supplementary Information for Details

### 4.16 Activity‐Based Protein Profiling (ABPP) Target Enrichment

HK‐2 cells stimulated with TGF‐β1 were lysed via ultrasonication, and protein extracts with concentrations exceeding 2 mg mL^−1^ were incubated with probes for 1 h. Subsequently, 10 mM Biotin‐azide (5.7 µL), 50 mM TCEP (11.3 µL), 3.4 mM TBTA (17 µL), and 50 mM CuSO4 (11.3 µL) were sequentially added, followed by a 1.5 h reaction at 25°C. Pre‐chilled acetone was introduced to precipitate proteins, which were then resolubilized in PBS containing 0.5% SDS and incubated with streptavidin‐agarose beads to enrich biotinylated probe‐binding proteins. The beads were subjected to five sequential washes with PBS containing 0.5% SDS and four washes with PBS. Captured proteins underwent reduction/alkylation and tryptic digestion prior to analysis using a Nano LC‐Q Exactive Plus system (Thermo Fisher Scientific). Chromatographic separation was performed on a PicoFrit Emitter column (75 µm ID, packed with Reprosil‐Pure‐AQ C18 phase, 1.9 µm particle size, 16 cm length) at 200 nL min^−1^ with mobile phase A (0.1% formic acid in H2O) and B (0.1% formic acid in 80% ACN) using a 120‐min gradient (3%–32% B over 95 min, 32%–100% B over 10 min, followed by 15 min isocratic elution). Mass spectrometry parameters included a nano‐electrospray ion source (2.2 kV spray voltage, 275°C capillary temperature), full MS scans (355–1700 m/z) at 70 000 resolutions (AGC target 5×105, max injection time 100 ms), and data‐dependent MS2 scans using HCD at 35 000 resolution (AGC target 5×104 max injection time 75 ms, 1.6 m/z isolation window) with a 60 s dynamic exclusion. Raw data were processed in Proteome Discoverer 2.2.0 (Thermo Fisher Scientific) against the UniProt human proteome database (including common contaminants), with remaining parameters set to default configurations.

### 4.17 Investigative Study on Human Renal Biopsy

The human studies were approved by the Ethics Committee of the Renmin Hospital of First Affiliated Hospital of Nanjing Medical University in accordance with the World Medical Association's Declaration of Helsinki (2024‐SR‐299). Written informed consent was obtained from all participants before recruitment.

### 4.18 Histopathologic Examination

Renal tissues were fixed in 4% paraformaldehyde for 24 h, embedded in paraffin, and sectioned into 4–5 µm slices. The sections were stained with hematoxylin and Masson's trichrome, followed by prompt imaging to quantitatively assess the severity of renal fibrosis and semiquantitatively evaluate histopathological alterations.

### 4.19 Cellular thermal shift assay (CETSA)

HK‐2 cells cultured in 10‐cm dishes were treated with compounds or DMSO for 1 h. The cells were harvested and resuspended in 1 mL PBS supplemented with protease inhibitors. The cell suspension was aliquoted into seven 0.2 mL PCR tubes (100 µL per tube). Samples were heated at designated temperatures (e.g., 37, 42, 47, 52, 57, 62, 67, and 70°C) for 3 min followed by incubation at 25°C for 3 min using a 96‐well thermal cycler (Bio‐Rad, USA). Three freeze‐thaw cycles were performed in liquid nitrogen to lyse the cells. Tubes were briefly vortexed after each thawing step. Supernatants from the cell lysates were collected and subjected to Western blot analysis.

### 4.20 Solvent‐Induced Protein Precipitation Assay (SIP)

The pretreatment protocol for HK‐2 cells was followed as described in the aforementioned methodology. Supernatants were divided into seven equal aliquots, and proteins in the supernatants were precipitated with an organic solvent mixture (A.E.A: acetone/ethanol/acetic acid = 50:50:0.1). Final organic solvent concentrations were adjusted to 0%, 9%, 11%, 13%, 15%, 17%, and 19% using the A.E.A mixture, followed by incubation at 37°C with agitation at 800 rpm for 30 min. Centrifugation was performed at 15 000 × *g* for 10 min at 4°C using a refrigerated centrifuge (Eppendorf, Hamburg, Germany). The resulting supernatants were collected and analyzed for IQGAP1 protein expression via Western blotting.

### 4.21 Surface Plasmon Resonance (SPR)

SPR measurements were performed using a Biacore T200 instrument (GE Healthcare, Boston, MA, USA). Recombinant IQGAP1 protein was immobilized on a CM5 sensor chip via amine coupling chemistry according to the manufacturer's standard protocol. The protein was diluted in sodium acetate buffer (pH 4.5) prior to immobilization. DCL samples at varying concentrations were prepared in running buffer (1.05× PBS‐P, 5% DMSO, pH 7.4) and injected over the Fc2‐Fc1 flow cells. The SPR parameters were set as follows: flow rate of 30 µL/min, contact time of 120 s, dissociation time of 180 s, and temperature maintained at 25°C. Binding kinetics were analyzed using the Biacore T200 Evaluation Software (GE Healthcare, Boston, MA, USA), with the equilibrium dissociation constant calculated from steady‐state affinity models.

### 4.22 Immunofluorescence Staining

HK‐2 cells and renal tissue sections were fixed in 4% paraformaldehyde (PFA) in PBS at room temperature for 20 min. After three washes with ice‐cold PBS, permeabilization was performed using 0.1% Triton X‐100 for 20 min, followed by three additional washes with pre‐chilled PBS. Non‐specific binding sites were blocked by incubating samples with 1% bovine serum albumin (BSA; P0102, Beyotime Biotechnology, Shanghai, China) for 2 h at room temperature. Subsequently, samples were incubated overnight at 4°C with either α‐SMA antibody or IQGAP1 antibody, all antibodies were diluted in 1% BSA. On the following day, samples were incubated with Alexa Fluor 488‐conjugated goat anti‐rabbit and Alexa Fluor Cy3‐conjugated goat anti‐mouse secondary antibodies in the dark for 1 h. After three washes with PBS, nuclear counterstaining was performed by applying 4′,6‐diamidino‐2‐phenylindole (DAPI; G1012, Servicebio Technology Co., Ltd., Wuhan, China) for 5 min. Fluorescence images were acquired using a Leica TCS SP8 X laser‐scanning confocal microscope (Leica Microsystems, Germany) equipped with a 63×oil immersion objective (NA 1.4), with laser excitation/emission filters optimized for DAPI, Alexa Fluor 488, and Alexa Fluor Cy3. Z‐stack images were captured at 0.5 µm intervals and processed using Leica LAS X software (v3.7) for maximum intensity projection.

### 4.23 Immunohistochemistry Analysis

Paraffin‐embedded mouse or human kidney sections (6 µm thickness) were deparaffinized with xylene and rehydrated through a graded ethanol series (100%, 95%, 90%, 80%, 75%) followed by distilled water. Endogenous peroxidase activity was blocked by incubation with 3% hydrogen peroxide for 15 min. Antigen retrieval was performed by heating the sections in sodium citrate buffer. Sections were subsequently blocked with 10% goat serum at room temperature for 1 h, followed by overnight incubation with primary antibodies at 4°C. After four washes with PBS, the sections were incubated with species‐matched HRP‐conjugated secondary antibodies at room temperature for 1 h, followed by additional PBS washes. Immunoreactivity was visualized using 3,3′‐diaminobenzidine (DAB) chromogen (K5007, Dako, Denmark). The slides were counterstained with hematoxylin and permanently mounted with neutral balsam. Digital image acquisition was subsequently performed using an Olympus BX53 brightfield microscope (Tokyo, Japan).

### 4.24 IP‐MS Analysis

Protein extracts for immunoprecipitation were prepared using non‐denaturing immunoprecipitation buffer supplemented with protease inhibitor cocktail, following 24‐h treatment with or without 10 ng/mL TGF‐β1. After protein quantification, anti‐IQGAP1 primary antibody was added to the lysates, and target proteins were immunoprecipitated using protein A/G magnetic beads. The beads were washed, and the eluted proteins were separated on 10% SDS‐PAGE gels. Subsequently, in‐gel digestion was performed, and the resulting peptides were analyzed by LC‐MS/MS. Detailed data are provided in the Supplementary Table.

### 4.25 Lc‐Ms/Ms

The recombinant IQGAP1 protein was incubated with DCL at 37°C for 1 h. Next, a series of operations including reduction and alkylation, enzymatic digestion, collection of peptide fragments, and desalination were carried out in sequence. Next, peptide sequences of IQGAP1 were analyzed using an LC‐MS/MS instrument and Max Quant software. Then, the amino acid sequence of IQGAP1 was compared with the known protein sequence to determine the binding site of the compound to the target protein.

### 4.26 Molecular Docking

The X‐ray crystal structure of IQGAP1 was retrieved from the Protein Data Bank (PDB) and preprocessed using the Protein Preparation Wizard module in Schrödinger Maestro 2019. The protein preprocessing workflow was identical to the reverse docking protocol described above. A binding pocket was defined as residues within a 20 Å radius of Cys1534. The ligand DCL was first constructed using ChemBio3D Ultra software (PerkinElmer, USA) and subsequently prepared via the Ligand Preparation module in Schrödinger Maestro 2019. Covalent docking of DCL to IQGAP1 was performed using the Glide Covalent Docking protocol. The Glide docking score was selected as the primary ranking criterion, with the number of output poses per ligand set to 5. Critically, the covalent reaction type was specified as Michael addition, and the covalently modified residue was explicitly assigned to Cys1534.

### 4.27 Proximity Ligation Assay (PLA)

The interaction between IQGAP1 and CCT3 in HK‐2 cells was detected using the PLA. HK‐2 cells were cultured in confocal dishes and subjected to modeling/drug treatment protocols, followed by fixation with 4% PFA in PBS for 20 min. Fixed cells were processed following the PLA protocol according to the manufacturer's instructions. In situ PLA was performed as specified in the manufacturer's manual. Amplified fluorescence signals were visualized using a Zeiss LSM 800 confocal laser scanning microscope.

### 4.28 SiRNA and Plasmids Transfection

IQGAP1 siRNA, CCT3 siRNAs or negative control siRNA were separately transfected into HK‐2 cells using Lipofectamine 2000. Briefly, HK‐2 cells were seeded in antibiotic‐free complete DMEM/F12 medium within 6‐well plates. Upon reaching 50% confluency, the medium was replaced with Opti‐MEM (31985070, Gibco) containing 120 nM IQGAP1‐siRNA and 5 µL Lipofectamine 2000. Following a 6‐hour incubation, cells were transferred to complete DMEM/F12 medium and cultured until 80% confluency. TGF‐β1 and DCL were subsequently added to the medium, and cells were harvested for Western blotting after 24 h. The same protocol was applied to HEK293T cells transfected with si‐IQGAP1. The sequences of siRNA are as follows:

IQGAP1‐siRNA‐1: CAAUGAUCCAAUCCACGAATT;

IQGAP1‐siRNA‐2: GGCAUAUCAAGAUCGGUUATT;

IQGAP1‐siRNA‐3: CCAGUAAUCUACAUUUCCAUUTT;

CCT3‐siRNA‐1: GGAAAGAGAUUGACAUAAATT;

CCT3‐siRNA‐2: GAGCAGGCCUGUUGGAAAUTT;

CCT3‐siRNA‐3: CUGUGAAGCUGCAGACUATT;

HK‐2 cells were seeded in 10 cm culture dishes containing DMEM/F12 medium supplemented with 10% fetal bovine serum (FBS) until reaching 80% confluency. Fresh medium was added 2 h prior to transfection. A mixture of 10 µL NEOFECT DNA transfection reagent and 10 µg plasmid DNA (pc DNA 3.1‐based HA‐IQGAP1, Flag‐CCT3, IQGAP1‐C1534A mutant, or IQGAP1‐WT) was incubated for 10 min at room temperature and subsequently introduced to the cells. Following 10‐hour incubation, cells were subjected to experimental treatments as indicated.

### 4.29 Microinjection of Adeno‐Associated Virus Into Mouse Kidneys

AAV serotype 9 vectors encoding a green fluorescent protein (GFP) reporter and shRNA targeting IQGAP1(AAV‐shIQGAP1), CCT3 (AAV‐shCCT3), or a control shRNA (AAV‐control) were designed for renal‐specific knockdown. AAVs were microinjected into at least six distributed sites within the renal cortex of mice under stereotaxic guidance. Kidneys were harvested 35 days post‐injection, and efficiency knockdown was quantified by Western blotting.

A single 60 µL dose of AAV2/9‐Ksp‐cadherin‐mir30‐m‐EGFP‐IQGAP1/CCT3 short hairpin RNA (shRNA) viral suspension (viral titer > 1012) or AAV2/9‐Ksp‐cadherin‐miR‐30‐m‐EGFP‐control shRNA (Guangzhou Pai Zhen Biotechnology Co., Ltd.) was injected into the renal cortex of mice via in situ microinjection. Kidneys were collected 35 days post‐injection, and knockdown efficiency was quantified by Western blotting using anti‐IQGAP1/anti‐CCT3 antibodies. The shRNA oligonucleotide sequences were as follows:

mIQGAP1‐shRNA‐F: 5′‐CAGGCACATGCCGAGAATAAT‐3′;

mIQGAP1‐shRNA‐F: 3′‐ATTATTCTCGGCATGTGCCTG‐5′;

mCCT3‐shRNA‐F: 5′‐GGAACCCCTAGTGATGGAGTT‐3′,

mCCT3‐shRNA‐R: 5′‐CGGCCTCAGTGAGCGA‐3′.

### 4.30 Statistics Analysis

Statistical analyses were performed using GraphPad Prism 9.0.0. Data are presented as the mean ± standard error of the mean. All data were analyzed by two‐tailed *t*‐tests or one‐way ANOVA (clinical samples) as appropriate, and *p‐*values < 0.05 was considered statistically significant.

## Author Contributions

W.J.L., W.Z.X., P.W., and K.L. designed and performed the experiments and analyzed the data. Z.Z.Z., W.Y.L., X.Q.P., X.K.Y., S.L.M., Z.Z, A.P., and J.L. performed the experiments and analyzed the data. L.H.H. initiated the study, designed and performed the experiments, analyzed the data, and wrote the paper. All authors approved the paper.

## Conflicts of Interest

The authors declare no conflicts of interest.

## Supporting information




**Supporting File**: advs74192‐sup‐0001‐SuppMat.docx

## Data Availability

Research data are not shared.
