# Supplementary material for: Multimodal AI‐Driven Identification of Dehydrocostus Lactone as a Potent Renal Fibrosis Attenuator Targeting IQGAP1
Source: Adv Sci (Weinh). 2026 Feb 3;13(20):e20277. doi: 10.1002/advs.202520277 (PMC13067758; doi:10.1002/advs.202520277)

**Supporting Information**

**Multimodal AI-driven Identification of Dehydrocostus Lactone as a Potent Renal Fibrosis Attenuator targeting IQGAP1**

Weijiang Lin ^a, #^, Wenzhuo Xu ^a #^, Kang Liu ^c, #^, Ping Wang ^a, #^, Zhenzhen Zhu ^a^, Wenyu Lu ^b^, Zhe Zheng ^a^, Xiaoqian Peng ^b^, Xunkai Yin ^a^, Shulan Mei ^a^, An Pan ^a, *^, Jian Liu ^a, b, *^, Lihong Hu ^a, d, *^

a Jiangsu Key Laboratory for Functional Substance of Chinese Medicine, School of Pharmacy, Nanjing University of Chinese Medicine, Nanjing 210023, PR China

b School of Artificial Intelligence and Information Technology, Nanjing University of Chinese Medicine, Nanjing 210023, PR China

c Department of Nephrology, the First Affiliated Hospital of Nanjing Medical University (Jiangsu Province Hospital), Nanjing Medical University, Nanjing 210003, PR China

d China Joint Graduate School of Traditional Chinese Medicine, Nanjing 210023, PR China

*Corresponding Authors: panan@njucm.edu.cn (Pan An); liujian623@njucm.edu.cn (Jian Liu); lhhu@njucm.edu.cn (Lihong Hu)

**
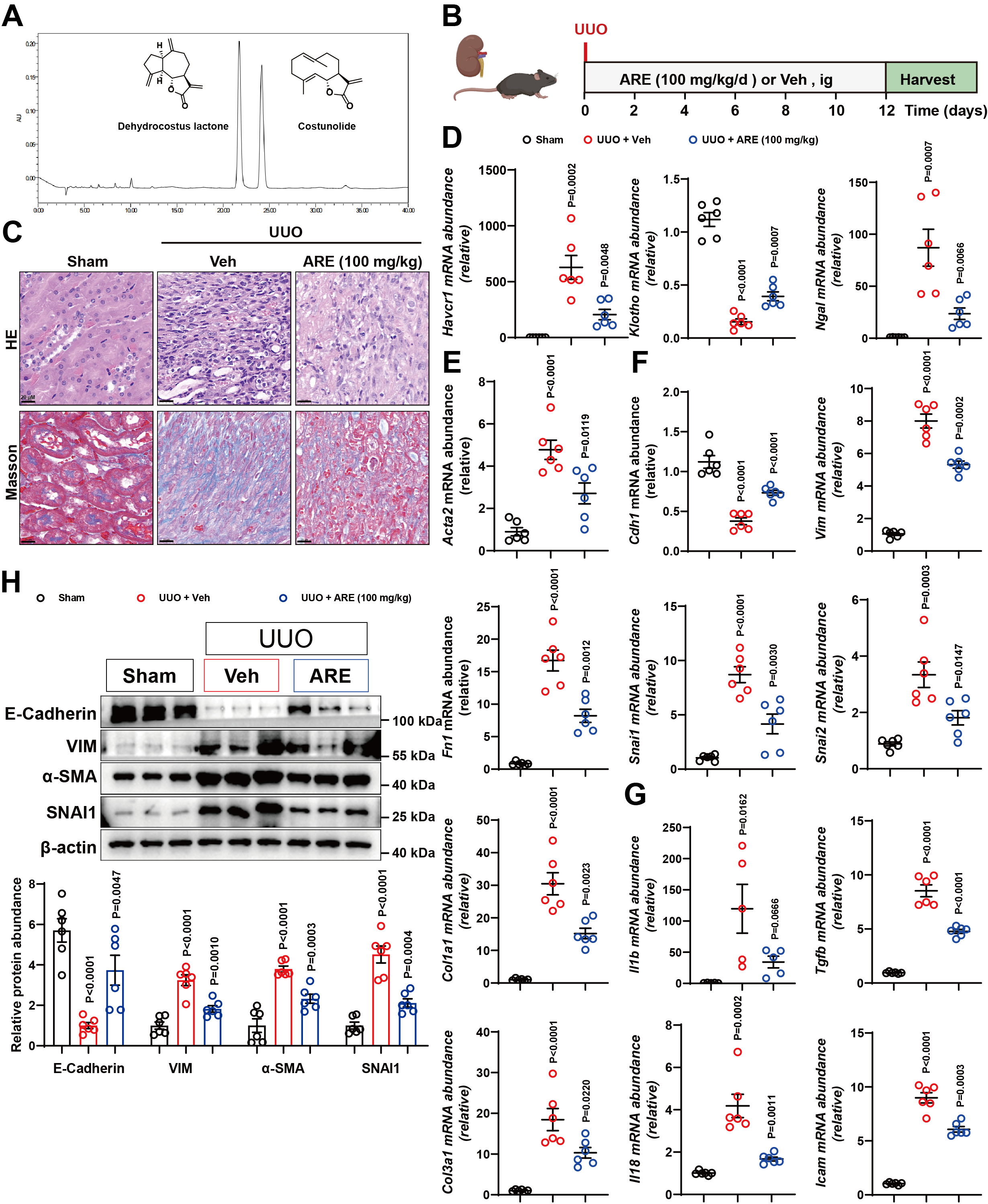
**

**Figure S1 Sesquiterpene Lactones from** **Aucklandiae Radix Alleviate UUO-Induced Renal Fibrosis in Mice. A** The abundance of sesquiterpene lactone in Aucklandiae Radix. **B** the scheme of administration the extraction of Aucklandiae Radix in renal fibrosis induced by UUO. **C** Representative images of mouse renal tissues stained with HE and Masson in indicated groups. Scale bar, 50 μm. **D-G**, qRT-PCR analysis of injury- (**D**), fibrosis- (**E**), epithelial-mesenchymal transition (EMT)- (**F**), and inflammation- (**G**) related mRNA expression in renal tissues from above mice (n = 6 per group). **H**, Kidney homogenate samples were analyzed by western blotting (n = 6 per group) to quantify the protein levels of E-Cadherin, VIM, α-SMA and SNAI1. All statistic data were presented as mean ± SEM and statistical differences were determined by one-way ANOVA.

**
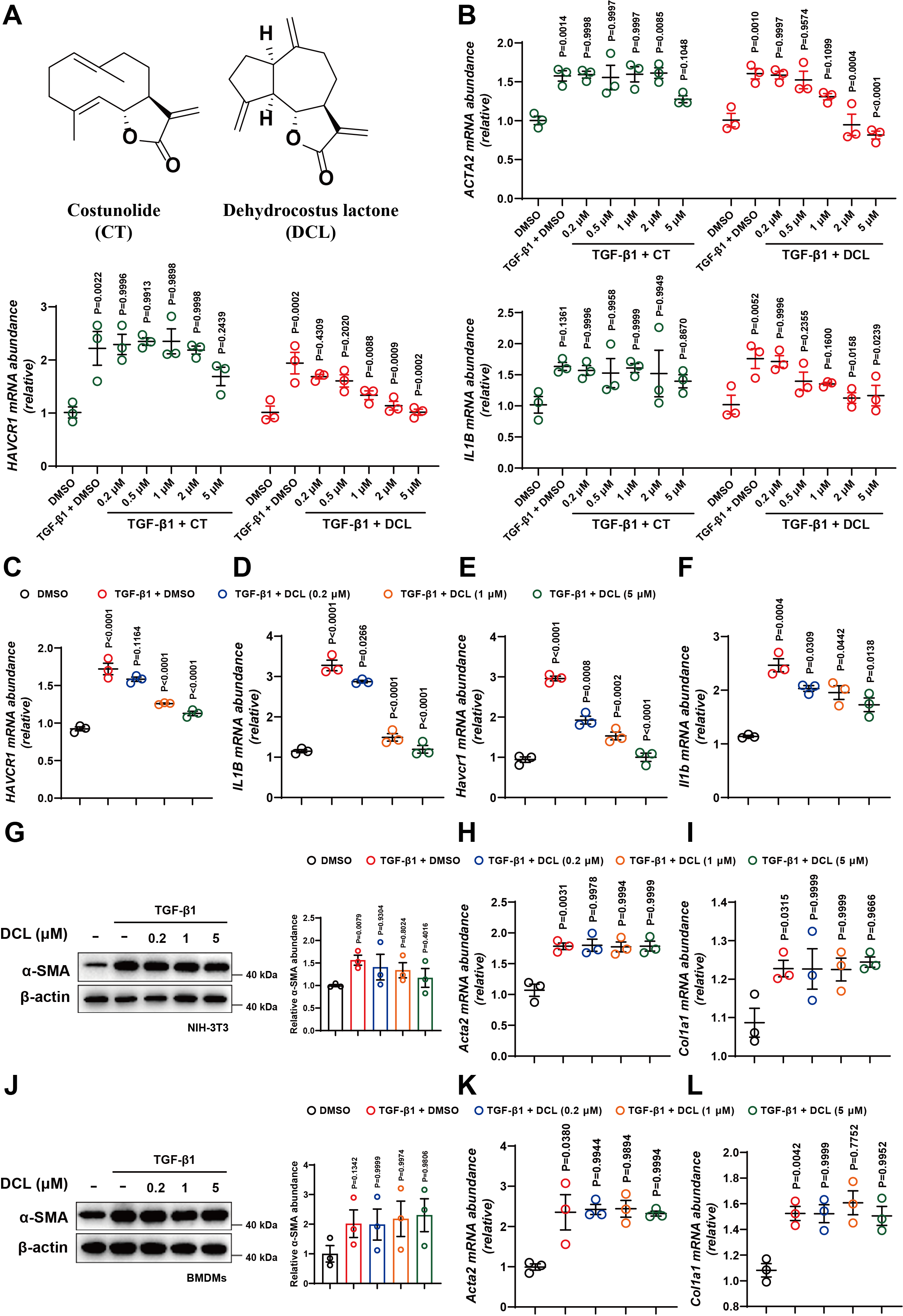
**

**Figure S2 DCL Alleviates TGF-β1-Induced Fibrosis in Renal Tubular Epithelial Cells.** **A,** Chemical structures of major bioactive sesquiterpene lactones from Aucklandiae Radix. **B**, qRT-PCR analysis of *ACTA2*, *HAVCR1* and *IL1B* mRNA expression in TGF-β1-induced HK-2 cells (n = 3 per group). **C-D**, qRT-PCR analysis of *HAVCR1* **(C)** and *IL1B* **(D)** mRNA expression in TGF-β1-induced HK-2 cells. **E-F**, qRT-PCR analysis of *Havcr1* **(E)** and *Il1b* **(F)** mRNA expression in TGF-β1-induced TCMK-1 cells (n = 3 per group). **G**, Representative western blot for α-SMA in TGF-β1-stimulated NIH-3T3 cells treated with indicated concentrations of DCL for 24 h (n = 3 per group). **H-I**, Effects of DCL on the expression profiles of *Acta2* (**H**) and *Col1a1* (**I**) genes in NIH-3T3 cells stimulated with TGF-β1. qRT–PCR assay was carried out in cells treated with indicated concentrations of DCL for 24 h (n = 3 per group). **J**, Representative western blot for α-SMA in TGF-β1-stimulated BMDM cells treated with indicated concentrations of DCL for 24 h (n = 3 per group). **K-L**, Effects of DCL on the expression profiles of *Acta2* (**K**) and *Col1a1* (**L**) genes in BMDM cells stimulated with TGF-β1. qRT–PCR assay was carried out in cells treated with indicated concentrations of DCL for 24 h (n = 3 per group). All statistic data were presented as mean ± SEM and statistical differences were determined by one-way ANOVA.

**
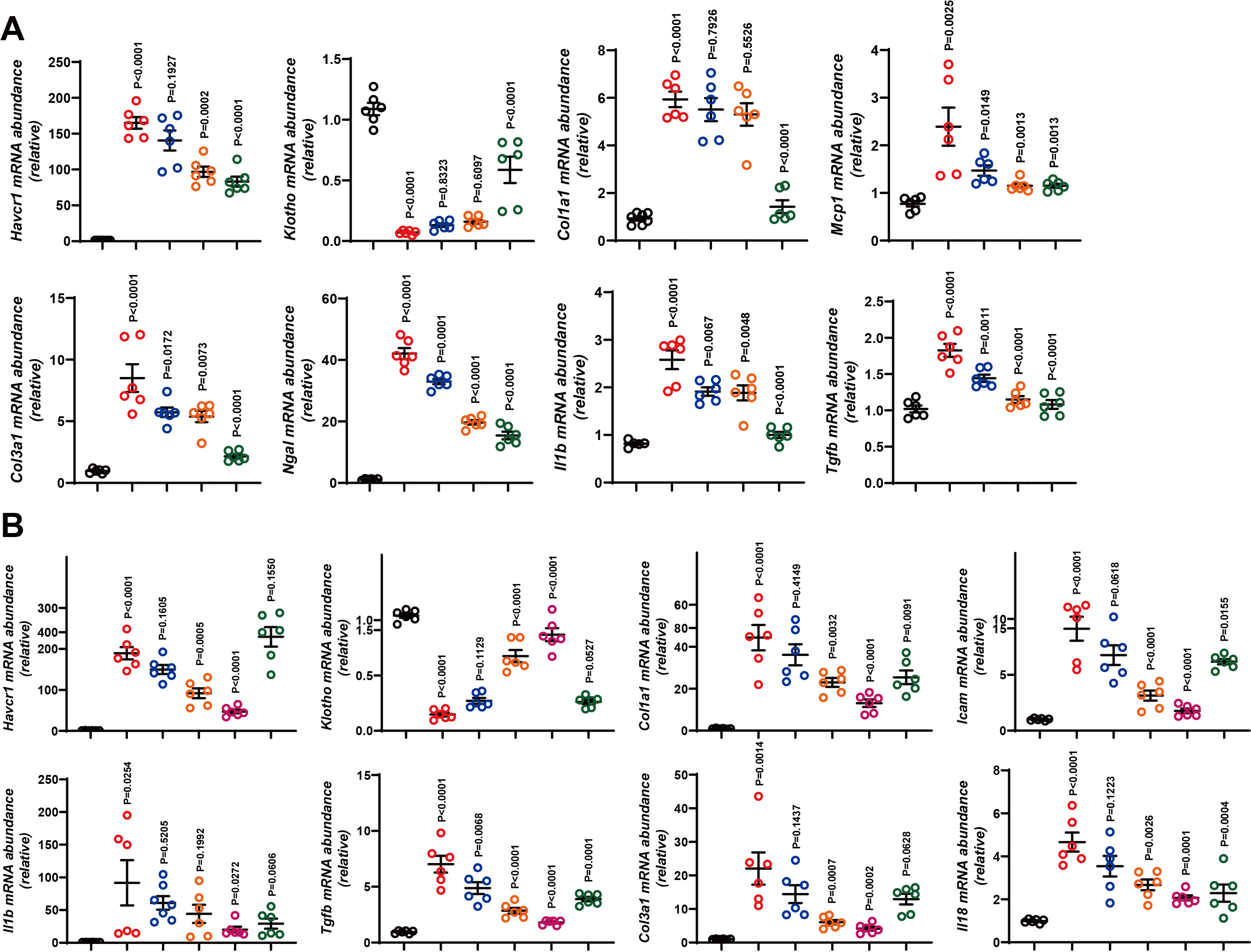
**

**Figure S3** **DCL Alleviates UUO-induced Renal Fibrosis *in vivo*. A**, qRT-PCR analysis of *Havcr1*, *Klotho*, *Col1a1*, *Mcp1*, *Il1b*, *Tgfb*, *Col3a1* and *Ngal* mRNA expression in mice renal tissue induced by Unilateral Ureteral Obstruction (UUO) (n = 6 per group). **B**, qRT-PCR analysis of *Havcr1*, *Klotho*, *Col1a1*, *Icam*, *Il1b*, *Tgfb*, *Col3a1* and *Il18* mRNA expression in mice renal tissue induced by UUO (n = 6 per group). All statistic data were presented as mean ± SEM and statistical differences were determined by one-way ANOVA.

**
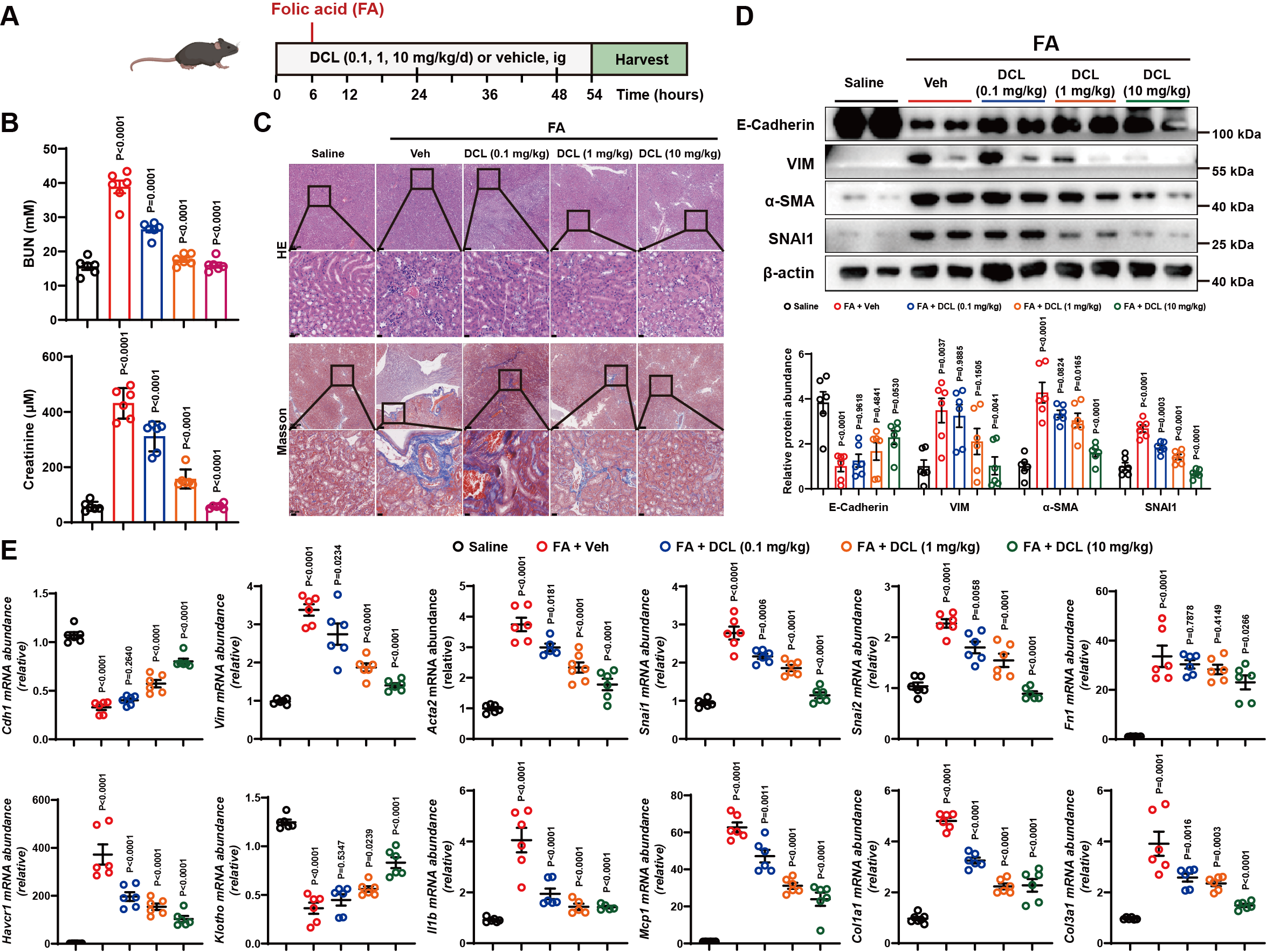
**

**Figure S4 DCL Alleviates FA-induced Renal Injury and Fibrosis *in vivo*. A**, Schematic overview of the experimental design of the FA-induced fibrosis model in mice. Mice were intraperitoneally injected with normal saline or FA once, and Veh, DCL (0.1, 1, and 10 mg/kg) were administered daily starting 6 h before FA. **B**, Creatinine and blood urea nitrogen in serum measured by ELISA (n = 6 per group). **C**, Representative images of mouse renal tissues stained with HE and Masson in indicated groups. Scale bar, 20 μm. **D**, Kidney homogenate samples were analyzed by western blotting (n = 6 per group) to quantify the protein levels of E-Cadherin, VIM, α-SMA and SNAI1. **E**, Kidney homogenate samples were analyzed by qRT-PCR (n = 6 per group) to quantify the gene levels of EMT marker, ﬁbrogenic and inflammatory factors.

**
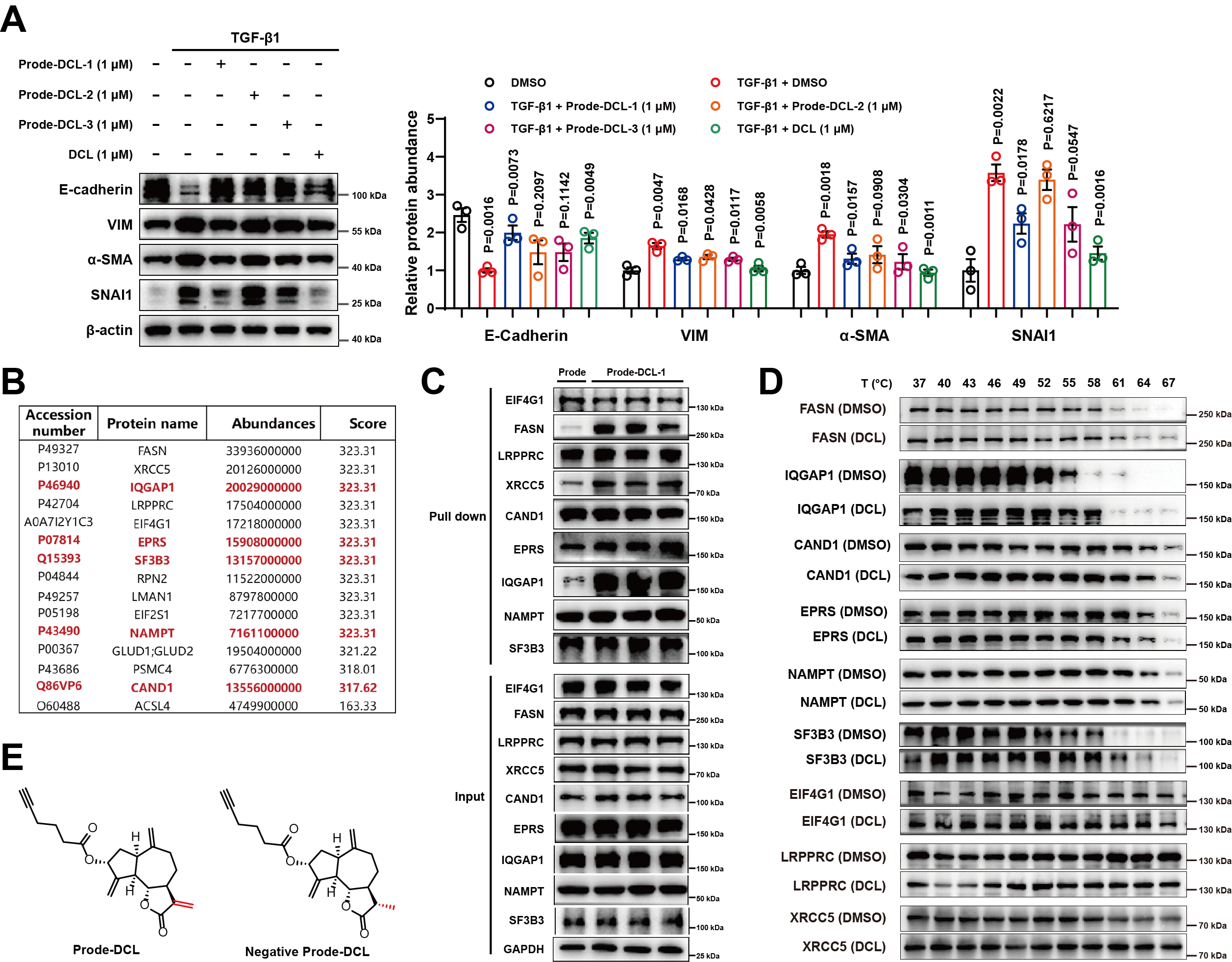
**

**Figure S5 DCL directly Targets IQGAP1. A**, Representative western blot for E-Cadherin, VIM, α-SMA and SNAI1 in TGF-β1-stimulated HK-2 cells treated different probe compounds (1 μM) for 24 h (n = 3 per group). All statistic data were presented as mean ± SEM and statistical differences were determined by one-way ANOVA. **B**, Top 15 DCL-binding proteins identified by ABPP assay, ranked by protein abundance and scoring metrics. **C**, TGF-β1-primed HK-2 cells were incubated with Prode or Prode-DCL-1 (10 μM) for 1 h, and the binding force of DCL to EIF4G1, FASN, LRPPRC, XRCC5, IQGAP1, CAND1, EPRS, NAMPT and SF3B3 was analyzed by pulldown. **D**, TGF-β1-primed HK-2 cells were incubated with DMSO or DCL (5 μM) for 1 h, and cellular thermal shift assays (CETSA) analyzed the thermal stabilization of EIF4G1, FASN, LRPPRC, XRCC5, IQGAP1, CAND1, EPRS, NAMPT and SF3B3 at different temperatures. **E**, Chemical structures of Prode-DCL and Negative Prode-DCL.

**
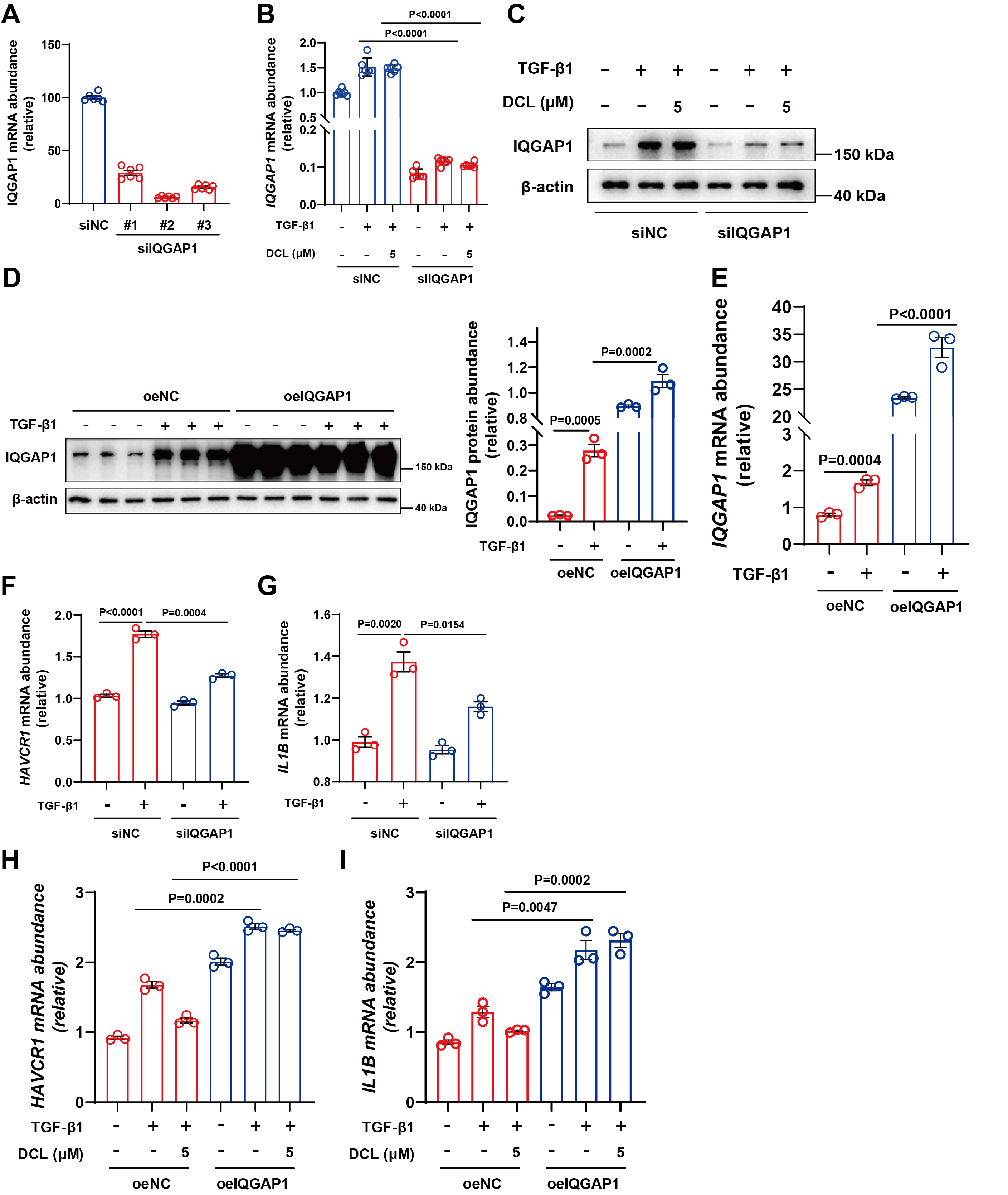
**

**Figure S6 DCL is Associated with the Progression of Renal Fibrosis. A**, mRNA expression level of *IQGAP1* (n = 6 per group). **B,** qRT-PCR analysis of *IQGAP1* mRNA expression in TGF-β1-induced HK-2 cells (n = 6 per group). **C**, Western blotting analysis of IQGAP1 protein expression in TGF-β1-induced HK-2 cells. **D**, Western blotting analysis of IQGAP1 protein expression in TGF-β1-induced HK-2 cells (n = 3 per group). **E,** qRT-PCR analysis of *IQGAP1* mRNA expression in TGF-β1-induced HK-2 cells (n = 3 per group). **F-G**, qRT-PCR analysis of *HAVCR1* **(F)** and *IL1B* **(G)** mRNA expression in TGF-β1-induced HK-2 cells (n = 3 per group). **H-I**, qRT-PCR analysis of *HAVCR1* **(H)** and *IL1B* **(I)** mRNA expression in TGF-β1-induced HK-2 cells (n = 3 per group). All statistic data were presented as mean ± SEM and statistical differences were determined by one-way ANOVA.

**
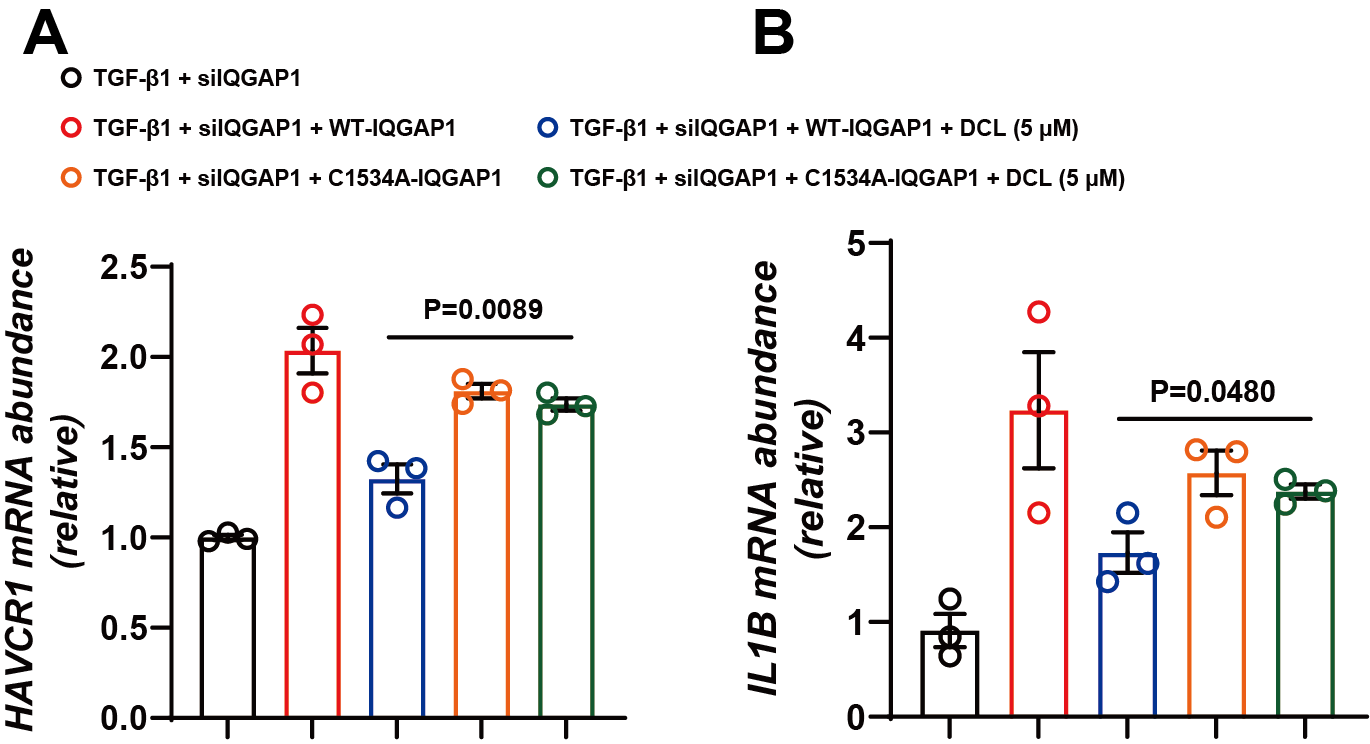
**

**Figure S7 Mapping the Binding Sites of DCL on IQGAP1. A-B**, qRT-PCR analysis of *HAVCR1* (**A**) and *IL1B* (**B**) mRNA expression in TGF-β1-induced HK-2 cells (n = 3 per group). All statistic data were presented as mean ± SEM and statistical differences were determined by one-way ANOVA.

**
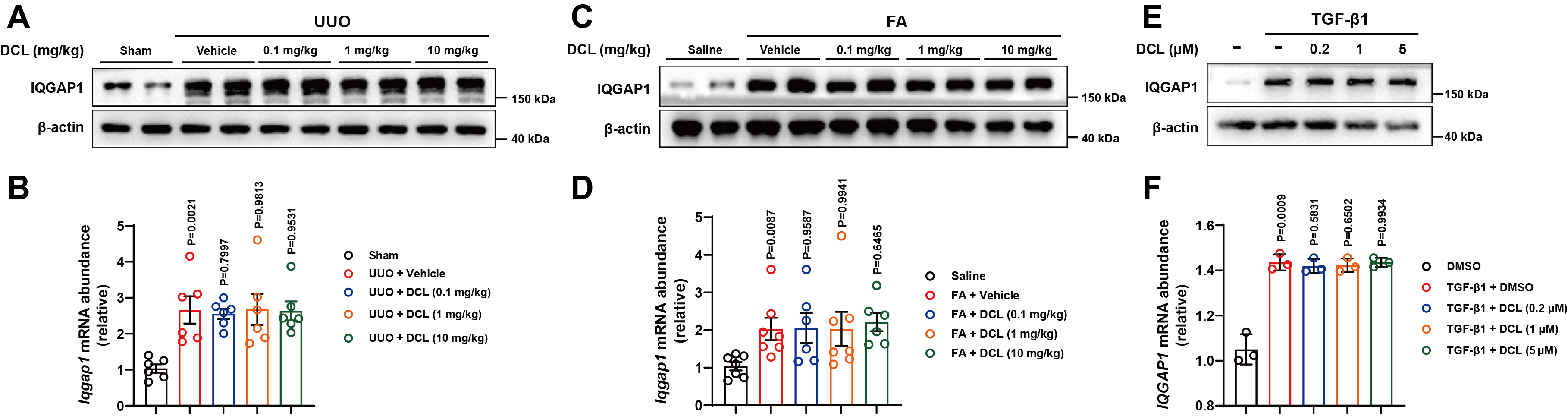
**

**Figure S8 DCL Did not Affect the Protein and mRNA Expression of IQGAP1 both *in vivo* and *in vitro*.** **A**, Western blotting analysis of IQGAP1 protein expression in mice renal tissue induced by UUO. **B**, qRT-PCR analysis of *IQGAP1* mRNA expression in mice renal tissue induced by UUO (n = 6 per group). **C**, Western blotting analysis of IQGAP1 protein expression in mice renal tissue induced by FA. **D**, qRT-PCR analysis of *Iqgap1* mRNA expression in mice renal tissue induced by FA (n = 6 per group). **E**, Western blotting analysis of IQGAP1 protein expression in TGF-β1-induced HK-2 cells. **F**, qRT-PCR analysis of *Iqgap1* mRNA expression in TGF-β1-induced HK-2 cells (n = 3 per group). All statistic data were presented as mean ± SEM and statistical differences were determined by one-way ANOVA.

**
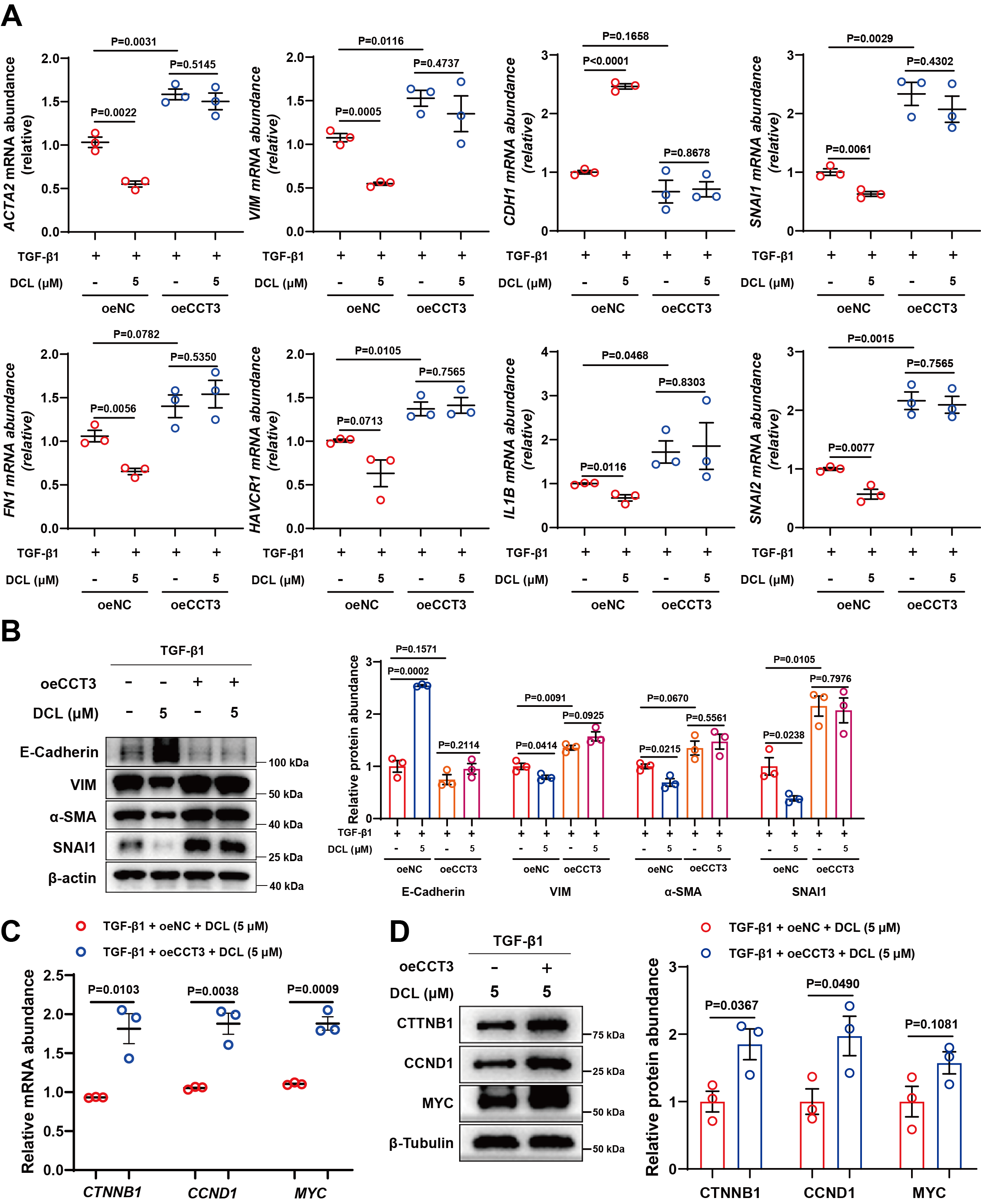
**

**Figure S9 DCL Blocked the Interaction between IQGAP1 and CCT3 to Inactivating Wnt Signaling Pathway.** **A**, qRT-PCR analysis of *ACTA2*, *VIM*, *CDH1*, *SNAI1*, *FN1*, *HAVCR1*, *IL1B* and *SNAI2* mRNA expression in TGF-β1-induced HK-2 cells (n = 3 per group). **B**, Western blotting analysis of E-Cadherin, VIM, α-SMA and SNAI1 protein expression in TGF-β1-induced HK-2 cells (n = 3 per group). **C**, qRT-PCR analysis of *CTNNB1*, *CCND1* and *MYC* mRNA expression in TGF-β1-induced HK-2 cells (n = 3 per group). **D**, Western blotting analysis of CTNNB1, CCND1 and MYC protein expression in TGF-β1-induced HK-2 cells (n = 3 per group). All statistic data were presented as mean ± SEM and statistical differences were determined by one-way ANOVA.

**
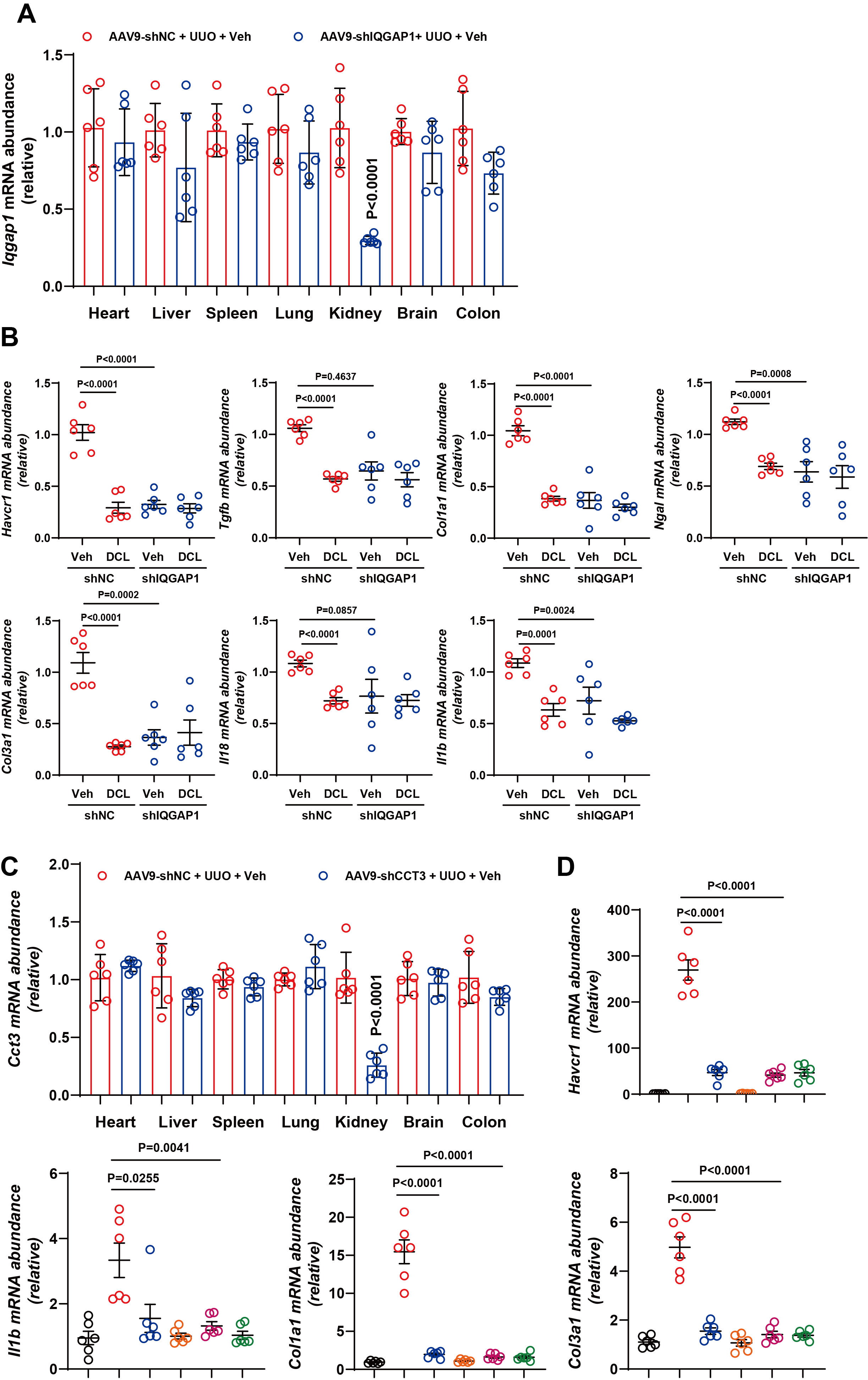
**

**Figure. S10 Specific IQGAP1 or CCT3 Deletion Inhibited Renal Fibrosis in Mice. A**, qRT-PCR analysis of *Iqgap1* mRNA expression in mice renal tissue induced by UUO (n = 6 per group). **B**, qRT-PCR analysis of *Havcr1*, *Tgfb*, *Col1a1*, *Ngal*, *Col3a1*, *Il18* and *Il1b* mRNA expression in mice renal tissue induced by UUO (n = 6 per group). **C**, qRT-PCR analysis of *Cct3* mRNA expression in mice renal tissue induced by UUO (n = 6 per group). **D**, qRT-PCR analysis of *Havcr1, Il1b, Col1a1* and *Col3a1* mRNA expression in mice renal tissue induced by UUO (n = 6 per group). All statistic data were presented as mean ± SEM and statistical differences were determined by one-way ANOVA.

**Table S1.** **Detailed** **symptom names**

| **Symptom** | edema, fatigue, abdominal pain, abdominal distention, dyspnea, cough, poor appetite, vomiting, pruritus, limb pain, headache, dizziness, gastric discomfort, weight loss, palpitations, chest pain, hyperhidrosis, polyuria, restlessness, joint stiffness, joint pain, sweating, limited mobility, shortness of breat, emotional lability, general body pain, lower limb weakness, hematuria, anorexia, irritability |
| --- | --- |

**Table S2. a detailed list of the 334 proteins**

See the Supporting Information Table S2 for details.

**Table S3. Detailed information of non-fibrotic kidney and fibrotic kidney**

|  | **Gender** | **Age** | **Creatinine** | **Blood Urea Nitrogen** | **eGFR** | **Stage of CKD** | **Diagnosis** |
| --- | --- | --- | --- | --- | --- | --- | --- |
| Non-fibrotic Kidney | Male | 62 | 74.7 | 6.9 | 98.6 | / | Renal cell carcinoma, uremia |
|  | Female | 49 | 49.9 | 2.31 | 97.4 | / | Renal cell carcinoma, uremia |
| Fibrotic Kidney | Female | 48 | 767.3 | 14.27 | 5.2 | Stage 5 CKD | Renal cell carcinoma |
|  | Male | 66 | 117.4 | 8.59 | 59.4 | Stage 3 CKD | Renal cell carcinoma, chronic kidney disease |

**Scheme S1. Establishment procedures of the multimodal AI-driven TCM-SPred**

This study proposes a Multimodal AI-driven TCM-Symptom Prediction model (MTC-SPred), which integrates herb-symptom associations (effective/ineffective), TCM symptom targets, herb targets, and protein-protein interaction (PPI) networks to predict therapeutic effects. The workflow comprises four stages: data preprocessing, feature extraction, model construction, and training/validation. Key methodological innovations are described below. The methodological pipeline of the MTC-SPred model is illustrated in **Figure 1**.

(1) Data Preprocessing

We collected data on 369 Traditional Chinese Medicine (TCM) herbs, 73 symptoms, and 2,010 herb-symptom therapeutic relationships, including both effective and ineffective interactions, from the herb-symptom dataset provided by Xiao Gan et al^45^. In addition, target protein information for the herbs and symptoms, totaling approximately 250,000 entries, was obtained from HIT2.0^46^ and herb-symptom dataset. The PPI network data were retrieved from the STRING database^47^. For each herb-symptom pair, we computed bidirectional PPI features to quantify target interactions: For herb targets $H=\{h_{1},h_{2},\ldots,h_{m}\}$ and symptom targets $S=\{s_{1},s_{2},\ldots,s_{n}\}$: $f_{h}\left( i \right)=\frac{1}{\left| S \right|}\sum_{s_{j}\in S} PPI\left( h_{i},s_{j} \right)$. Similarly, the feature for $s_{j}$: $f_{s}\left( j \right)=\frac{1}{\left| H \right|}\sum_{h_{i}\in H} \text{PPI}\left( h_{i},s_{j} \right)$. These features encode the interaction strength between herb and symptom targets, forming the input for subsequent modules.

(2) Feature Extraction and Embedding

To capture the latent interplay among herbs, symptoms and targets, we first re-expressed the heterogeneous relational knowledge as natural-language sentences that preserve rich biomedical semantics, then fed the entire corpus to Word2Vec, which distilled it into 128-dimensional dense vectors encoding both therapeutic efficacy and mechanistic connectivity. Specifically, we leveraged the four curated datasets (D1-D4) to assemble a domain-specific corpus in the following manner:

Therapeutic sentences:

From D1 (effective pairs) we generated bidirectional clauses, e.g. [symptom, "commonly used", herb, "for treatment"] and From D2 (ineffective pairs) we generated negative clauses, e.g. [symptom, "not suitable", herb, "for treatment"].

Mechanistic sentences:

D3 supplies herb → target links; each herb was described as [herb, "targets include", target1, target2, …]. D4 links symptoms to pathogenic genes, forming sentences such as [symptom, "associated genes include", gene1, gene2, …].

With the composite sentence set in place, we trained a skip-gram Word2Vec^48^ model to translate these textual cues into shared vector space. The key hyper-parameters were chosen to balance semantic resolution and computational efficiency: 128-dimensional embeddings, an 8-word context window, a minimum frequency threshold of 2 to suppress noise, five negative samples per positive example, and 200 training epochs with an initial learning rate of 0.025 that decayed smoothly to 0.0001. Concurrently, for each herb-symptom pair, we computed bidirectional PPI features to quantify target interactions: For herb targets $H=\{h_{1},h_{2},\ldots,h_{m}\}$ and symptom targets $S=\{s_{1},s_{2},\ldots,s_{n}\}$: $f_{h}\left( i \right)=\frac{1}{\left| S \right|}\sum_{s_{j}\in S} PPI\left( h_{i},s_{j} \right)$. Similarly, the feature for $s_{j}$: $f_{s}\left( j \right)=\frac{1}{\left| H \right|}\sum_{h_{i}\in H} \text{PPI}\left( h_{i},s_{j} \right)$. These bidirectional PPI scores were converted to 1-dimensional features, complementing the semantic embeddings to form a comprehensive representation of herb-symptom interactions.

(3) MTC-SPred Model

The MTC-SPred model integrates four key components: 1) Main Feature Processor, 2) Bidirectional Target Encoders, 3) Cross-Modal Attention, and 4) Classifier.

1) Main Feature Processor: Concatenates 128-dimensional herb and symptom embeddings into a 256-dimensional vector. Processes through linear layer with layer normalization and ReLU activation: $z_{0}=\text{ReLU}\left( \text{LayerNorm}\left( W_{f}\left[ e_{h}\parallel e_{s} \right]+b_{f} \right) \right).$Then, pass through a linear layer to output the 128-dimensional main feature vector.

2) Bidirectional Target Encoders: Processes variable-length target sequences using bidirectional LSTMs. Input features combine 128D symbol embeddings and 1D PPI scores: $input=\left[ e_{symbol}\parallel\phi\left( g \right) \right]$. The BiLSTM processes variable-length sequences with 2 layers and 64 hidden units per direction, outputting 128D representations.

3) Cross-Modal Attention: Integrates four feature types into 512D matrix: 1) Processed main features ($z_{0}$); 2) Herb target encodings ($H_{h}$); 3) Symptom context vector ($c_{s}=\text{mean}\left( H_{s} \right)$);4) Interaction features ($H_{h}\odot c_{s}$). A multiheaded attention mechanism aligns herb and symptom features: $\text{Attention}\left( Q,K,V \right)=\text{softmax}\left( \frac{QK^{\top}}{\sqrt{d_{k}}} \right)V$, where $d_{k}=128$ per head captures diverse interaction patterns. Four attention heads ($h$=4) capture diverse interaction patterns.

4) Classifier: 1) Global average pooling: $p=\frac{1}{L}\sum_{i=1}^{L} X_{i}^{'}$. 2) Two fully-connected layers with ReLU activations. 3) Sigmoid activation for probability prediction: $\hat{y}=\sigma\left( W_{2}\cdot\text{ReLU}\left( W_{1}\cdot p+b_{1} \right)+b_{2} \right)$.

(4) Training

The dataset was partitioned into training (70%), validation (15%), and test (15%) sets to ensure rigorous evaluation. We used the AdamW optimizer with Focal Loss to address class imbalance. The learning rate schedule combined 5-epoch linear warmup and 45-epoch cosine annealing, with training conducted in batches of 8 using dynamic padding for variable-length sequences, and early stopping triggered after 10 epochs without validation AUC improvement. Comprehensive training specifications are provided in **Table S4**.

**Table S4.**

| **Parameter name** | **Value** |
| --- | --- |
| Optimizer | AdamW |
| Base Learning Rate | 5$e^{-5}$ |
| Weight Decay | 1$e^{-3}$ |
| Loss Function | Focal Loss |
| Batch Size | 8 |
| Warmup Epochs | 5 |
| Cosine Annealing Epochs | 45 |
| Early Stopping | 10 epochs |

**Table S5. Primers for qPCR**

| **Gene** | **Forward Sequence (5’-3’)** | **Reverse Sequence (5’-3’)** |
| --- | --- | --- |
| Mouse *Gapdh* | GTCAAGGCTGAGAACGGGAA | AAATGAGCCCCAGCCTTCTC |
| Mouse *Acta2* | CTTCGTGACTACTGCCGAGC | TGCATCCTGTCAGCAATGCCT |
| Mouse *Vim* | GAGCTATGTGACCACGTCCA | CCGGGGGATGAGGAATAGAG |
| Mouse *Snai1* | CCGGAAGCCCAACTATAGCG | TGGGGTACCAGGAGAGAGTC |
| Mouse *Snai2* | TACAGCGAACTGGACACACA | GTAAAGGAGAGTGGAGTGGAGC |
| Mouse *Cdh1* | CTGTGAAGGGACGGTCAACA | ATCAGAATCAGCAGGGCGAG |
| Mouse *Il1b* | ATCTCGCAGCAGCACATCAA | ATGGGAACGTCACACACCAG |
| Mouse *Fn1* | GAAGTCGCAAGGAAACAAGC | GCCACCATAAGTCTGGGTCA |
| Mouse *Col1a1* | CTGACGCATGGCCAAGAAGA | CGTGCCATTGTGGCAGATAC |
| Mouse *Col3a1* | TGACTGTCCCACGTAAGCAC | GGAGGGCCATAGCTGAACTG |
| Mouse *Icam* | CTGAAAGATGAGCTCGAGAGTG | AAACGAATACACGGTGATGGTA |
| Mouse *Il18* | ATCTCGCAGCAGCACATCAA | ATGGGAACGTCACACACCAG |
| *Mouse Tgfb* | CCAGATCCTGTCCAAACTAAGG | CTCTTTAGCATAGTAGTCCGCT |
| Mouse *Ngal* | ATGTCACCTCCATCCTGGTCAG | GCCACTTGCACATTGTAGCTCTG |
| Mouse *Mcp1* | GCTACAAGAGGATCACCAGCAG | GTCTGGACCCATTCCTTCTTGG |
| Mouse *Havcr1* | CTGGAATGGCACTGTGACATCC | GCAGATGCCAACATAGAAGCCC |
| Mouse *Iqgap1* | ACCAGAGTGACCTTGCTGAAGC | TTGCGTCTCCAGGTTGTGGTAG |
| Mouse *Myc* | TCGCTGCTGTCCTCCGAGTCC | GGTTTGCCTCTTCTCCACAGAC |
| Mouse *Ctnnb1* | GTTCGCCTTCATTATGGACTGCC | ATAGCACCCTGTTCCCGCAAAG |
| Mouse *Ccnd1* | GCAGAAGGAGATTGTGCCATCC | AGGAAGCGGTCCAGGTAGTTCA |
| Human *GAPDH* | GTCAAGGCTGAGAACGGGAA | AAATGAGCCCCAGCCTTCTC |
| Human *ACTA2* | CTATGAGGGCTATGCCTTGCC | GCTCAGCAGTAGTAACGAAGGA |
| Human *IL1B* | GCAGAAGTACCTGAGCTCGC | CATGGCCACAACAACTGACG |
| Human *HAVCR1* | CTTCACCTCAGCCAGCAGAAAC | GCCATCTGAAGACTCTGTCACG |
| Human *VIM* | GGACCAGCTAACCAACGACA | AAGGTCAAGACGTGCCAGAG |
| Human *SNAI1* | CCTCGCTGCCAATGCTCATCTG | GCTCTGCCACCCTGGGACTC |
| Human *SNAI2* | CGAACTGGACACACATACAGTG | CTGAGGATCTCTGGTTGTGGT |
| Human *CDH1* | CGAGAGCTACACGTTCACGG | GGGTGTCGAGGGAAAAATAGG |
| Human *FN1* | CCGGGACTCAATCCAAATGC | TCCGTAGGTTGGTTCAAGCC |
| Human-*IL1B* | GCAGAAGTACCTGAGCTCGC | CATGGCCACAACAACTGACG |
| Human *IQGAP1* | CCGTGGATACTTAGTTCGACAGG | AGCGCAGGTAAGCTAACCGATC |
| Human *MYC* | CCTGGTGCTCCATGAGGAGAC | CAGACTCTGACCTTTTGCCAGG |
| Human *CTNNB1* | CACAAGCAGAGTGCTGAAGGTG | GATTCCTGAGAGTCCAAAGACAG |
| Human *CCND1* | TCTACACCGACAACTCCATCCG | TCTGGCATTTTGGAGAGGAAGTG |

**Table S6. The primary antibodies used were as follows:**

| **Antibody** | **Brand entity** | **Product Code** | **Usage and dilution ratio** |
| --- | --- | --- | --- |
| anti-E-Cadherin | Proteintech | 20874-1-AP | 1:1000 for WB;  1:400 for IF;  1:400 for IHC |
| anti-Vimentin | Proteintech | 10366-1-AP | 1:1000 for WB;  1:400 for IF;  1:400 for IHC |
| anti-Snail | Proteintech | 13099-1-AP | 1:1000 for WB; |
| anti-α-smooth muscle actin | Proteintech | 14395-1-AP | 1:1000 for WB; |
| anti-β-Tubulin | Abmart | M20005S | 1:1000 for WB |
| anti-β-actin | Abmart | T40104S | 1:1000 for WB |
| anti-IQGAP1 | Proteintech | 22167-1-AP | 1:1000 for IP;  1:100 for PLA;  1:200 for IHC;  1:8000 for IP;  1:200 for IF |
| anti-IQGAP1 | Santa Cruz | sc-374307 | 1:100 for IF;  1:100 for IHC |
| anti-CAND1 | Abcam | AB181216 | 1:1000 for WB |
| anti-SF3B3 | ABclonal | A9624 | 1:1000 for WB |
| anti-EPRS | Proteintech | 25307-1-AP | 1:1000 for WB |
| anti-C Myc | Abways | CY5150 | 1:1000 for WB |
| anti-Wnt1 | Abways | AY0229 | 1:1000 for WB |
| anti-Cyclin D1 | Abways | CY5404 | 1:1000 for WB |
| anti-Beta Catenin | Abways | CY3523 | 1:1000 for WB |
| anti-CCT3 | Proteintech | 10571-1-AP | 1:800 for WB;  1:1000 for IP |
| anti-HA-tag | Zenbio | 301113 | 1:1000 for WB;  1:100 for IP |
| anti-Flag-tag | Zenbio | R24091 | 1:1000 for WB;  1:100 for IP |
| Anti-rabbit IgG, HRP-linked antibody | Abbkine | A21020 | 1:10000 for WB |
| anti-mouse IgG, HRP-linked antibody | Abbkine | A21010 | 1:10000 for WB |
| Alexa Fluor 488 goat anti-rabbit IgG | Abbkine | A23220 | 1:500 for IF |
| Alexa Fluor 488 goat anti-mouse IgG | Abbkine | A23210 | 1:500 for IF |
| Alexa Fluor Cy3 goat anti-mouse IgG | Abbkine | A22210 | 1:500 for IF |
| HRP-Streptavidin | Abbkine | A0305 | 1:500 for IF |
| EIF4G1 Polyclonal antibody | Proteintech | 15704-1-AP | 1:2000 for WB |
| FASN Polyclonal antibody | Proteintech | 10624-2-AP | 1:5000 for WB |
| XRCC5 Polyclonal antibody | Proteintech | 16389-1-AP | 1:2000 for WB |
| LRPPRC Polyclonal antibody | Proteintech | 21175-1-AP | 1:5000 for WB |

**Scheme S2.** **Data of chemical**

All chemical reagents (Macklin, Aladdin, Titan, Bide Pharmatech) and solvents were used without further purification. Purification was performed using 200-300 mesh silica gel column chromatography (Qingdao Marine Chemicals Co., Ltd.), and solvents were removed under reduced pressure using a BUCHI R-300 rotary evaporator. Mass spectrometric data were acquired on an Agilent 6120 single quadrupole mass spectrometer equipped with electrospray ionization (ESI). Nuclear magnetic resonance (NMR) spectra were recorded on a Bruker Avance III 500 MHz superconducting NMR spectrometer with tetramethylsilane (TMS) as the internal standard: ^1^H NMR (500 MHz) and ^13^C NMR (126 MHz) chemical shifts (δ) were reported in parts per million (ppm), with coupling constants (J) expressed in Hertz (Hz). Compound purity was verified using a Waters E2695 high-performance liquid chromatography (HPLC) system equipped with a Hedera ODS-2 C^18^ column (4.6 mm × 250 mm, 5 μm), employing a methanol-water gradient elution (55/45 to 95/5, within 30 min) at a flow rate of 0.8 mL/min with detection at 254 nm. All target compounds were confirmed to exhibit ≥ 95% purity as determined by peak area normalization.

**Synthetic schemes and methods of compounds**

DCL were extracted from Aucklandiae Radix.

DCL: ^1^H NMR (500 MHz, CDCl3) δ 6.20 (d, J = 3.5 Hz, 1H), 5.48 (d, J = 3.1 Hz, 1H), 5.25 (d, 1H), 5.05 (d, 1H), 4.88 (s, 1H), 4.80 (s, 1H), 3.95 (t, J = 9.3 Hz, 1H), 2.95-2.88 (m, 1H), 2.88-2.82 (m, 2H), 2.53 (d, J = 8.6 Hz, 1H), 2.51-2.44 (m, 2H), 2.27-2.20 (m, 1H), 2.19-2.11 (m, 1H), 1.94 (dt, J = 13.2, 8.5 Hz, 1H), 1.90-1.82 (m, 1H), 1.46-1.36 (m, 1H).^13^C NMR (126 MHz, CDCl3) δ 170.32 , 151.34 , 149.33 , 139.87 , 120.24 , 112.70 , 109.68 , 85.32 , 52.13 , 47.72 , 45.23 , 36.36 , 32.71 , 31.04 , 30.41 . LC-MS: calcd for C15H18O2 [M + H]^+^ = 231.2; found 231.2. HPLC purity: 100.00% (t_R_ = 26.190 min).

Synthetic schemes for compound Int1


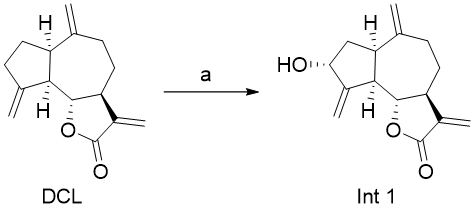


Reagents and conditions: (a) SeO2, TBHP, AcOH, DCM, rt, 24 h.

DCL (920 mg, 4 mmol) was dissolved in anhydrous dichloromethane (DCM, 1 mL), followed by sequential addition of selenium dioxide (SeO_2_, 7.4 mg, 0.08 mmol), tert-butyl hydroperoxide (TBHP, 5-6 M in decane, 4.8 mmol), and acetic acid (AcOH, 24 mg, 0.4 mmol). The reaction mixture was stirred at room temperature for 24 h, after which a saturated aqueous sodium thiosulfate (Na_2_S_2_O_3_) solution was added. The mixture was stirred for an additional 10 min and then extracted with ethyl acetate (EtOAc, 3 × 10 mL). The combined organic layers were washed with water (3 × 4 mL) and saturated brine (6 mL), dried over anhydrous sodium sulfate (Na_2_SO_4_), and concentrated under reduced pressure. The crude product was purified by silica gel column chromatography (200-300 mesh) to afford compound Int1 (511.68 mg, 52% yield).

Compound Int1: ^1^H NMR (500 MHz, CDCl3) δ 6.23 (d, J = 3.5 Hz, 1H), 5.51 (d, J = 3.1 Hz, 1H), 5.50 (s, 1H), 5.37 (s, 1H), 4.94 (s, 1H), 4.79 (s, 1H), 4.70 (t, J = 6.2 Hz, 1H), 3.91 (t, J = 9.2 Hz, 1H), 3.11 (t, J = 6.1 Hz, 2H), 2.90-2.82 (m, 1H), 2.53 (dt, J = 13.0, 4.8 Hz, 1H), 2.28-2.17 (m, 2H), 2.16-2.07 (m, 1H), 1.92-1.84 (m, 1H), 1.47-1.35 (m, 1H).13C NMR (126 MHz, CDCl3) δ 170.17, 154.27, 148.63, 139.59, 120.58, 113.43, 113.29, 85.03, 74.73, 49.75, 45.74, 44.37, 40.03, 36.84, 31.16. LC-MS: calcd for C_15_H_18_O_3_ [M + H]^+^ = 247.2; found 247.2. HPLC purity: 100.00% (t_R_ = 21.409 min).

Synthetic schemes for compound Prode-DCL-1

Reagents and conditions: 5-hexynoyl chloride, pyridine, DMAP, 0 °C to rt, 12 h.

Oxalyl chloride (152.4 mg, 1.2 mmol) was added to a solution of 5-hexynoic acid (44.9 mg, 0.4 mmol) in anhydrous dichloromethane (DCM, 2 mL), followed by the addition of one drop of dimethylformamide (DMF). The mixture was stirred for 1.5 h, after which excess oxalyl chloride was removed under reduced pressure. The residue was re-dissolved in anhydrous DCM (5 mL) at 0 °C, and pyridine (94.8 mg, 1.2 mmol), 4-dimethylaminopyridine (DMAP, catalytic amount), and compound **Int1** (88.61 mg, 0.36 mmol) were sequentially added. The reaction was stirred at room temperature for 12 h, then quenched with ethyl acetate (30 mL). The organic phase was washed sequentially with saturated aqueous citric acid solution (3 × 10 mL), saturated aqueous sodium bicarbonate (NaHCO_3_, 3 × 10 mL), water (3 × 10 mL), and saturated brine (10 mL), dried over anhydrous sodium sulfate (Na_2_SO_4_), and concentrated in vacuo. The crude product was purified by silica gel column chromatography (200-300 mesh) to afford **Prode-DCL-1** (55.05 mg, 45% yield).

Prode-DCL-1: ^1^H NMR (500 MHz, CDCl3) δ 6.23 (d, J = 3.5 Hz, 1H), 5.73-5.66 (m, 1H), 5.55 (t, J = 1.9 Hz, 1H), 5.51 (d, J = 3.1 Hz, 1H), 5.39 (t, J = 1.9 Hz, 1H), 4.95 (d, 1H), 4.81 (d, 1H), 3.91 (t, J = 9.0 Hz, 1H), 3.14-3.01 (m, 2H), 2.92-2.82 (m, 1H), 2.56-2.50 (m, 1H), 2.48-2.43 (m, 2H), 2.26 (dd, J = 6.8, 2.5 Hz, 2H), 2.17-2.09 (m, 1H), 1.97 (t, J = 2.7 Hz, 1H), 1.95-1.88 (m, 1H), 1.87-1.83 (m, 2H), 1.45-1.36 (m, 1H), 1.30 (d, J = 24.2 Hz, 1H), 1.24 (d, J = 7.0 Hz, 1H).^13^C NMR (126 MHz, CDCl3) δ 172.85, 170.05, 149.21, 148.15, 139.40, 120.67, 115.73, 113.62, 85.11, 83.33, 76.20, 69.31, 49.98, 45.40, 44.81, 37.46, 36.86, 33.30, 31.16, 23.76, 17.97. LC-MS: calcd for C_21_H_24_O_4_ [M + H]^+^ = 341.2; found 341.2. HPLC purity: 100.00% (t_R_ = 25.949 min).

Synthetic schemes for compound Prode-DCL-2

Reagents and conditions: 7-Octynoic acid, pyridine, DMAP, 0 °C to rt, 12 h.

Oxalyl chloride (152.4 mg, 1.2 mmol) was added to a solution of 7-octynoic acid (56.07 mg, 0.4 mmol) in anhydrous dichloromethane (DCM, 2 mL), followed by the addition of one drop of dimethylformamide (DMF). Subsequent operations, including reaction workup, purification, and analytical characterization, were performed identically to the synthesis of Prode-DCL-1 as previously described. The target compound Prode-DCL-2 was obtained as a purified product (39.77 mg, 30% yield) via silica gel column chromatography (200-300 mesh).

Prode-DCL-2: ^1^H NMR (500 MHz, CDCl3) δ 6.23 (d, J = 3.5 Hz, 1H), 5.69 (t, J = 6.2 Hz, 1H), 5.55 (s, 1H), 5.51 (d, J = 3.1 Hz, 1H), 5.39 (s, 1H), 4.95 (s, 1H), 4.82 (s, 1H), 3.91 (t, J = 9.1 Hz, 1H), 3.14-3.04 (m, 2H), 2.92-2.83 (m, 1H), 2.53 (dt, J = 13.1, 4.7 Hz, 1H), 2.33 (dd, J = 9.2, 5.7 Hz, 2H), 2.30-2.22 (m, 2H), 2.19 (td, J = 7.0, 2.6 Hz, 2H), 2.12 (dt, J = 12.8, 5.7 Hz, 1H), 1.93 (t, J = 2.6 Hz, 1H), 1.92-1.87 (m, 1H), 1.65 (dt, J = 15.1, 7.5 Hz, 2H), 1.55 (p, J = 7.0 Hz, 2H), 1.49-1.35 (m, 3H). ^13^C NMR (126 MHz, CDCl3) δ 173.42, 170.06, 149.30, 148.19, 139.44, 120.66, 115.65, 113.63, 85.15, 84.45, 76.05, 68.50, 50.03, 45.43, 44.84, 37.51, 36.90, 34.54, 31.19, 28.29, 28.22, 24.62, 18.39. LC-MS: calcd for C_23_H_28_O_4_ [M + H]^+^ = 369.2; found 369.2. HPLC purity: 100.00% (t_R_ = 27.228 min).

Synthetic schemes for compound Prode-DCL-3

Reagents and conditions: D-Biotin, pyridine, DMAP, 0 °C to rt, 12 h.

Oxalyl chloride (152.4 mg, 1.2 mmol) was added to a solution of biotin (97.68 mg, 0.4 mmol) in anhydrous dichloromethane (DCM, 2 mL), followed by the addition of one drop of dimethylformamide (DMF). Subsequent reaction workup, purification, and analytical procedures were performed in accordance with the synthetic protocol for Prode-DCL-1 as previously detailed. The title compound Prode-DCL-3 was isolated via silica gel column chromatography (200–300 mesh), yielding 73.09 mg (43% yield) of purified product.

Prode-DCL-3: ^1^H NMR (500 MHz, DMSO-d6) δ 6.43 (s, 1H), 6.35 (s, 1H), 6.04 (d, J = 3.5 Hz, 1H), 5.64 (d, J = 3.1 Hz, 1H), 5.62-5.57 (m, 1H), 5.32 (d, J = 2.1 Hz, 1H), 5.28 (s, 1H), 4.91 (s, 1H), 4.72 (s, 1H), 4.29 (dd, J = 7.7, 5.1 Hz, 1H), 4.14-4.10 (m, 1H), 3.91 (t, J = 8.9 Hz, 1H), 3.14-3.05 (m, 3H), 3.04-2.95 (m, 1H), 2.81 (dd, J = 12.4, 5.1 Hz, 1H), 2.56 (d, J = 12.4 Hz, 1H), 2.44 (dt, J = 12.9, 4.7 Hz, 1H), 2.31 (t, J = 7.4 Hz, 2H), 2.27-2.19 (m, 2H), 2.14-2.06 (m, 1H), 1.87-1.79 (m, 1H), 1.62-1.51 (m, 3H), 1.50-1.41 (m, 2H), 1.37-1.26 (m, 3H). ^13^C NMR (126 MHz, DMSO-d6) δ 172.60, 169.46, 162.69, 150.20, 148.86, 139.60, 120.19, 113.83, 112.59, 84.54, 75.51, 61.04, 59.19, 55.33, 49.40, 44.00, 43.79, 36.67, 36.14, 33.53, 30.38, 27.99, 27.96, 24.55. LC-MS: calcd for C_25_H_32_N_2_O_5_S [M + H]^+^ = 473.2; found 473.2. HPLC purity: 100.00% (t_R_ = 21.557 min).

Synthetic schemes for compound Negative Prode-DCL

Reagents and conditions: (a) NaBH_4_, MeOH, 0 °C to rt, 2 h. (b) SeO_2_, TBHP, AcOH, DCM, rt, 12 h. (c) 5-hexynoyl chloride, pyridine, DMAP, 0 °C to rt,12 h.

DCL (920.0 mg, 4 mmol) was dissolved in anhydrous methanol (10 mL), and sodium borohydride (NaBH_4_, 303.0 mg, 8 mmol) was added portionwise under ice-cooling. The mixture was stirred at room temperature for 2 h, quenched with water (10 mL), and extracted with ethyl acetate (3 × 50 mL). The combined organic layers were washed with saturated brine (3 × 50 mL), dried over anhydrous sodium sulfate (Na_2_SO_4_), and concentrated under reduced pressure. The crude intermediate DCL-1 (835 mg, 3.6 mmol) was purified by silica gel column chromatography. DCL-1 was then dissolved in anhydrous dichloromethane (DCM, 1 mL), followed by sequential addition of selenium dioxide (SeO_2_, 8 mg, 0.072 mmol), tert-butyl hydroperoxide (TBHP, 5-6 M in decane, 4.32 mmol), and acetic acid (AcOH, 26.7 mg, 0.36 mmol). The reaction mixture was stirred at room temperature for 24 h, quenched with saturated aqueous sodium thiosulfate (Na_2_S_2_O_3_), and extracted with ethyl acetate (3 × 10 mL). The organic phase was washed with water (3 × 4 mL) and saturated brine (6 mL), dried over Na_2_SO_4_, and concentrated to afford intermediate DCL-2 after silica gel chromatography. Oxalyl chloride (990.6 mg, 7.8 mmol) was added to a solution of 5-hexynoic acid (291.85 mg, 2.6 mmol) in anhydrous DCM (5 mL) with one drop of dimethylformamide (DMF). After 1.5 h stirring, excess reagents were removed under reduced pressure. The residue was re-dissolved in anhydrous DCM (5 mL) at 0 °C, and pyridine (616.2 mg, 7.8 mmol), 4-dimethylaminopyridine (DMAP, catalytic), and DCL-2 (580.0 mg, 2.34 mmol) were sequentially added. Subsequent procedures were performed as previously described for Prode-DCL-1 synthesis, yielding Negative Prode-DCL (214.72 mg, 37% yield). This multi-step synthesis protocol was validated by ^1^H NMR tracking at each stage, with final compound purity confirmed as ≥95% by HPLC analysis.

Negative Prode-DCL: ^1^H NMR (500 MHz, CDCl3) δ 5.69 (t, J = 6.5 Hz, 1H), 5.49 (s, 1H), 5.38 (s, 1H), 4.93 (s, 1H), 4.78 (s, 1H), 3.90-3.84 (m, 1H), 3.07-2.99 (m, 2H), 2.57-2.50 (m, 1H), 2.46 (t, J = 7.4 Hz, 2H), 2.31-2.25 (m, 3H), 2.24-2.18 (m, 1H), 2.16-2.09 (m, 1H), 2.06-1.98 (m, 1H), 1.97 (q, J = 2.8 Hz, 2H), 1.95-1.90 (m, 1H), 1.89-1.83 (m, 2H), 1.47-1.39 (m, 1H), 1.28 (d, J = 3.8 Hz, 3H).^13^C NMR (126 MHz, CDCl3) δ 178.47, 172.89, 149.55, 148.85, 115.43, 112.89, 84.97, 83.35, 77.16, 76.20, 69.31, 50.23, 49.95, 44.48, 42.08, 37.95, 37.33, 33.33, 32.70, 23.78, 17.99, 13.40. LC-MS: calcd for C_21_H_26_O_4_ [M + H]^+^ = 343.2; found 343.2. HPLC purity: 100.00% (t_R_ = 25.953 min).

**Spectrum of compound**

^1^H NMR spectrum of DCL (500 MHz, CDCl3)


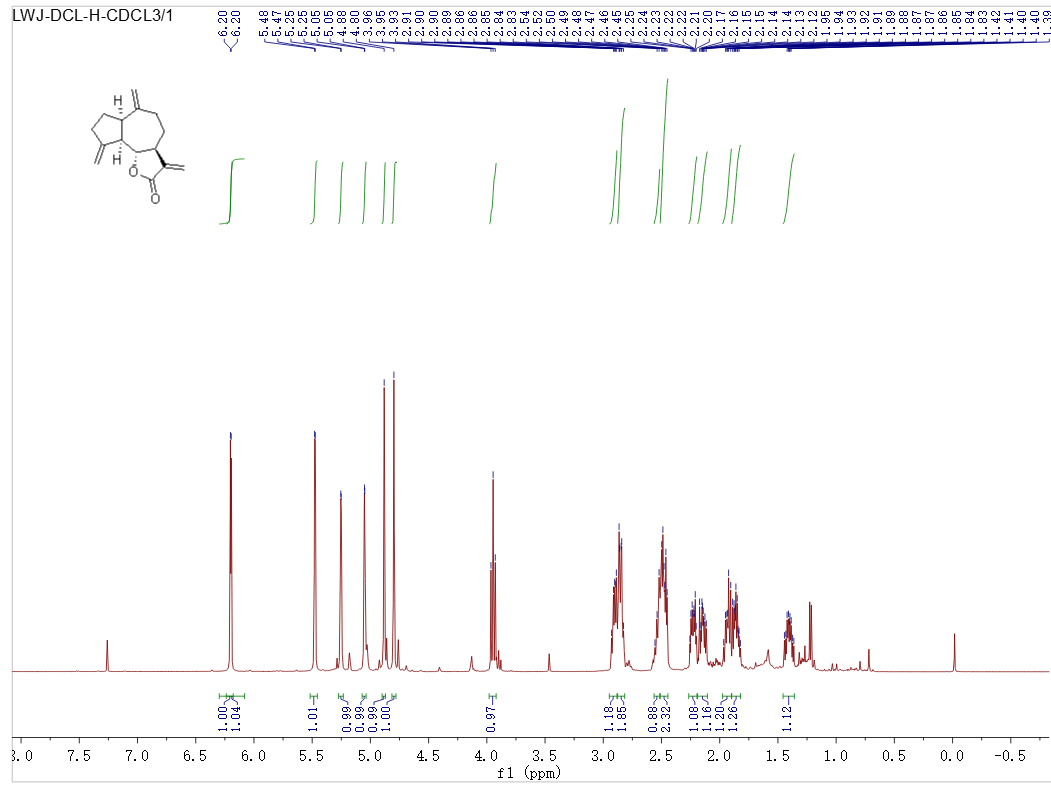


^13^C NMR spectrum of **DCL** (126 MHz, CDCl_3_)


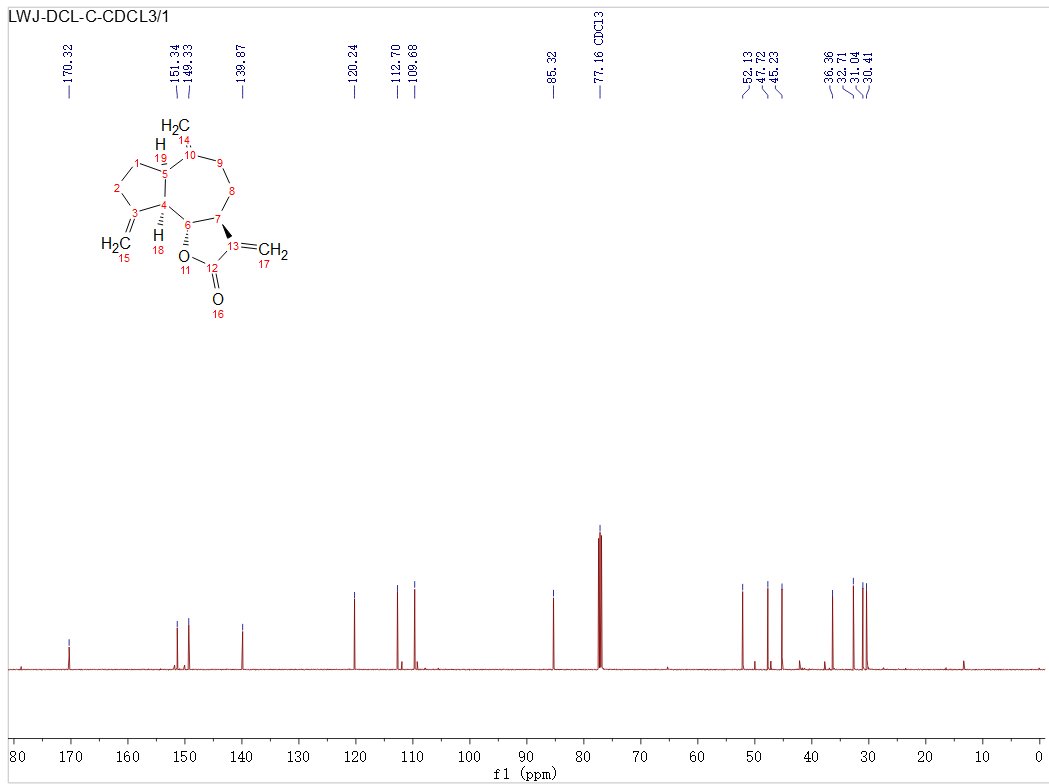


HPLC chromatograms of **DCL**


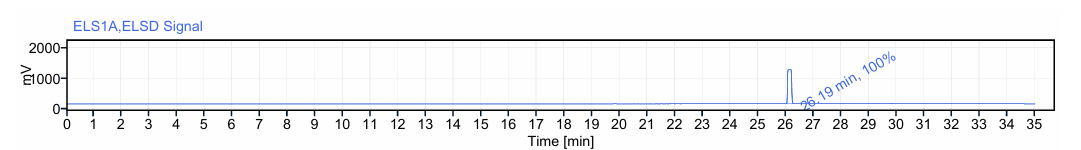


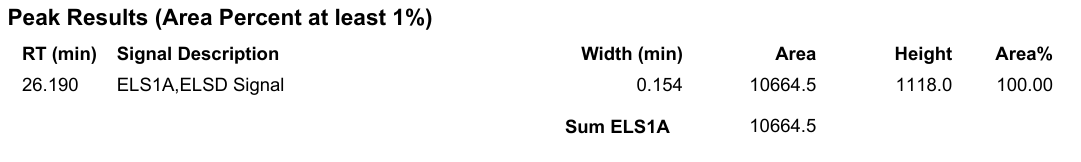

^1^H NMR spectrum of Int1 (500 MHz, CDCl_3_)


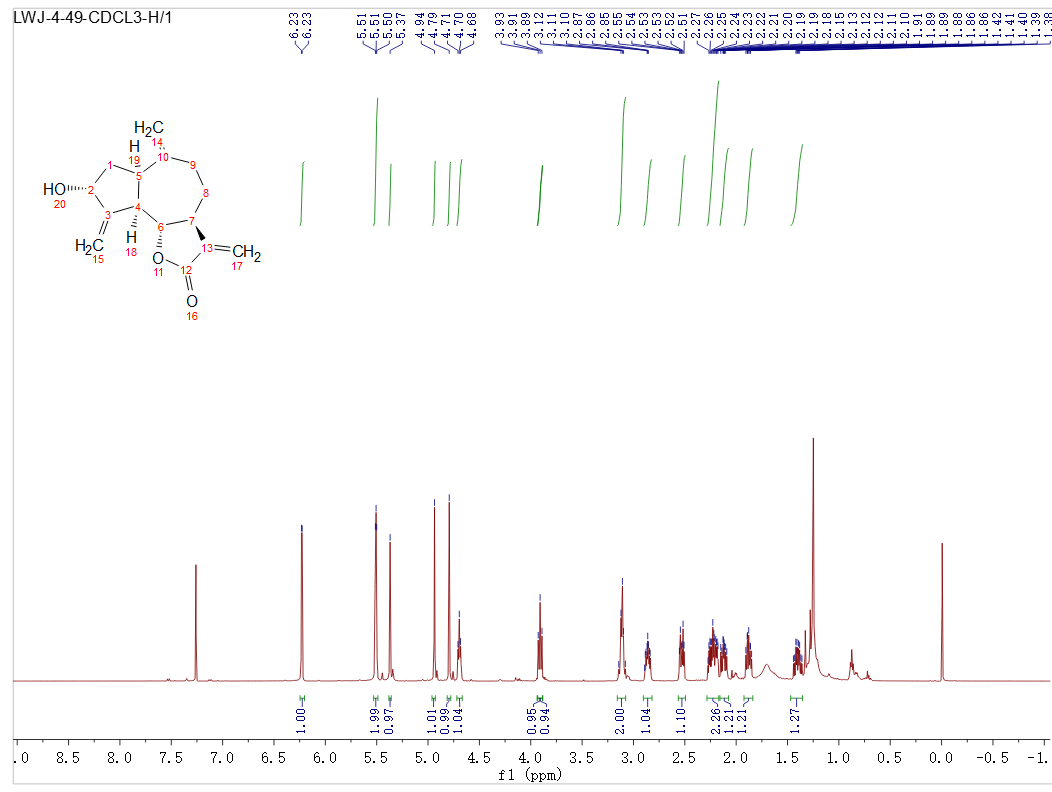


^13^C NMR spectrum of Int1 (126 MHz, CDCl_3_)


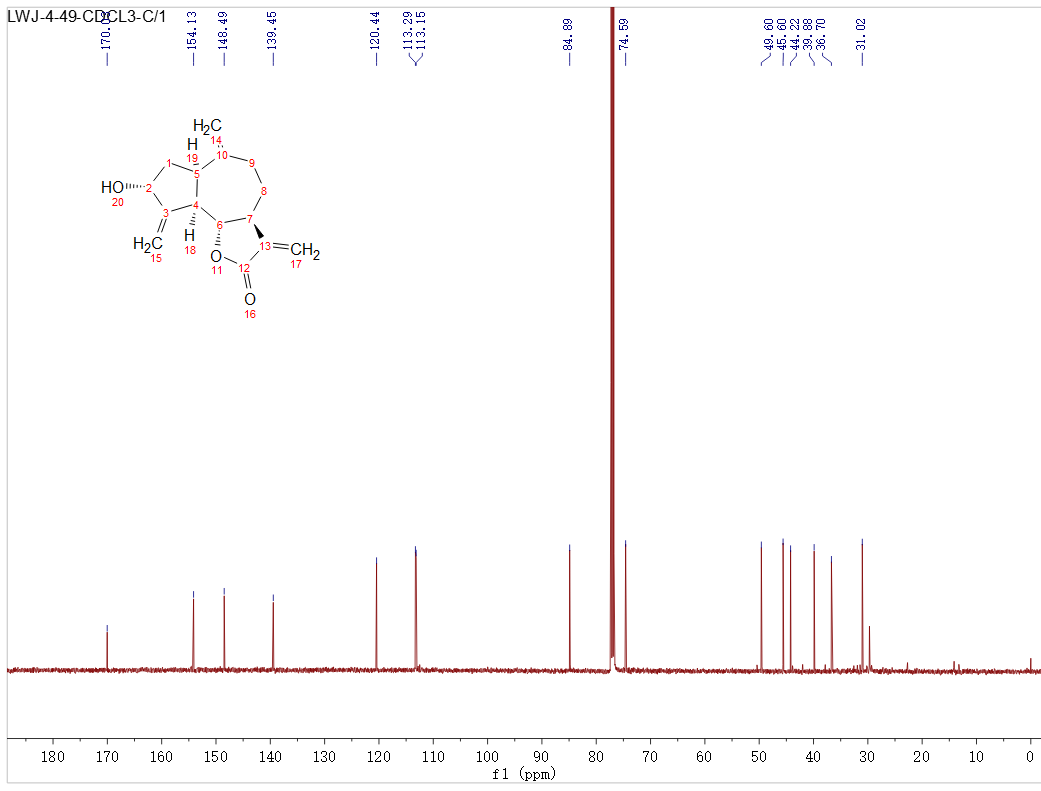


COSY NMR spectrum of Int1 (500 MHz, CDCl_3_)


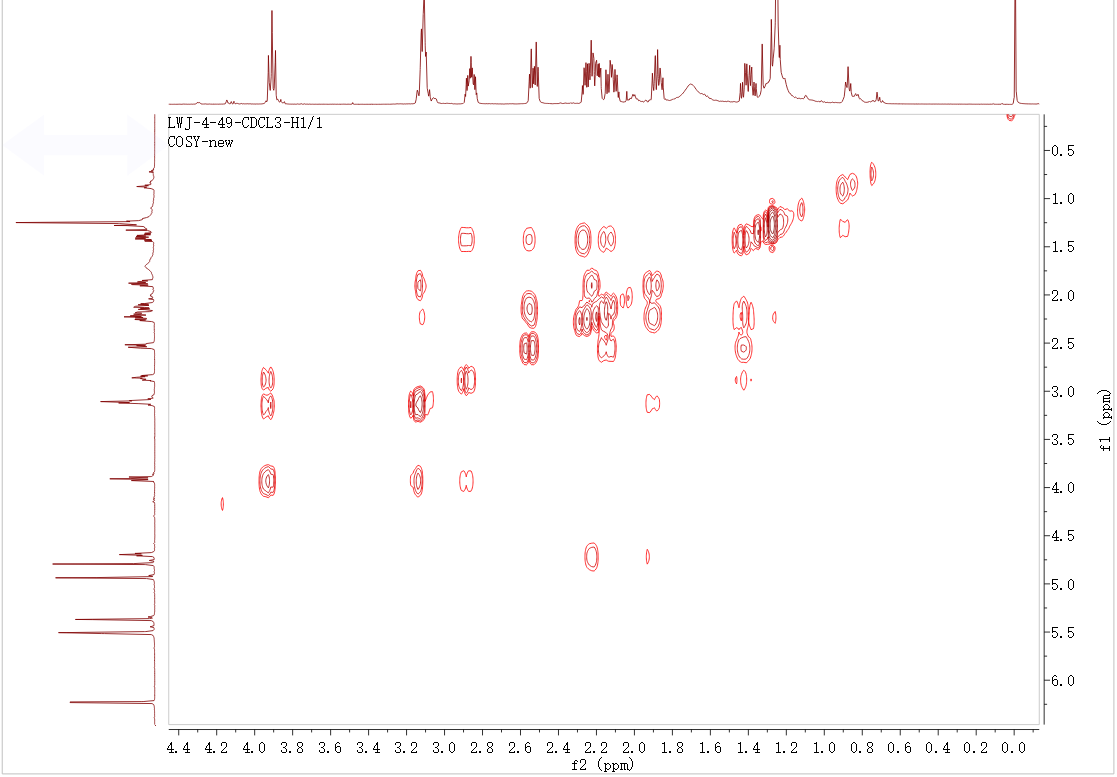


HSQC NMR spectrum of Int1 (500 MHz, CDCl_3_)


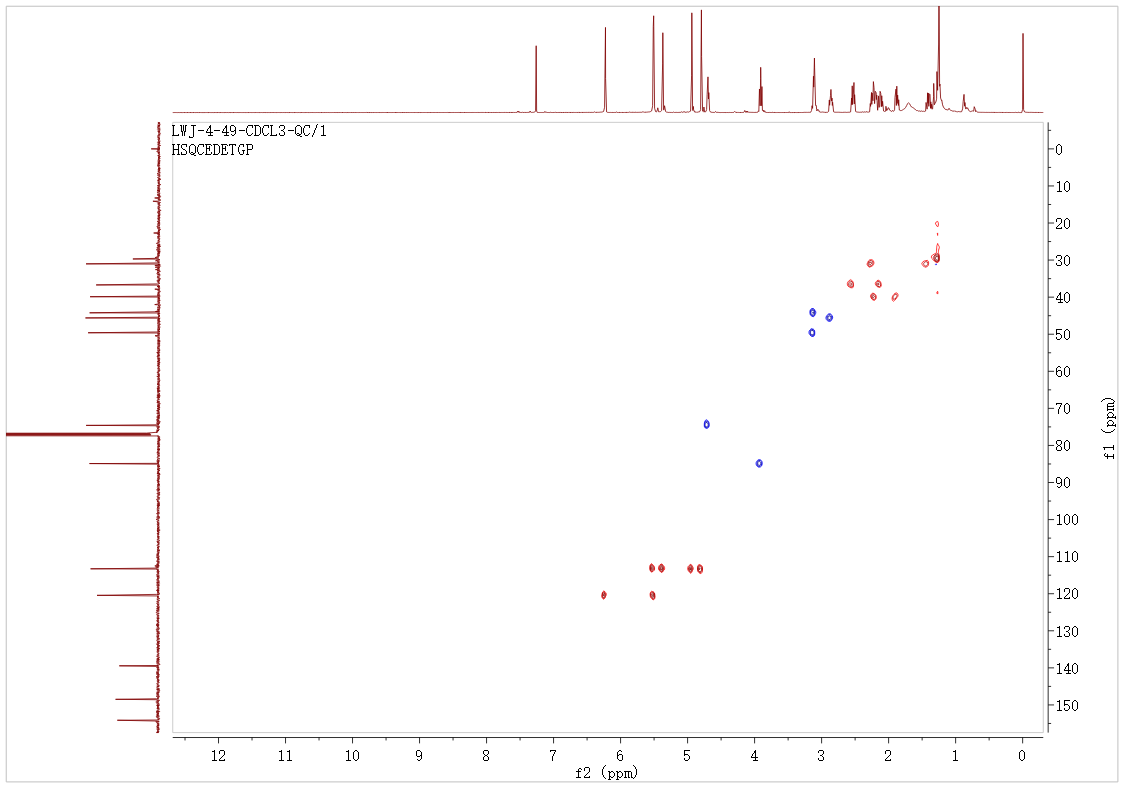


NOESY NMR spectrum of Int1 (500 MHz, CDCl_3_)


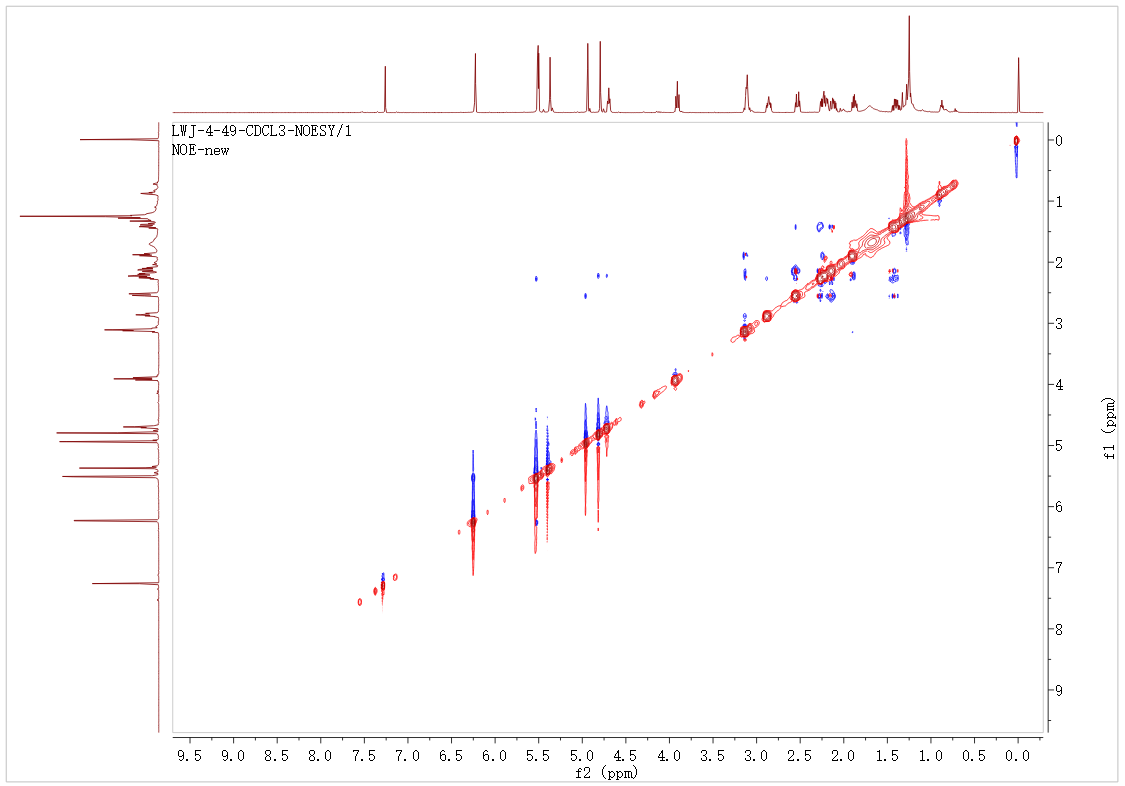


HPLC chromatograms of Int1


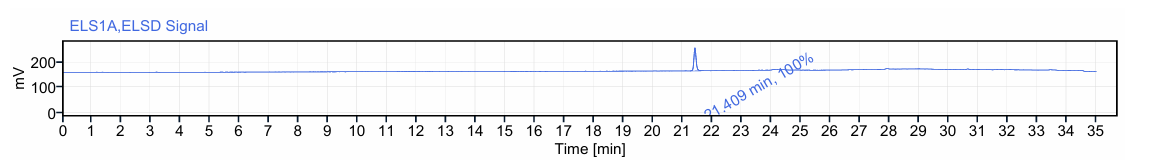


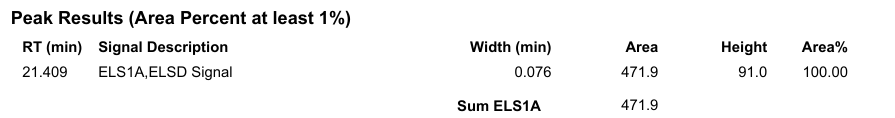

^1^H NMR spectrum of Prode-DCL-1 (500 MHz, CDCl_3_)


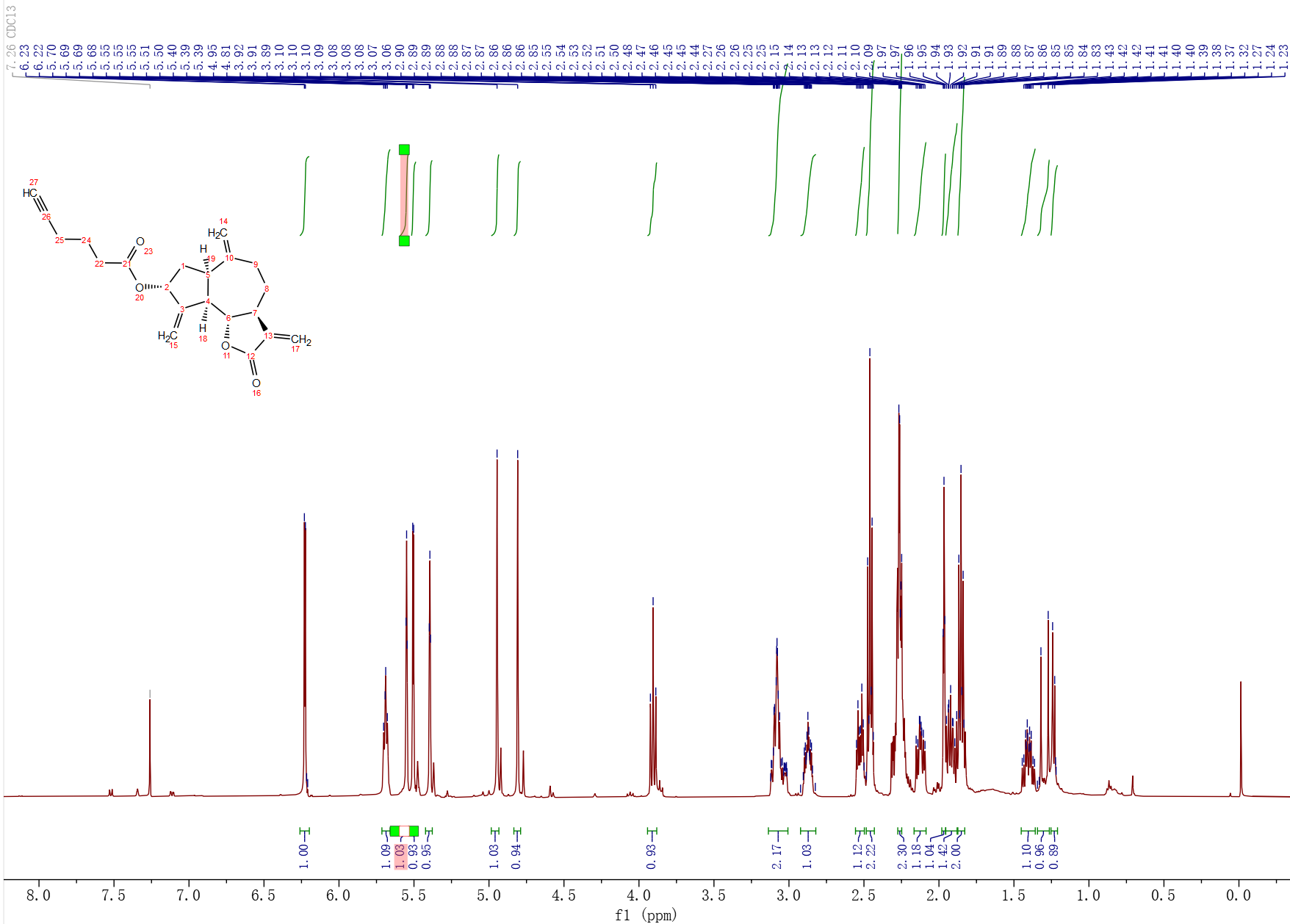


^13^C NMR spectrum of Prode-DCL-1 (126 MHz, CDCl_3_)


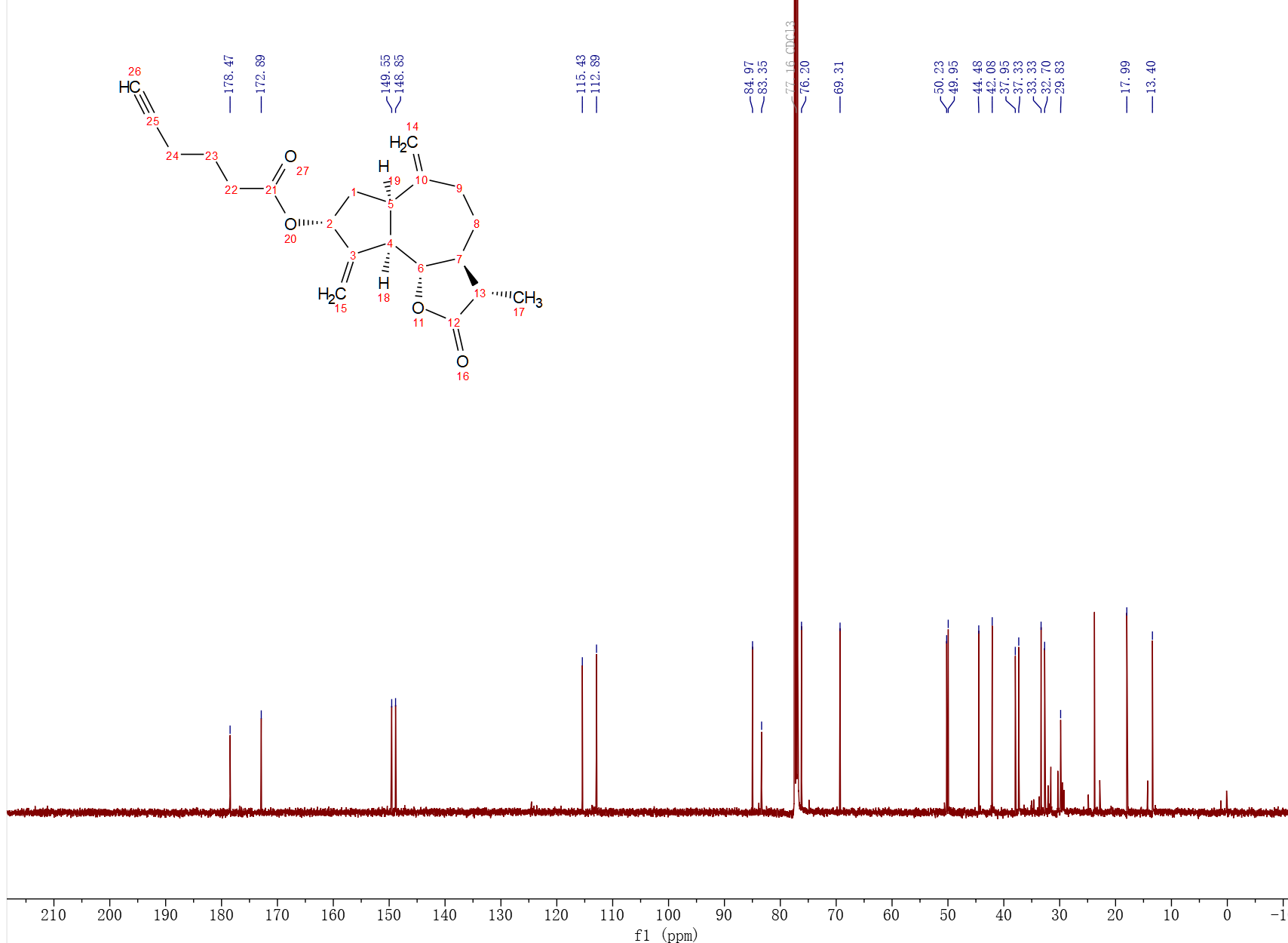


HPLC chromatograms of Prode-DCL-1


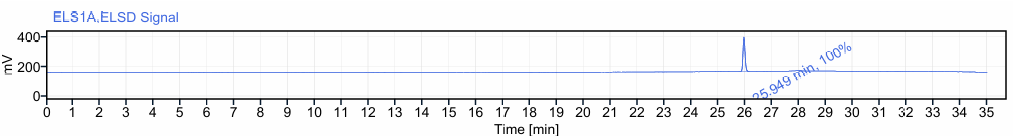


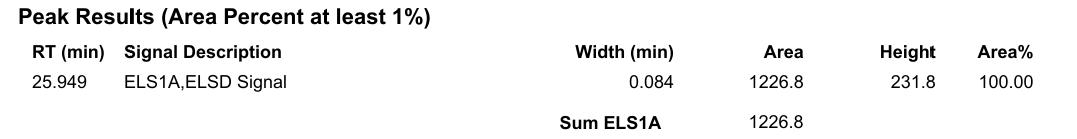

^1^H NMR spectrum of Prode-DCL-2 (500 MHz, CDCl_3_)


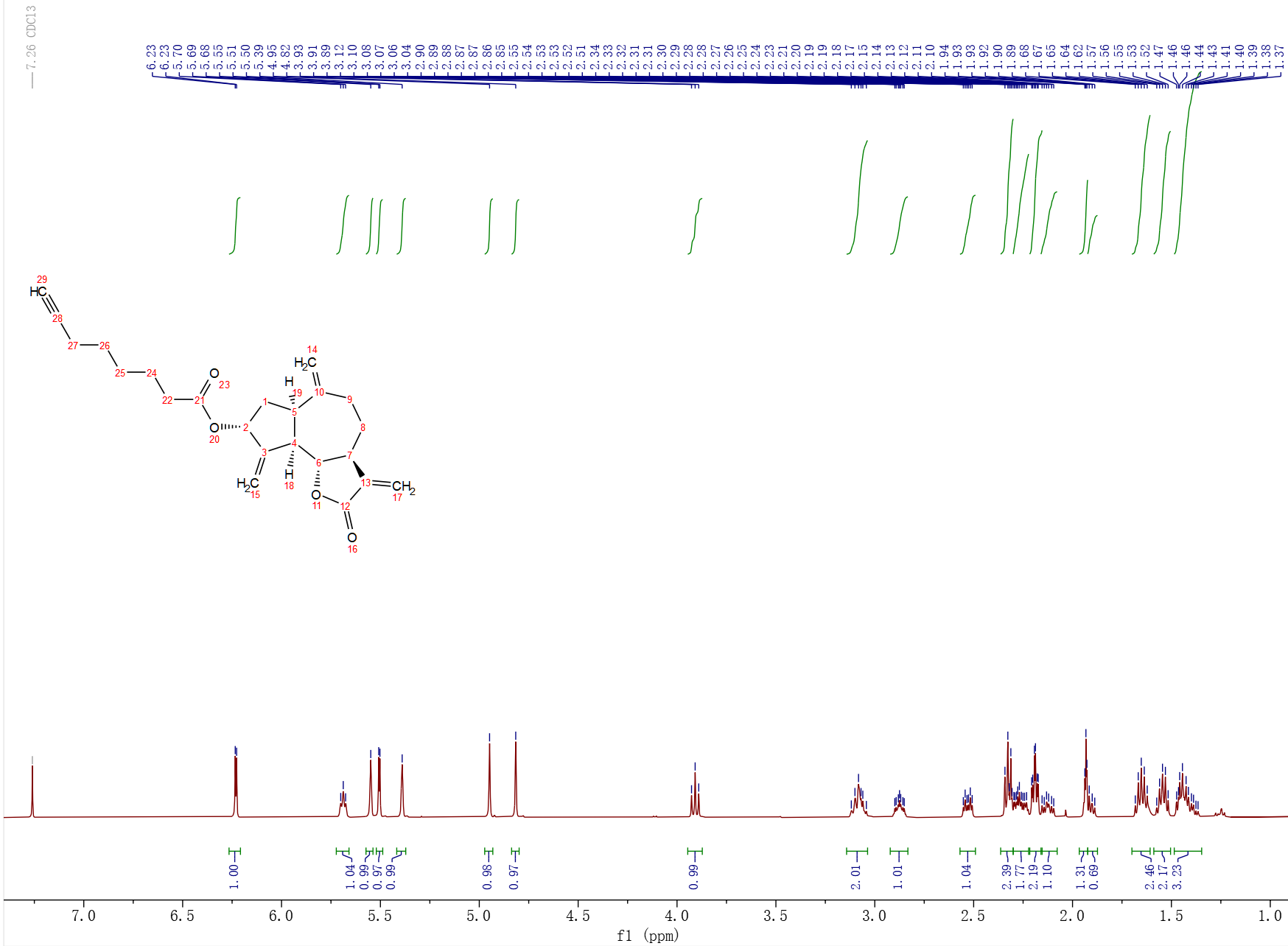


^13^C NMR spectrum of Prode-DCL-2 (126 MHz, CDCl_3_)


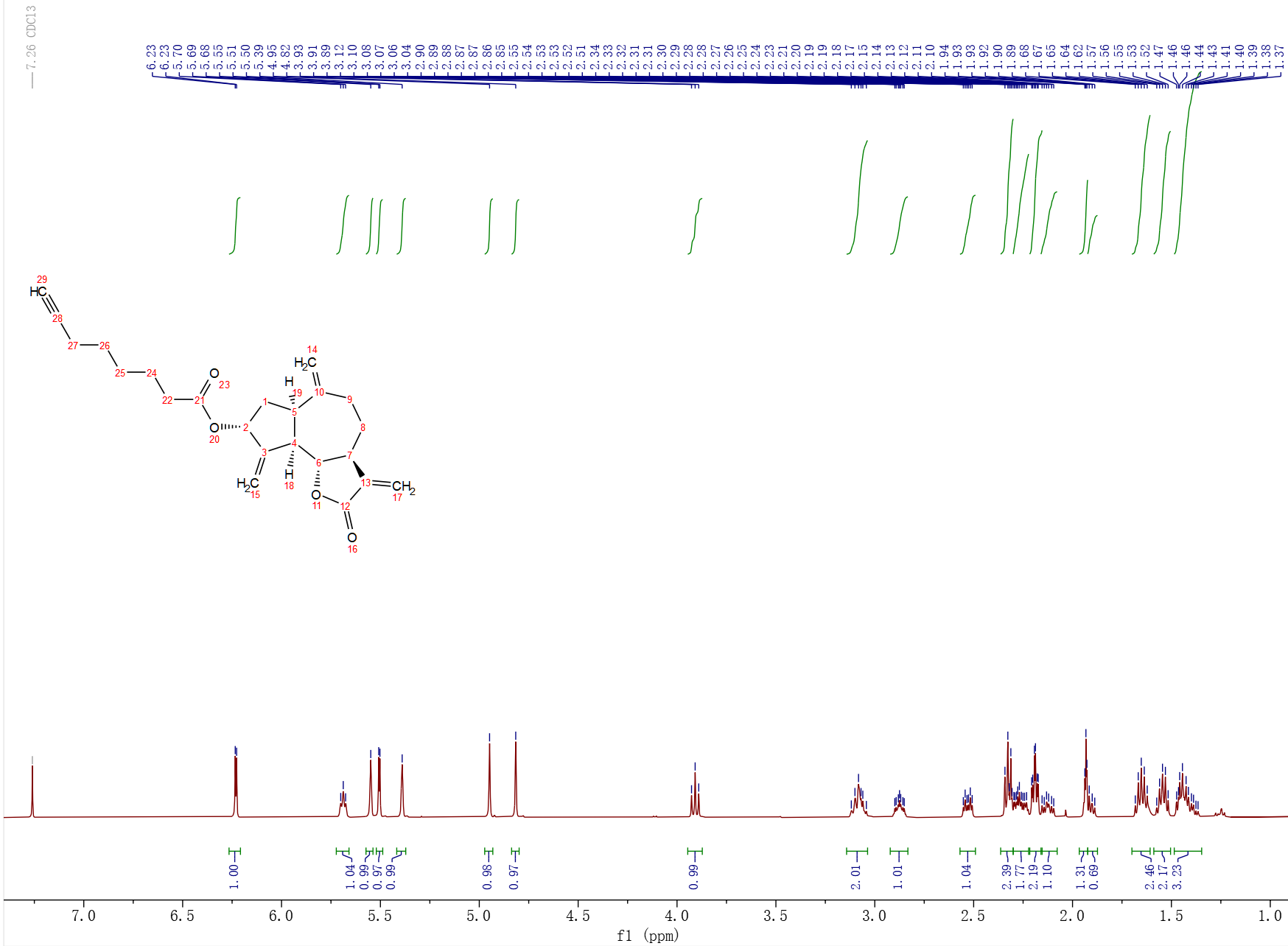


HPLC chromatograms of Prode-DCL-2


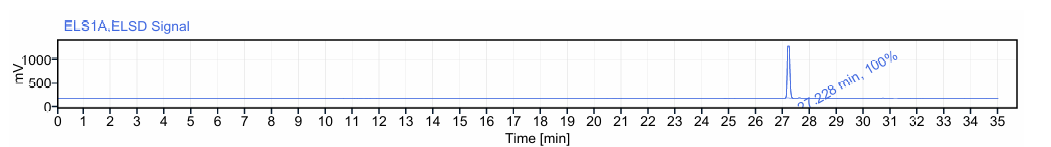


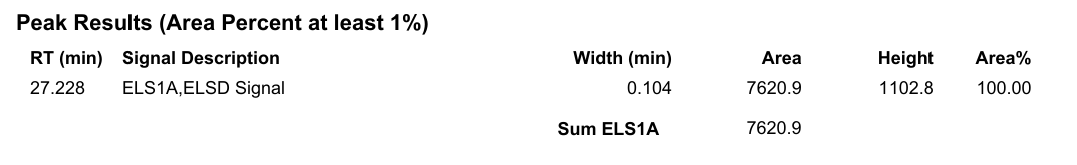

^1^H NMR spectrum of Prode-DCL-3 (500 MHz, CDCl_3_)

1
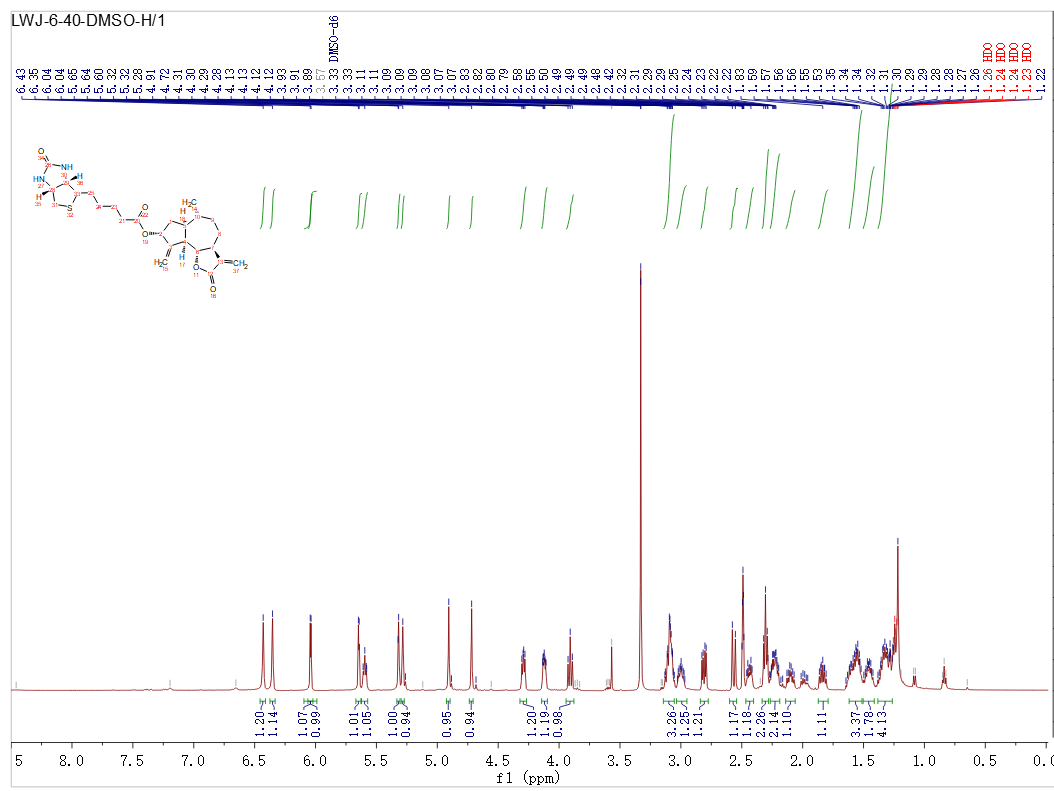


^13^C NMR spectrum of Prode-DCL-3 (126 MHz, CDCl_3_)


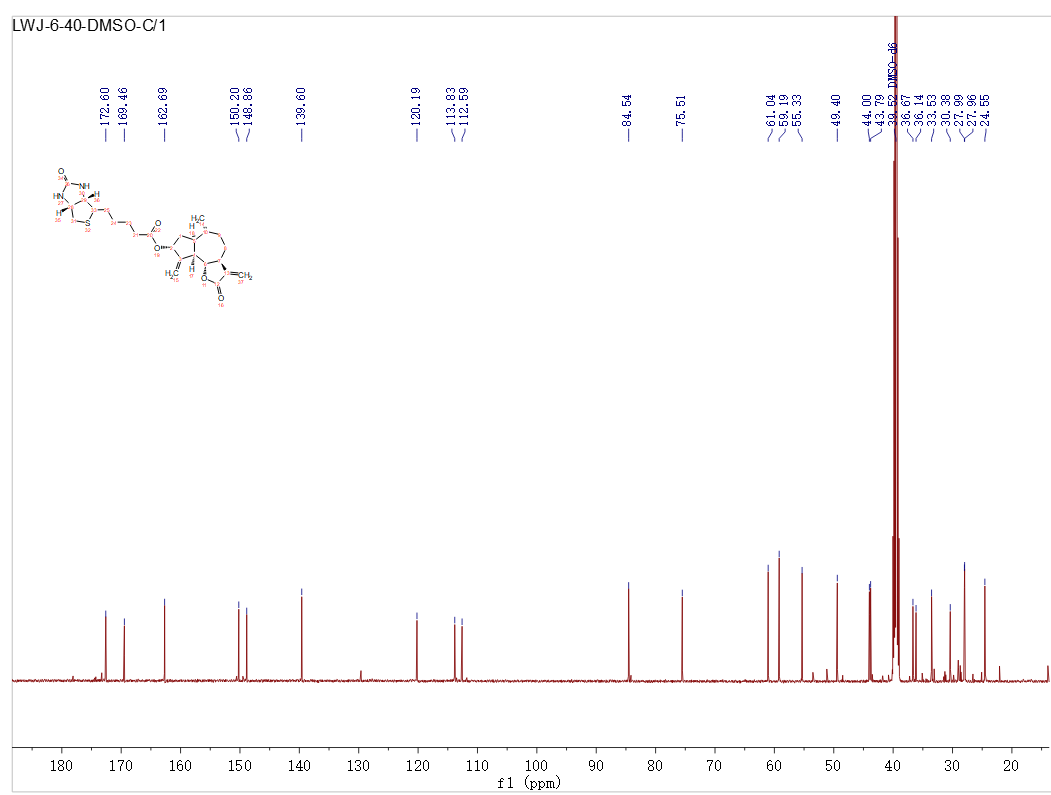


HPLC chromatograms of Prode-DCL-3


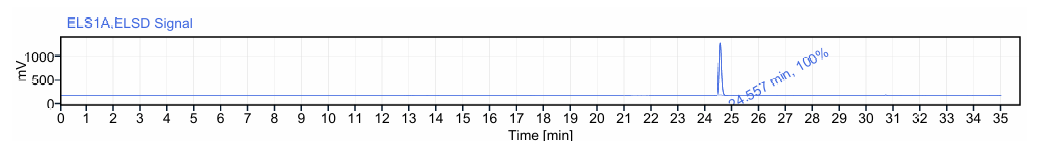


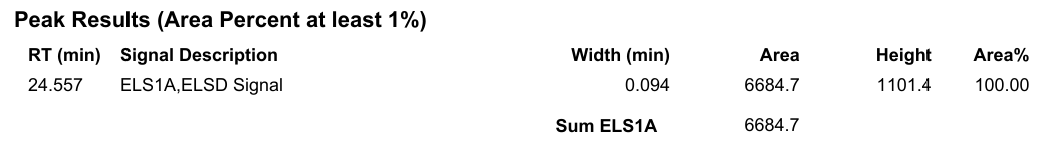

^1^H NMR spectrum of DCL-1 (500 MHz, CDCl_3_)


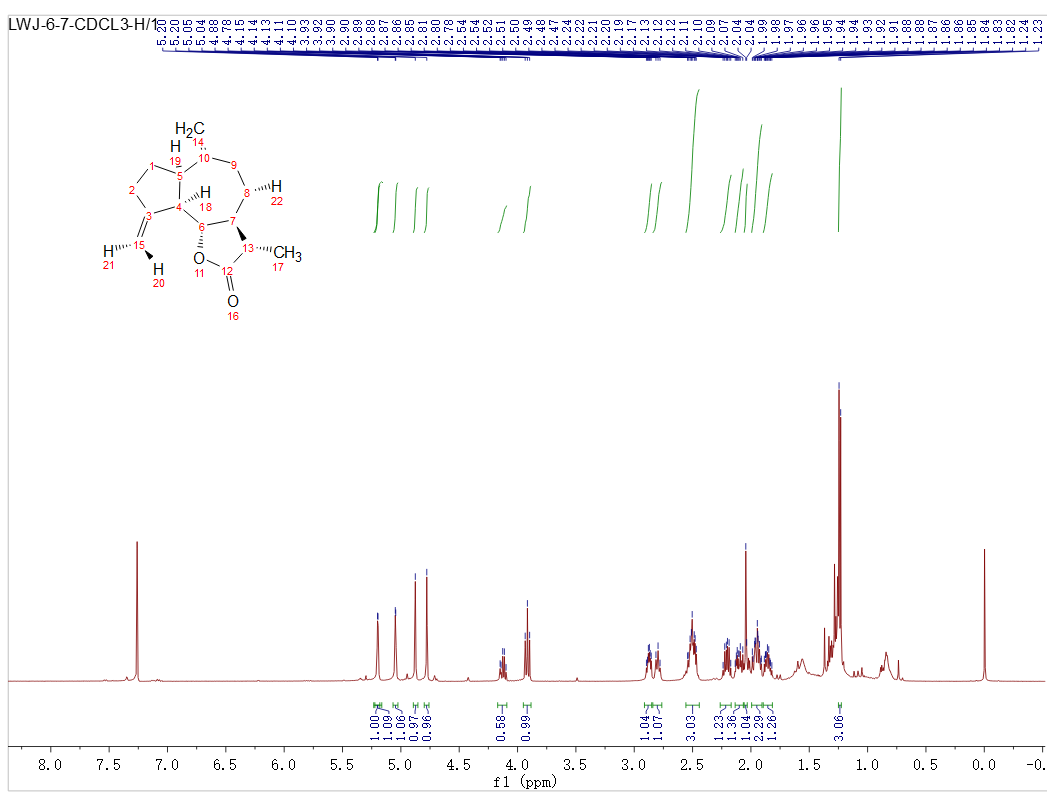


^13^C NMR spectrum of DCL-1 (126 MHz, CDCl_3_)


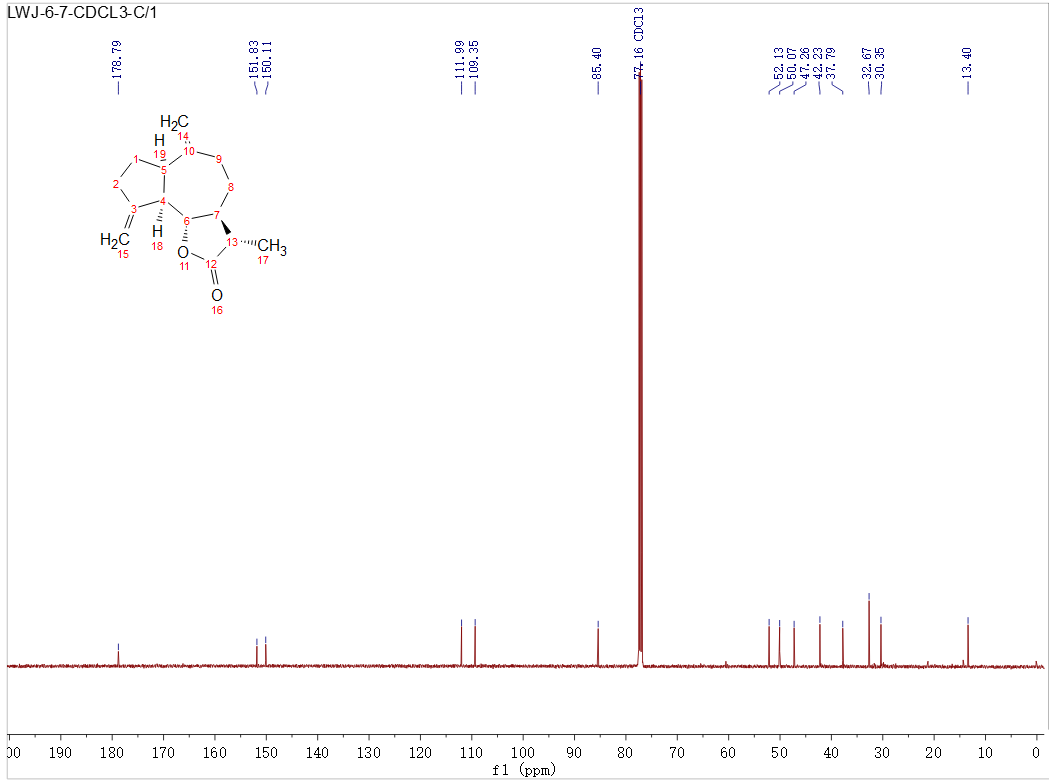


COSY NMR spectrum of DCL-1 (500 MHz, CDCl_3_)


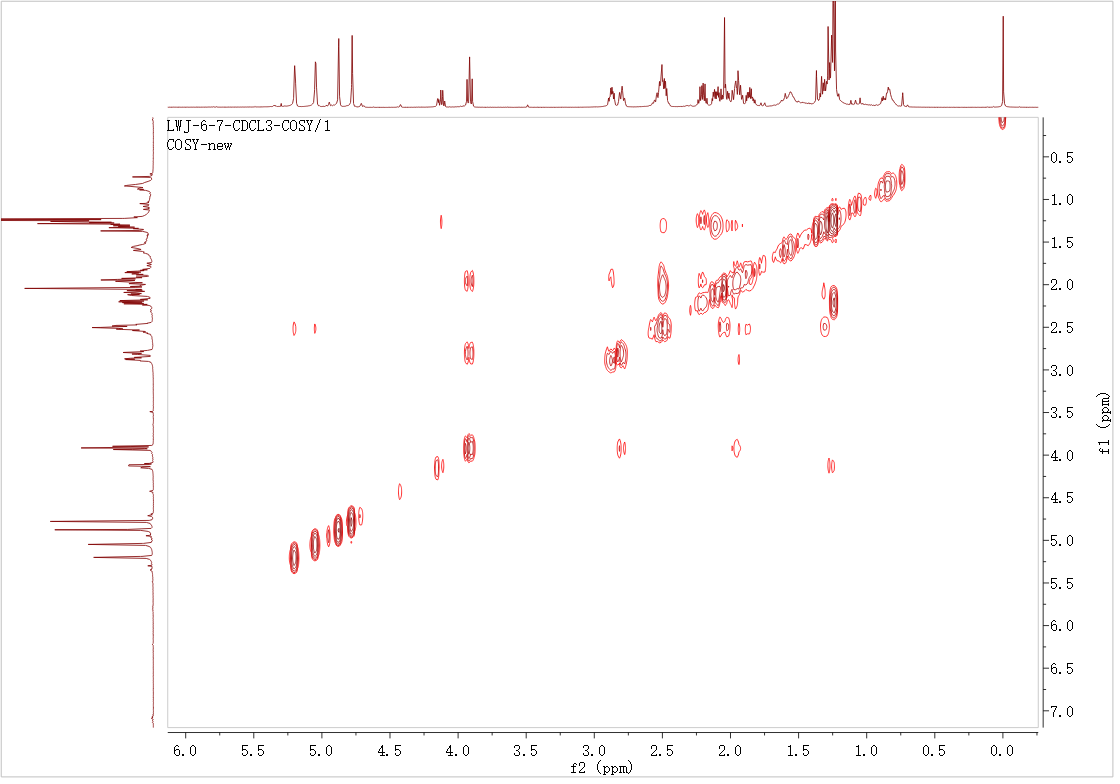


HSQC NMR spectrum of DCL-1 (500 MHz, CDCl_3_)


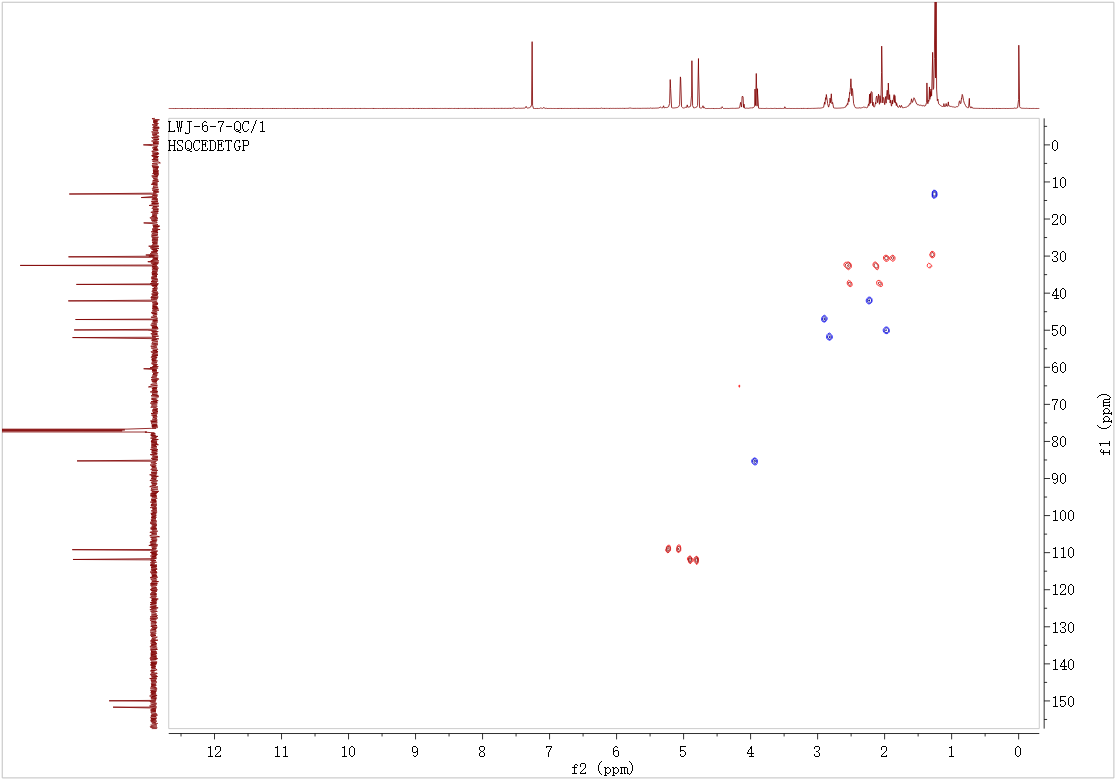


NOESY NMR spectrum of DCL-1 (500 MHz, CDCl_3_)


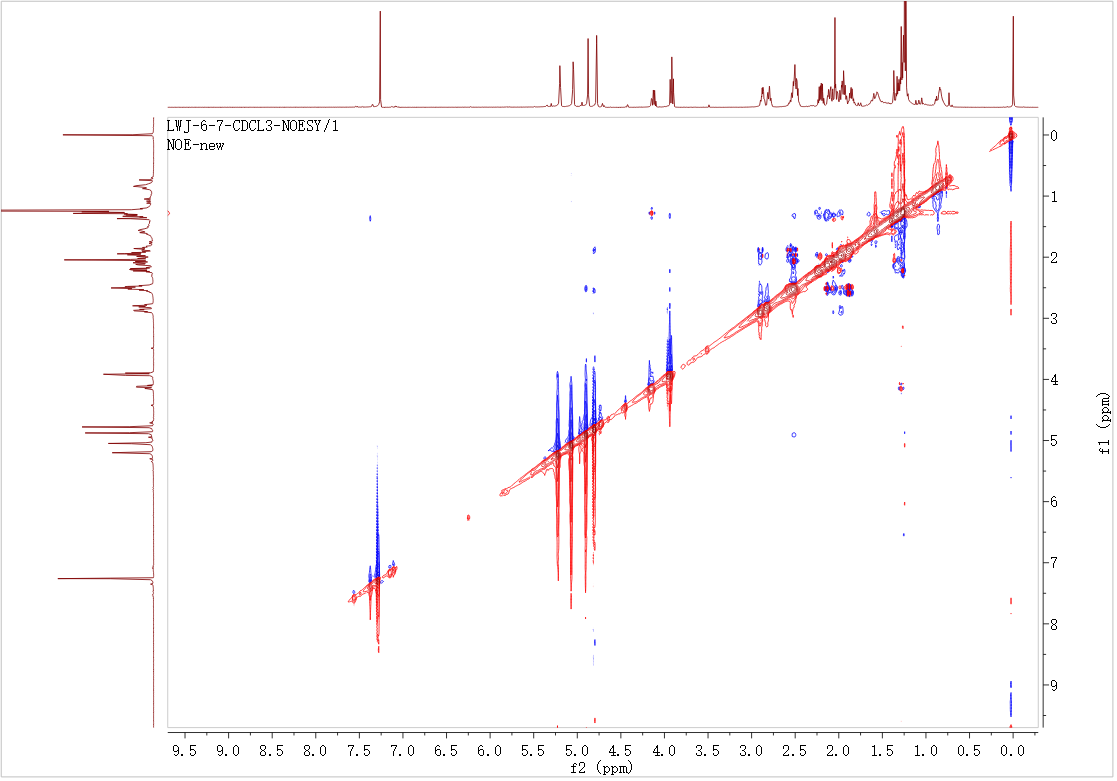


HPLC chromatograms of DCL-1


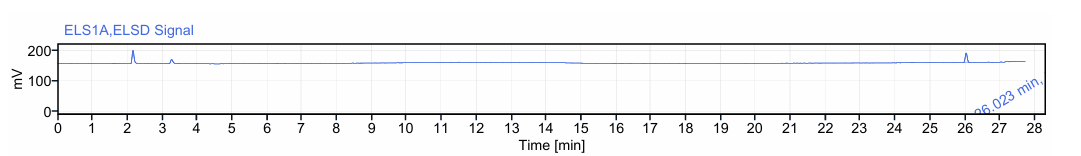


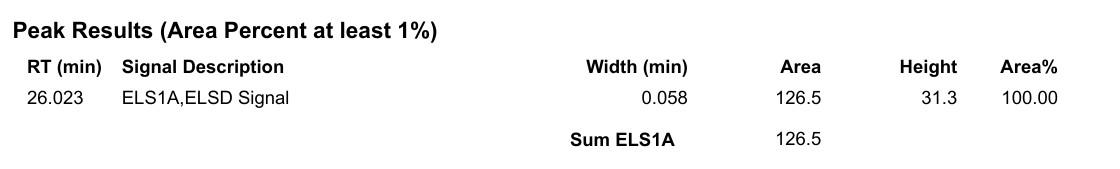

^1^H NMR spectrum of Negative Prode-DCL (500 MHz, CDCl_3_)


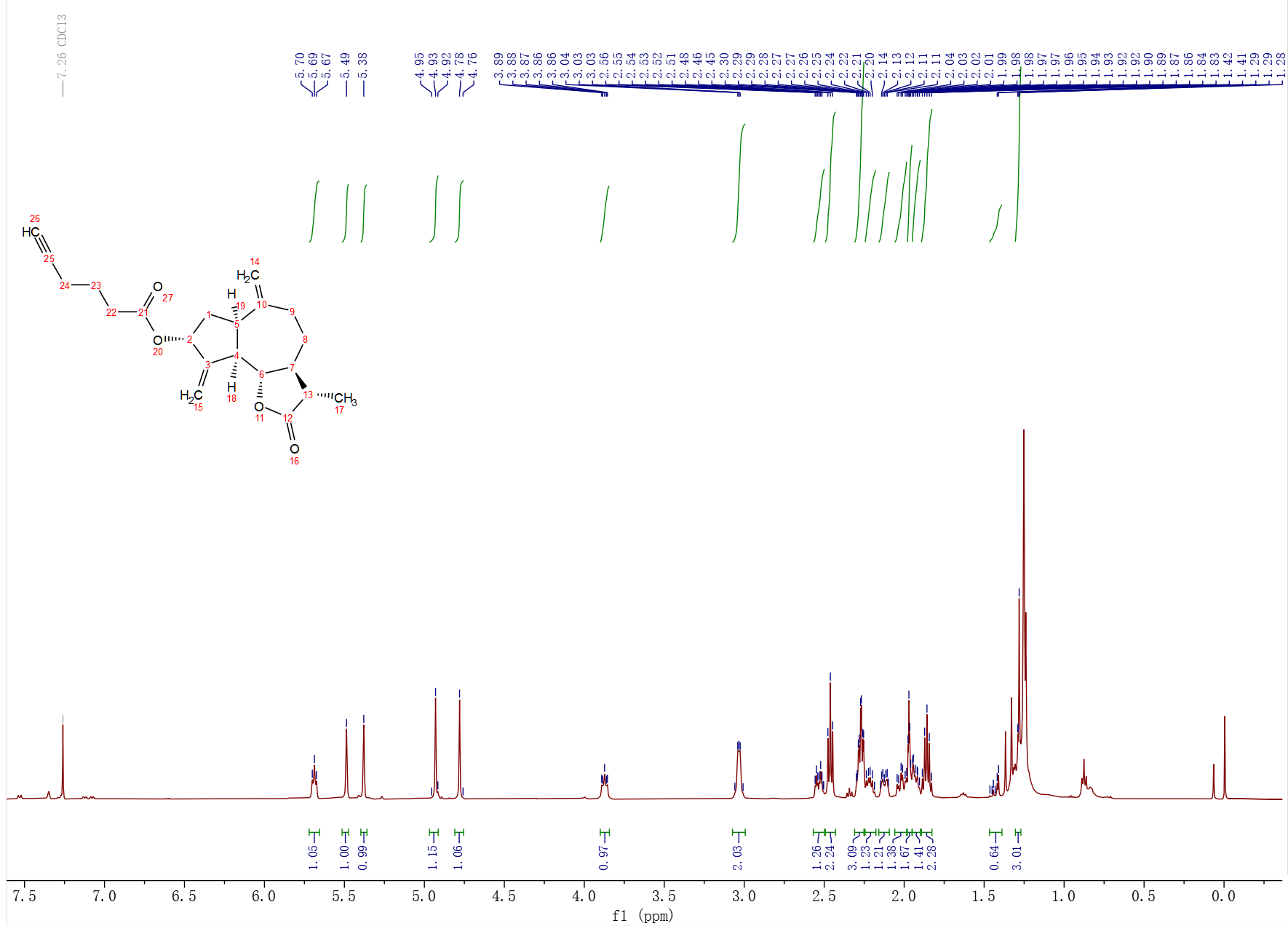


^13^C NMR spectrum of Negative Prode-DCL (126 MHz, CDCl_3_)


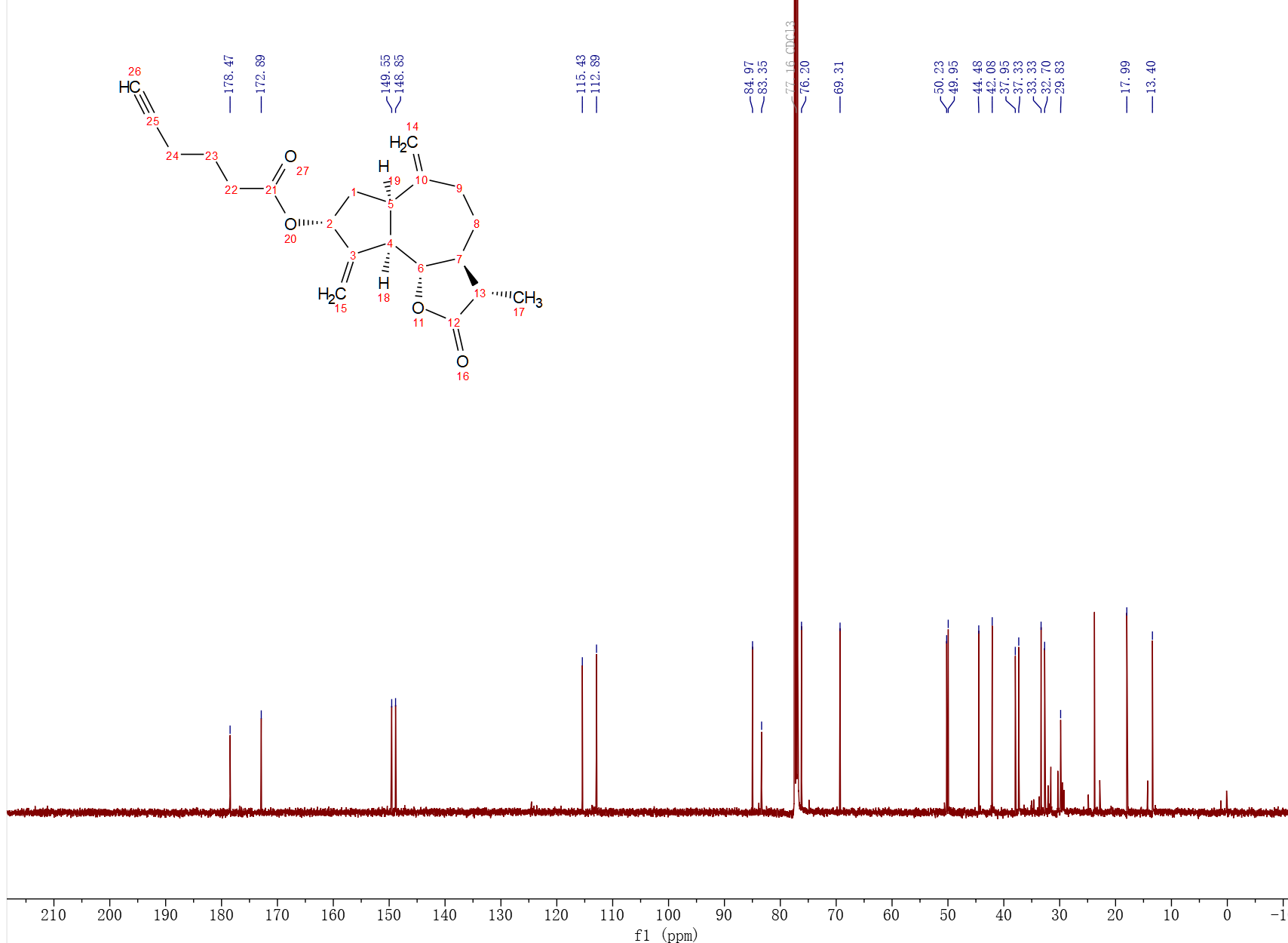


HPLC chromatograms of Negative Prode-DCL


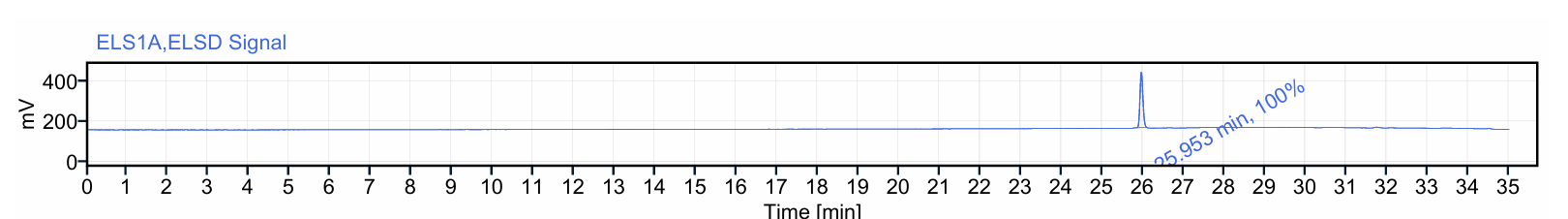


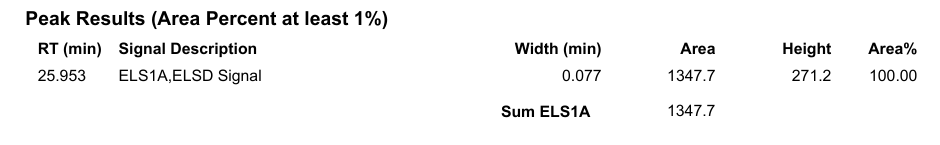

Supplement: Supplementary file 1 — Supporting File: advs74192‐sup‐0001‐SuppMat.docx [file ADVS-13-e20277-s001.docx]
